# Synthesis of pyrimidine-6-carbonitriles, pyrimidin-5-ones, and tetrahydroquinoline-3-carbonitriles by new superb oxovanadium(V)-[5,10,15,20-tetrakis(pyridinium)-porphyrinato]-tetra(tricyanomethanide) catalyst via anomeric based oxidation

**DOI:** 10.1038/s41598-022-23956-6

**Published:** 2022-11-14

**Authors:** Mohammad Dashteh, Sajjad Makhdoomi, Saeed Baghery, Mohammad Ali Zolfigol, Ardeshir Khazaei, Yanlong Gu

**Affiliations:** 1 Department of Organic Chemistry, Faculty of Chemistry, Bu-Ali Sina University, Hamedan, 6517838683 Iran; 2 Department of Pharmacology and Toxicology, School of Pharmacy, Hamedan University of Medicinal Science, Hamedan, Iran; 3 School of Chemistry and Chemical Engineering, Huazhong University of Science and Technology, 1037 Luoyu Road, Hongshan District, Wuhan, 430074 People’s Republic of China

**Keywords:** Chemistry, Catalysis, Green chemistry, Organic chemistry

## Abstract

Oxovanadium(V)-[5,10,15,20-tetrakis(pyridinium)-porphyrinato]-tetra(tricyanomethanide) [(VO)TPP][(TCM)_4_] was designed, synthesized and characterized by various techniques such as FT-IR, EDX, SEM equipped with EDX mappings, CHN elemental analysis, ICP-OES, XRD, SEM, TEM, TGA, DTA, DRS, Kubelka–Munk function (Tauc’s plot), and UV–Vis analyses. Then, [(VO)TPP][(TCM)_4_] was used as a benign and expedient catalyst for the synthesis of numerous heterocyclic compounds such as 5-amino-7-(aryl)-4,7-dihydro-[1,2,4]triazolo[1,5-*a*]pyrimidine-6-carbonitriles, 5-amino-7-(aryl)-[1,2,4]triazolo[1,5-*a*]pyrimidine-6-carbonitriles, 7-(aryl)-7,12-dihydro-5*H*-isochromeno[4,3-*d*][1,2,4]triazolo[1,5-*a*]pyrimidin-5-ones, and 4-(aryl)-2-(1*H*-indol-3-yl)-5,6,7,8-tetrahydroquinoline-3-carbonitriles under solvent-free conditions at 100 °C via a cooperative geminal-vinylogous anomeric based oxidation.

## Introduction

Triazolopyrimidines are among the great substantial hybrid heterocycles of pyrimidine as they are structural fundamentals of bioactive natural target molecules^1,2^. These compounds have engrossed much consideration from agricultural and medicinal scientists because of their varied range of biological activities, such as bromodomain inhibitors^3^, leishmanicidal^4^, receptor antagonists^5,6^, herbicidal^7,8^, antimalarial^9,10^, cardiovascular vasodilators^11^, anti-inflammatory^12^, and fungicidal^13^ factors.

Polycyclic compounds have engrossed great investigation attention because of their key functional organic materials including solar cells, light-emitting devices, and semiconductors^14–16^. Tetrahydroquinoline skeleton has established substantial attention not only as a significant building block but also as a structural part of the pharmaceutical targets^17–19^. These compounds are identified to occur in several natural products for example alkaloids establish in fungal sources, rutaceous and non-rutaceous plants^17–21^, and it is also employed in the design of numerous synthetic compounds with various exciting and advanced pharmacological properties^22^.

Triazolopyrimidines and tetrahydroquinolines were synthesized by using numerous approaches and catalysts such as thiamine hydrochloride (VB_1_)^23^, Nafion-H^24^, phthalhydrazide-MCM-41 (P-MCM-41)^25^, boric acid under aqueous micellar medium^26^, [DABCO](SO_3_H)_2_Cl_2_ and [DABCO](HSO_3_)_2_(HSO_4_)_2_^27^, DBU^28^, microwave irradiation^29^, etc.^30–39^.

Porphyrin and phthalocyanine compounds have established various applications in the part of material science owing to their distinguished optical and electrical properties in addition to their thermal stability. These complexes of transition metals are noteworthy as potential oxidation catalysts due to their rather inexpensive and efficient preparation on a large scale and their chemical and thermal stability. Their macrocyclic structure is extensively employed by nature in the active sites of oxygenase enzymes. These compounds found an extraordinarily useful category of compounds with various technological uses^40–50^. Among antibacterial and antifungal agents porphyrin complexes attract consideration because of their ability to act as photosensitizers. Some porphyrins were established to display phototoxicity to bacteria and fungi^51–55^. Porphyrins and metal porphyrins having a positive charge are superb photosensitizers for the photodynamic inactivation of micro-organisms, especially, Escherichia coli^56,57^.

**Figure 1:**
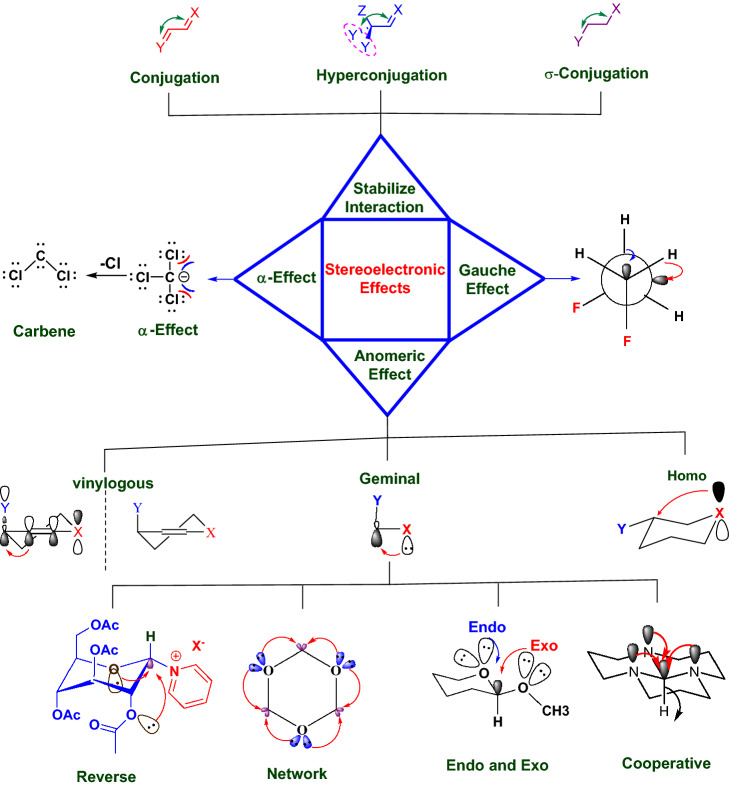
Various kinds of stereoelectronic interactions^63^.

The appropriate application of the anomeric effect in synthetic approaches may lead to noteworthy stereoselective reactions^58–62^. According to Alabugin’s theory, stereoelectronic effects are a bridge between structure and reactivity^63^. The anomeric effect as a famous subset of stereoelectronic interactions has been categorized into endo, exo, homo, geminal, reverse, and vinylogous (Fig. 1). Recently, we have reviewed the role of the anomeric effect concepts broadly^64^.

**Figure 2:**
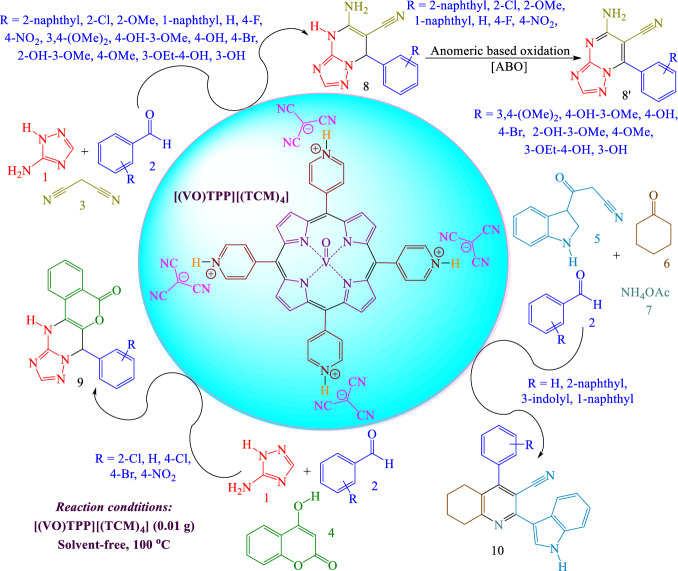
Synthesis of triazolopyrimidines and tetrahydroquinolines by using [(VO)TPP][(TCM)_4_] as a catalyst.

**Figure 3:**
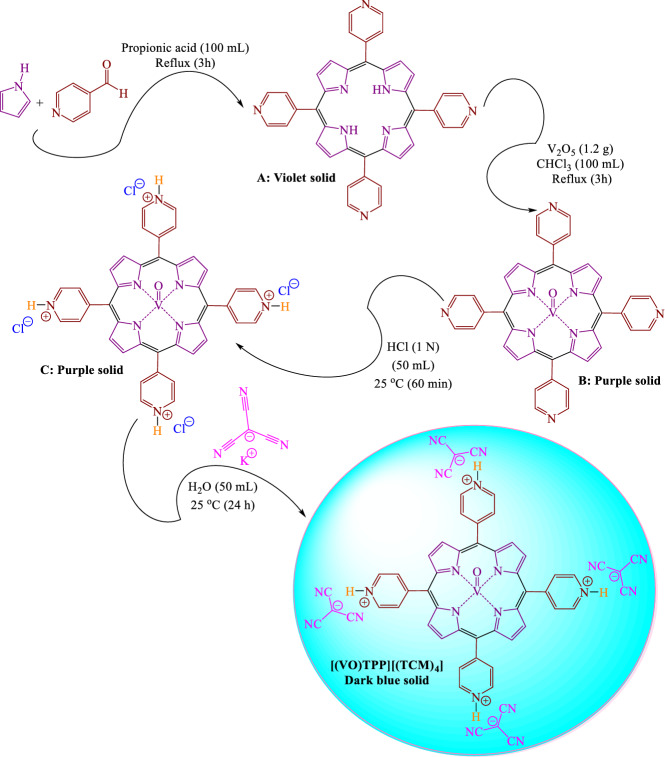
Synthesis of [(VO)TPP][(TCM)_4_].

To continue our investigations in this field^65–70^, herein, we synthesized numerous pyrimidine-6-carbonitrile, pyrimidin-5-one, and tetrahydroquinoline-3-carbonitrile compounds via a cooperative geminal-vinylogous anomeric based oxidation under solvent-free conditions at 100 °C (Fig. 2) by using oxovanadium(V)-[5,10,15,20-tetrakis(pyridinium)-porphyrinato]-tetra(tricyanomethanide) [(VO)TPP][(TCM)_4_] as a versatile catalyst (Fig. 3).

## Results and discussion

Design, synthesis, and applications of porphyrins and phthalocyanines in the synthesis of various catalysts are our great interest^71–76^. In this regard, we wish to report a new organic–inorganic hybrid of porphyrin, oxovanadium(V)-[5,10,15,20-tetrakis(pyridinium)-porphyrinato]-tetra(tricyanomethanide) [(VO)TPP][(TCM)_4_]. Also, we decided to synthesize numerous pyrimidine-6-carbonitrile, pyrimidin-5-one, and tetrahydroquinoline-3-carbonitrile compounds in the presence of described catalyst due to their biological activity and abilities for the development of anomeric based oxidation (ABO).

### Characterization of oxovanadium(V)-[5,10,15,20-tetrakis(pyridinium)-porphyrinato]-tetra(tricyanomethanide) [(VO)TPP][(TCM)_4_]

The structure of [(VO)TPP][(TCM)_4_] was investigated by using numerous analyses such as FT-IR, energy-dispersive X-ray spectroscopy (EDX), scanning electron microscopy (SEM)-coupled EDX (SEM equipped with EDX mappings), CHN elemental analysis, inductively coupled plasma-optical emission spectrometry (ICP-OES), X-ray diffraction (XRD), scanning electron microscopy (SEM), transmission electron microscopy (TEM), thermal gravimetric analysis (TGA), differential thermal analysis (DTA), diffuse reflectance spectroscopy (DRS), Kubelka–Munk function (Tauc’s plot), and UV–Vis. Refer to the supplementary information section to study the FTIR, EDX, TGA, DTA, DRS, Kubelka–Munk function, and UV–Vis of the catalyst (Figs.3S and 7S).

**Figure 4:**
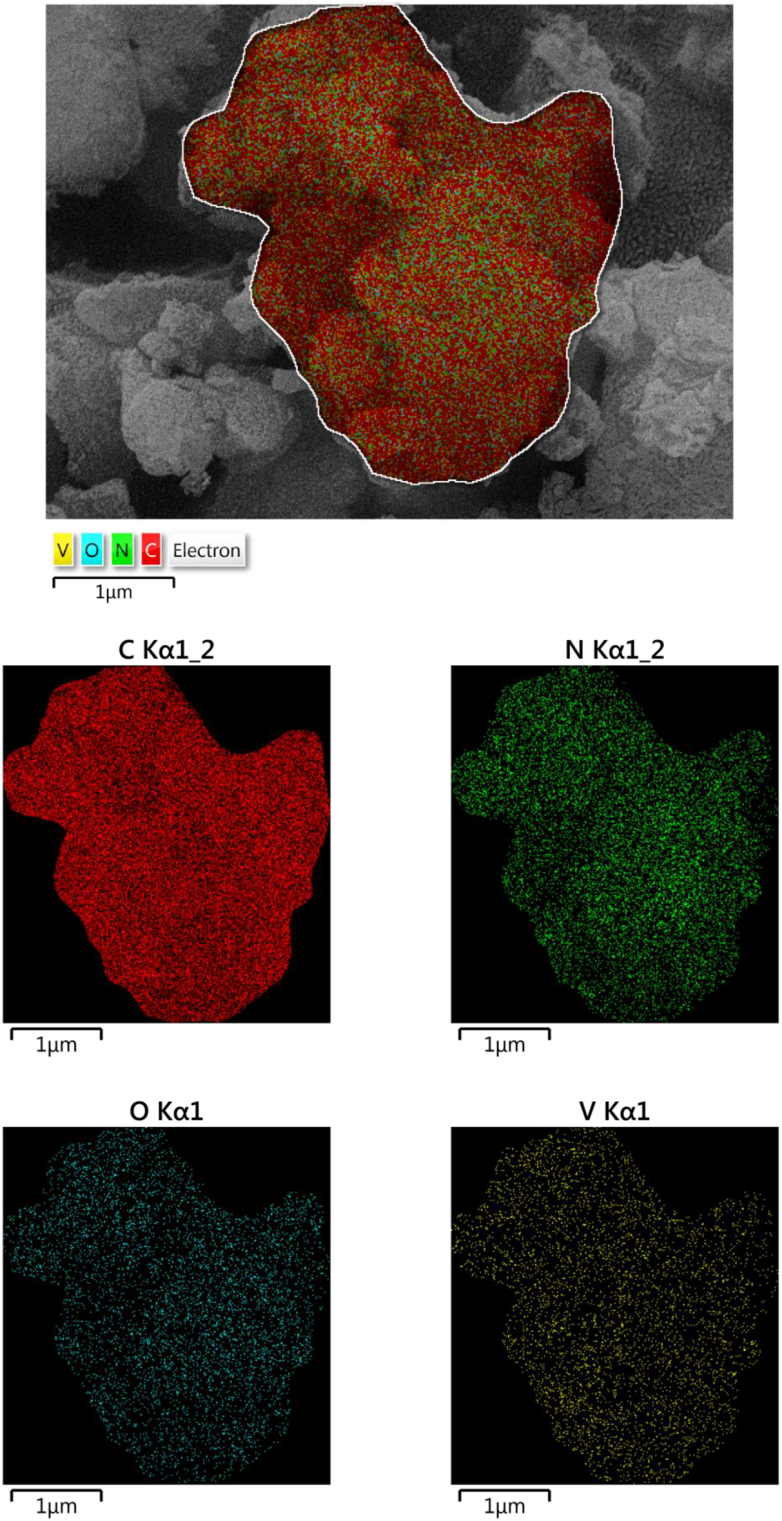
SEM equipped with EDX mapping of [(VO)TPP][(TCM)_4_].

The structure of [(VO)TPP][(TCM)_4_] and its surface morphologies was investigated by SEM equipped with EDX. SEM images and EDX mappings of [(VO)TPP][(TCM)_4_] are given in Fig. 4. From the elemental mapping technique homogeneously dispersed V = O on the structure of [(VO)TPP][(TCM)_4_] were observed. Since the EDX mapping was scanned from the surface, the composition of elements is in close agreement with the surface composition of the structure.

The results attained from CHN elemental analysis (C = 64.25%, H = 2.71%, N = 26.21%) confirmed the structure of [(VO)TPP][(TCM)_4_] (Table 1). Also, we effectively determined the amount of V (1.14 wt.%) by using ICP-OES analysis.

**Table 1:** The results of CHN elemental analysis for [(VO)TPP][(TCM)_4_], **A**, **B,** and **C**.

Sample	Type of test	C%	H%	N%
[(VO)TPP][(TCM)4]	Experimental	64.25	2.71	26.21
	Computational	66.44	3.25	22.60
Compound A	Experimental	76.51	3.87	17.63
	Computational	77.65	4.24	18.11
Compound B	Experimental	70.11	3.04	16.21
	Computational	70.28	3.54	16.39
Compound C	Experimental	58.10	2.98	13.15
	Computational	57.92	3.40	13.51

**Figure 5:**
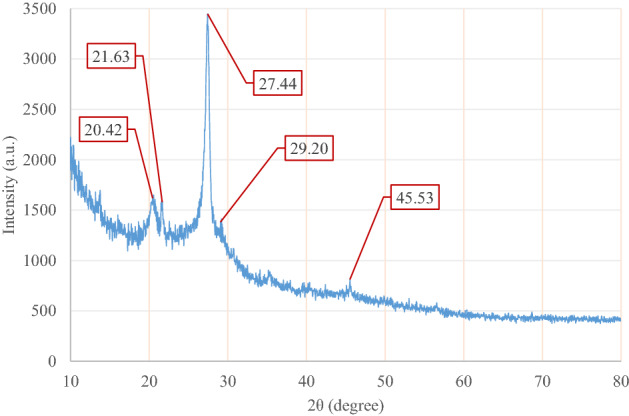
XRD pattern of [(VO)TPP][(TCM)_4_].

The XRD was used to approve the shape, size, and morphology of [(VO)TPP][(TCM)_4_]. The X-ray diffraction pattern of the [(VO)TPP][(TCM)_4_] is revealed in Fig. 5. The XRD pattern of [(VO)TPP][(TCM)_4_] at 2θ range of 20–30 has four peaks and one obvious peak located at 45.53°. The synthesized structure diameter is recognized by using Debye–Scherrer’s equation: [D = *Kλ*/(*β* cos *θ*)], which was resultant in full-width at half-maximum of the most intense peak located at 27.44°. The peak at 2θ of 27.44° is observed, which is the main feature of a physical mixture of porphyrin and V_2_O_5_. The XRD data for [(VO)TPP][(TCM)_4_] is shortened in Table 1S. This compound does not have a crystalline structure.

**Figure 6:**
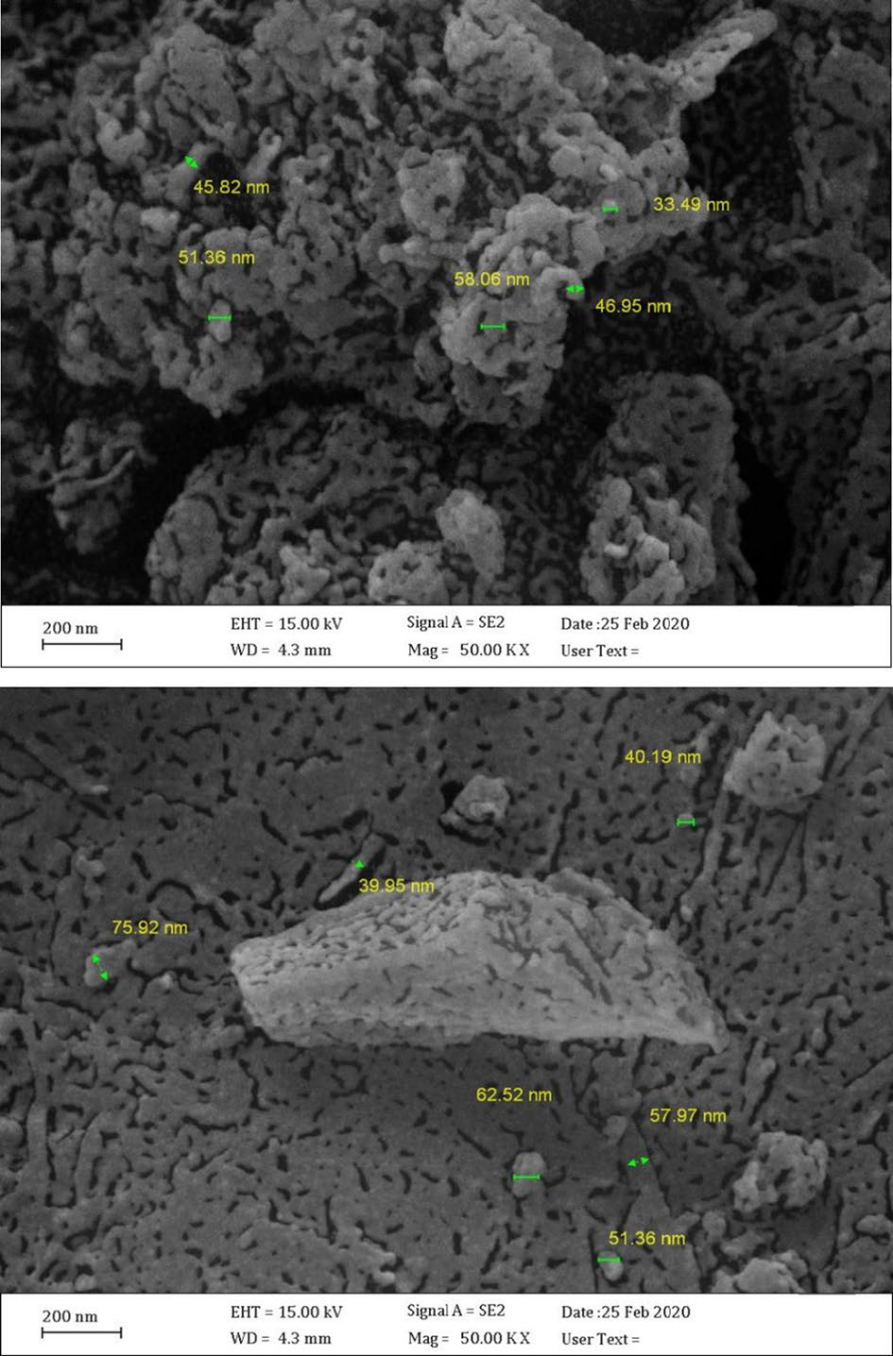
SEM images of [(VO)TPP][(TCM)_4_].

**Figure 7:**
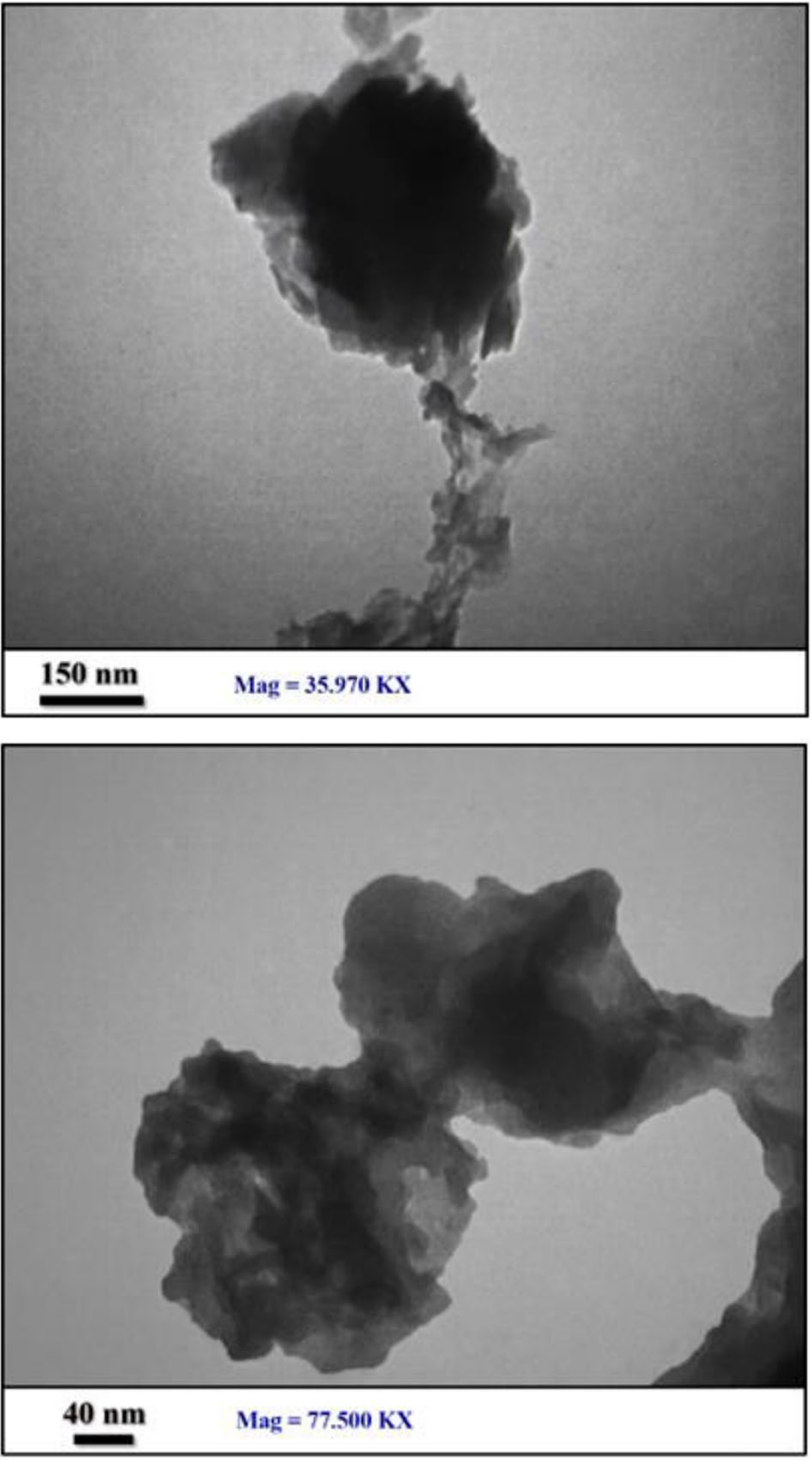
TEM images of [(VO)TPP][(TCM)_4_].

Because of the opinion that the crystallite size and morphology of these compounds can affect the catalytic activity in catalytic reactions, the morphology and crystallite size of [(VO)TPP][(TCM)_4_] were studied by using SEM and TEM techniques, and are displayed in Figs. 6 and 7, respectively. The SEM images of [(VO)TPP][(TCM)_4_] show multidimensional morphology (Fig. 6). Furthermore, the surface of the quasi-spherical aggregates of [(VO)TPP][(TCM)_4_] is covered with holes.

TEM images of [(VO)TPP][(TCM)_4_] approve the synthesis of its structure and that this complex is uneven-sized particles and most of the particles have a quasi-spherical shape (Fig. 7).

### Catalytic application of [(VO)TPP][(TCM)_4_] for the synthesis of pyrimidine-6-carbonitrile, pyrimidin-5-one, and tetrahydroquinoline-3-carbonitrile compounds

The catalytic activity of the synthesized [(VO)TPP][(TCM)_4_] as a catalyst was assessed in the typical reaction of 3,4-dimethoxybenzaldehyde, 3-amino-1,2,4-triazole, and malononitrile (Table 2). As displayed in Table 2, the desired product **8'a** was achieved with 85% yield in the presence of [(VO)TPP][(TCM)_4_] under solvent-free conditions for 10 min (Table 2, entry 6). To further progress the yield, numerous reaction factors such as the solvents, amount of catalyst, and temperature were assessed. Numerous solvents, containing ethyl acetate, *n*-hexane, acetonitrile, ethanol, water, and solvent-free condition were studied in the typical reaction. When aprotic solvents such as acetonitrile, ethyl acetate, and *n*-hexane were employed, desired product **8'a** was achieved in low yield (Table 2, entries 1 to 3). The yield of desired product **8'a** was enhanced when protic solvents such as ethanol, and water were used (Table 2, entries 4 and 5). We were pleased to find that the solvent-free condition was optimal and the reaction was completed within 10 min to provide an 85% yield of desired product **8'a** (Table 2, entry 6). The results (in terms of isolated yield and reaction time) show that solvent-free conditions are the best conditions for performing the model reaction. Control experiments approved that, in the absence of any catalyst, the reaction was not progressed (Table 2, entry 7), demonstrating that the [(VO)TPP][(TCM)_4_] as a catalyst was playing the main role in the reaction.

**Table 2:** Optimization of the reaction conditions in the typical reaction.^a^


Entry	Solvent	Catalyst loading (g)	Temperature (°C)	Time (min)	Yield (%)^b^
1	CH3CN	0.01	Reflux	60	51
2	EtOAc	0.01	Reflux	90	Trace
3	n-hexane	0.01	Reflux	120	−
4	C2H5OH	0.01	Reflux	45	70
5	H2O	0.01	Reflux	60	66
6	−	0.01	100	10	85
7	−	−	100	60	−
8	−	0.01	25	90	−
9	−	0.01	50	60	65
10	−	0.01	75	30	76
11	−	0.005	100	60	72
12	−	0.02	100	15	85
13	−	0.03	100	20	85
14^c^	−	0.01	100	60	35
15^d^	−	0.01	100	60	54
16^e^	−	0.01	100	30	61
17^f^	−	0.01	100	10	85
18^g^	−	0.01	100	30	83
19^h^	−	0.01	100	15	80

^a^Reaction conditions: 3,4-dimethoxybenzaldehyde (1 mmol; 0.166 g), 3-amino-1,2,4-triazole (1 mmol; 0.084 g, 0.014 mL), malononitrile (1 mmol; 0.066 g); ^b^Isolated yield; ^c^Compound **A** (in Fig. 3) was used as a catalyst; ^d^Compound **B** (in Fig. 3) was used as a catalyst; ^e^Compound **C** (in Fig. 3) was used as a catalyst; ^f^{[VO(TPPA)][C(CN)_3_]_4_}^72^ was used as a catalyst; ^g^Chitosan based vanadium oxo (ChVO)^75^ was used as a catalyst; ^h^[VO(TPPABr)]CBr_3_^76^ was used as a catalyst.

A study of the temperature was also carried out. The desired product **8'a** was furnished in 85% yield at 100 °C. Shrinking the temperature to room temperature did affect the reaction effectiveness and the desired product was attained in low yield (Table 2, entries 8 to 10), while a higher temperature of 120 °C was not useful for the yield either. Additional investigation of the amount of the [(VO)TPP][(TCM)_4_] catalyst showed that 0.01 g (equal to 9.54 mmol) was the best loading for the reaction. Decreasing the amount of catalyst to 0.005 g led to a lower yield (Table 2, entry 11). A further increase in the amount of catalyst to 0.02 g and 0.03 g did not affect the yield (Table 2, entries 12 and 13).

Moreover, as additional evidence of the practical applicability of this approach, the typical reaction was scaled up to 10 mmol. The result showed that the reaction progressed effectively and the chosen product **8'a** could be achieved without shrinking the reaction efficacy. Also, as you can see in Table 2 (entries 17–19), in the same conditions, by changing the catalyst, there was an insignificant difference in the isolated yield and reaction time.

To determine the substrate scope of this catalytic approach, as demonstrated in Table 3, employing aldehydes including electron-neutral, electron-poor, and electron-rich groups gave the target products **8**, **8'**, **9**, and** 10** in suitable yields and in moderately good reaction times without synthesis of any organic side products. Using aldehyde possessing electron-withdrawing, and electron-releasing groups almost the same results were attained in several reaction parameters such as isolated yields and reaction times. Agreeably, heteroaryl aldehydes, such as 1*H*-indole-3-carbaldehyde, naphthalene-1-carbaldehyde, and naphthalene-2-carbaldehyde were also appropriate for this reaction, synthesizing the related products in good yields.

**Table 3:**
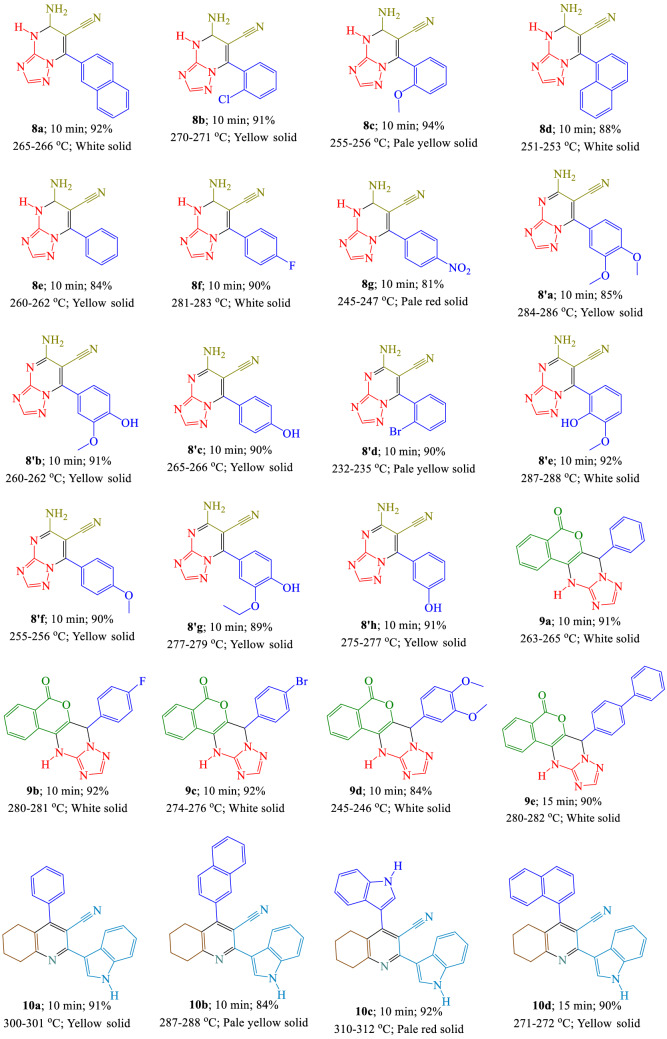
Synthesis of target products **8**, **8'**, **9**, and **10** by using [(VO)TPP][(TCM)_4_] (0.01 g is equal to 9.54 mmol) at 100 °C under solvent-free conditions.^a,b^. ^a^Reaction conditions: Aldehyde (1 mmol), 3-amino-1,2,4-triazole (1 mmol; 0.084 g, 0.014 mL), malononitrile (1 mmol; 0.066 g) [for the synthesis of **8** and **8'**]; Aldehyde (1 mmol), 3-amino-1,2,4-triazole (1 mmol; 0.084 g, 0.014 mL), 4-hydroxycoumarin (1 mmol; 0.162 g) [for the synthesis of **9**]; Aldehyde (1 mmol), cyclohexanone (1 mmol; 0.098 g, 0.104 mL), ammonium acetate (1 mmol; 0.077 g), 3-(1*H*-indol-3-yl)-3-oxopropanenitrile (1 mmol; 0.184 g) [for the synthesis of **10**];^b^ Isolated yield.

**Figure 8:**
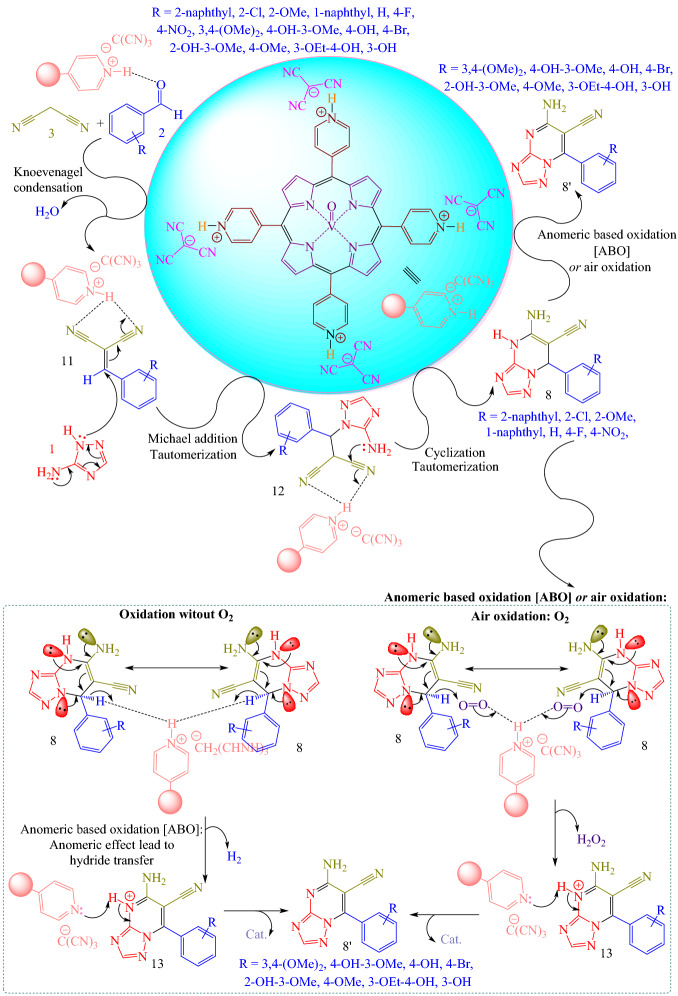
Suggested reaction mechanism for the synthesis of 5-amino-7-(aryl)-4,7-dihydro-[1,2,4]triazolo[1,5-*a*]pyrimidine-6-carbonitriles **8** and 5-amino-7-(aryl)-[1,2,4]triazolo[1,5-*a*]pyrimidine-6-carbonitriles **8'**.

The recyclability and reusability of the catalytic medium were studied in the typical reaction under the optimized reaction conditions. After the end of the reaction, the reaction mixture was cooled to room temperature, then, diluted in hot ethanol and the catalyst was separated by filtration, and washed with ethyl acetate and water, dried under vacuum. In the next step, the crude desired product **8'a** was evaporated under reduced pressure and recrystallized by hot ethanol to attain the pure desired product **8'a**. The recycled catalyst was reused in the typical reaction to the next round. The catalytic medium was established to be effective for up to six successive runs without any noteworthy loss in its catalytic activity and stability (Table 4). Refer to the supplementary information section to study the UV–Vis, EDX, SEM equipped with EDX mapping, and SEM of recycled catalyst (Fig. 8S).

**Table 4:** Recyclability and reusability studies of [(VO)TPP][(TCM)_4_] in the typical reaction.^a^


Experimental round	Fresh	1	2	3	4	5	6
Yield (%)^b^	85	84	82	79	76	74	70

^a^Reaction conditions: 3,4-dimethoxybenzaldehyde (1 mmol; 0.166 g), 3-amino-1,2,4-triazole (1 mmol; 0.084 g, 0.014 mL), malononitrile (1 mmol; 0.066 g); ^b^Isolated yield.

**Figure 9:**
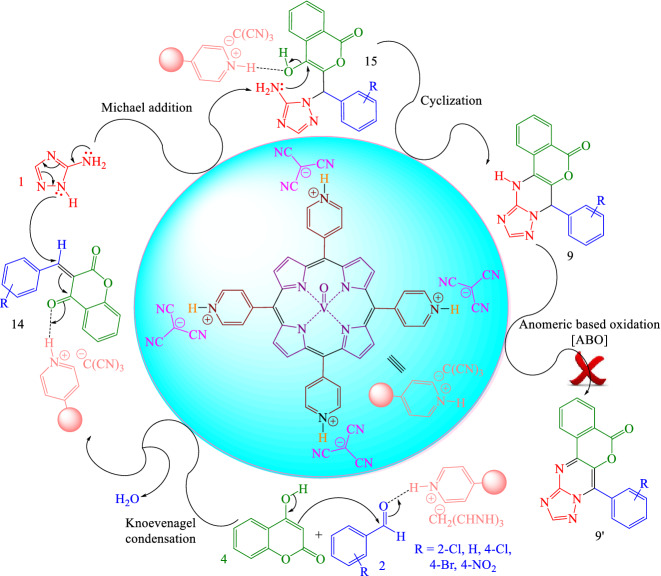
Proposed reaction mechanism for the synthesis of 7-(aryl)-7,12-dihydro-5*H*-isochromeno[4,3-*d*][1,2,4]triazolo[1,5-*a*]pyrimidin-5-ones **9**.

**Figure 10:**
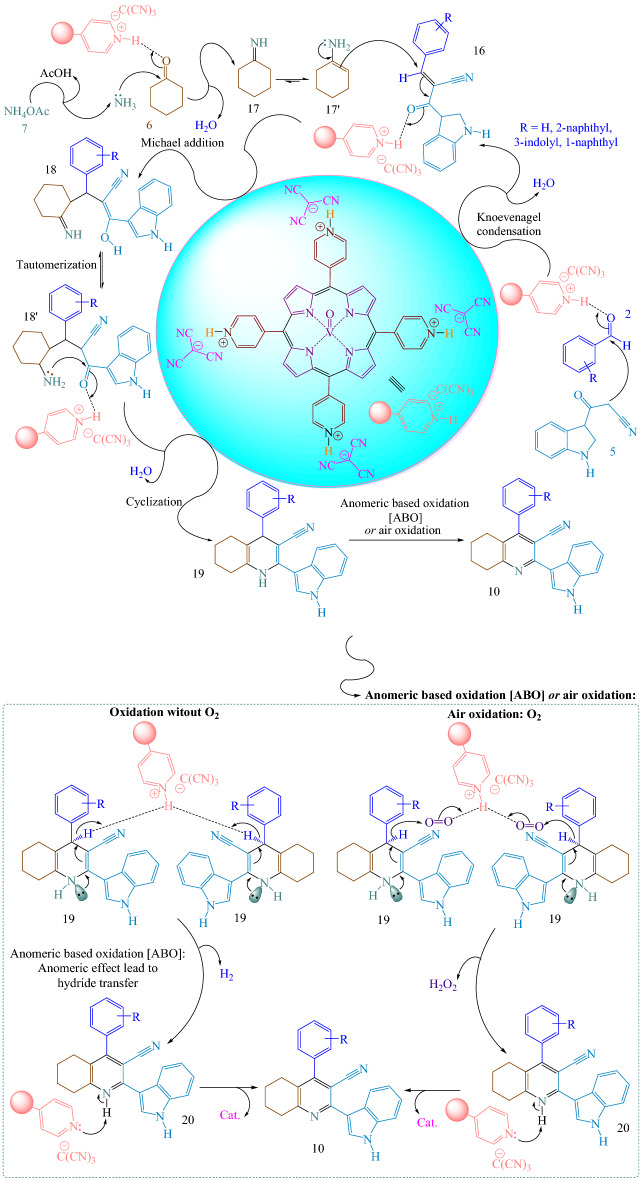
Probable reaction mechanism for the synthesis of 4-(aryl)-2-(1*H*-indol-3-yl)-5,6,7,8-tetrahydroquinoline-3-carbonitriles **10**.

Proposed reaction mechanism for the synthesis of 5-amino-7-(aryl)-4,7-dihydro-[1,2,4]triazolo[1,5-*a*]pyrimidine-6-carbonitriles **8** and 5-amino-7-(aryl)-[1,2,4]triazolo[1,5-*a*]pyrimidine-6-carbonitriles **8'** was displayed in Fig. 8^18^. Initially, [(VO)TPP][(TCM)_4_] activates the carbonyl group of the aldehyde **2**. At that time, the Knoevenagel condensation reaction of malononitrile **3** with activated aldehyde **2** happened to afford intermediate **11** with the removal of one molecule of water. Then, nucleophilic attack of 3-amino-1,2,4-triazole **1** to intermediate **11** gives intermediate **12** via Michael addition. Then, intramolecular cyclization between the amine group and the cyanide functional group of intermediate **12** to synthesize product **8.** To finish, product **8** via a cooperative vinylogous anomeric-based oxidation (ABO) was converted to desired product **8'**. We suggest that this step might have proceeded via unusual hydride transfer as well as the Cannizzaro reaction (Fig. 9S)^77^ and H_2_ release from tricyclic orthoamide (Fig. 10S)^78–80^. We have proposed anomeric-based oxidation (ABO) for the last step in the mechanistic approach to the synthesis of some organic compounds (Figs. 11S–18S)^81–90^. To confirm the aforementioned suggestion, a reaction occurred under both nitrogen, and argon atmosphere and in the absence of any molecular oxygen. It was established that the reaction progressed under the atmosphere of nitrogen and argon slightly slower than the normal reaction condition (air atmosphere). By investigating the aforesaid evidence, the conversion of product **8** to **8'** might be occurred by unusual hydride transfer and release of molecular hydrogen (H_2_) in the presence of [(VO)TPP][(TCM)_4_]. The C–H bond is weakened via anomeric supporting of the nitrogen lone pairs into the vacant anti-bonding of C–H (^*^_C–H_ orbital)^1,2,91,92^ which can be reacted with a proton to furnish molecular hydrogen.

Probable reaction mechanism for the synthesis of 7-(aryl)-7,12-dihydro-5*H*-isochromeno[4,3-*d*][1,2,4]triazolo[1,5-*a*]pyrimidin-5-ones **9** was exhibited in Fig. 9^25^. At first, [(VO)TPP][(TCM)_4_] activates the carbonyl group of the aldehyde **2**. At that time, the Knoevenagel condensation reaction of 4-hydroxycoumarin **4** with activated aldehyde **2** occurred to provide intermediate **14** with the elimination of one molecule of water. In the next step, nucleophilic attack of 3-amino-1,2,4-triazole **1** to intermediate **14** affords intermediate **15** via Michael addition and after tautomerization. Finally, intramolecular cyclization between the amine group and the hydroxyl group of intermediate **15** to furnish product **9**.

Suggested reaction mechanism for the synthesis of 4-(aryl)-2-(1*H*-indol-3-yl)-5,6,7,8-tetrahydroquinoline-3-carbonitriles **10** was showed in Fig. 10^30^. At the start, [(VO)TPP][(TCM)_4_] activates the carbonyl group of the aldehyde **2**. Then, the Knoevenagel condensation reaction of 3-(1*H*-indol-3-yl)-3-oxopropanenitrile **5** with activated aldehyde **2** occurred to provide intermediate **16** with the removal of one molecule of water. On the other hand, [(VO)TPP][(TCM)_4_] activates the carbonyl group of cyclohexanone **6**. At that moment, the nucleophilic attack of ammonia (in situ generated from ammonium acetate **7**) to activated cyclohexanone **6** happened to afford intermediate **17** with the elimination of one molecule of water which tautomerized to intermediate **17'**. In the next step, nucleophilic attack of intermediate **17'** to intermediate **16** via Michael addition furnishes intermediate **18** which is tautomerized to intermediate **18'**. Then, intramolecular cyclization between the amine and carbonyl group of intermediate **18'** to synthesize intermediate **19**. Lastly, intermediate **19** via a cooperative vinylogous anomeric based oxidation (ABO) was converted to desired product **10**. We suggest that the last step might have progressed via anomeric-based oxidation (ABO) according to the aforementioned facts (Fig. 8).

Also, in another study, to investigation the activity and efficacy of the system, [(VO)TPP][(TCM)_4_] was compared with the catalysts reported in previous works (Table 5). As you can see in Table 5, in the similar reaction conditions, there was an unimportant difference in the reaction time and isolated yield.

**Table 5:** Comparison of [(VO)TPP][(TCM)_4_] with other reported catalysts.

Reaction condition	Time (min)	Yield (%)^a^ [References]
	a: 120	93^76^
	b: 60	91
	a: 120	90^76^
	b: 45	93
	c: 45	89^75^
	b: 10	95
	c: 55	88^75^
	b: 60	84

^a^Isolate yield.

## Conclusion

In summary, we have established a benign, versatile, and expedient oxovanadium(V)-[5,10,15,20-tetrakis(pyridinium)-porphyrinato]-tetra(tricyanomethanide) [(VO)TPP][(TCM)_4_] catalyzed synthesis of numerous pyrimidine-6-carbonitrile, pyrimidin-5-one, and tetrahydroquinoline-3-carbonitrile compounds via a cooperative geminal-vinylogous anomeric based oxidation under solvent-free conditions at 100 °C. The reaction tolerates various functional groups and affords a mild and efficient approach for the synthesis of useful triazolopyrimidines and tetrahydroquinolines. Mainly, the catalytic system could be effortlessly recycled and reused for six consecutive runs without substantial loss of its activity, advising great potential for industrial uses. The key highlights of this approach are ease in the catalyst synthesis, being environmentally friendly, good yields, short reaction times, and prevention of toxic solvents. Additional scope and mechanistic investigations of the reaction are occurring in our laboratory.

## Experimental

### General information

^1^H (400 MHz) and ^13^C NMR (101 MHz) spectra were recorded on DRX‐400 MHz instrument. All signals are expressed as ppm (*δ*) and are referenced to the non-deuterated solvent peak DMSO-*d*_*6*_. Coupling constants (*J*) are given in Hz and refer to apparent peak multiplicities. The subsequent abbreviations are employed: *s* = singlet, *d* = doublet, *t* = triplet, *q* = quartet, *m* = multiplet. Melting points were determined with a Thermo Scientific apparatus and are uncorrected. Sigma-Aldrich Silica gel (high-purity grade, pore size 60 Å, 230–400 mesh particle size, 40–63 mm particle size) was used for flash column chromatography. Analytical thin-layer chromatography (TLC) was performed by using commercial silica-gel plates (Merck 60 F254), and spots were observed with UV light (254 nm). All reagents were employed as received from commercial sources unless identified otherwise, or prepared as described in the literature. The catalyst was characterized for its gold content by ICP-AES (using Agilent 7700 × apparatus). UV–vis spectra of the silver-gold solution were performed at ambient temperature using a Perkin‐Elmer spectrophotometer by scanning wavelengths between 200 and 800 nm. Transmission electron micrographs were taken of the sample using a Zeiss‐EM10C electronic microscope operating at an accelerating voltage of 100 kV. The morphologies of the catalyst were detected by SEM with a ZEISS SIGMA VP‐500 scanning microscope operated equipped with an X‐ray detector Oxford Instrument for microanalysis (SEM‐EDX) presenting a 133 eV resolution at 20 kV. A small portion of each sample powder was coated on a metallic disk holder and covered with a thin gold layer before the SEM analysis. XRD measurements were prepared using an X' Pert Pro apparatus with Cu_*Kα*_ (λ = 0.154 nm) radiation. The angular 2θ diffraction range was between 10° and 80°. The data were collected with an angular step of 0.05° at 3 s per step and sample rotation. Cu_*Kα*_ radiation was attained from a copper X-ray tube operated at 40 kV and 30 mA. FE-SEM images were performed by a Zigma. TGA and DTA were achieved on a Rheometric Scientific STA 1500 (heating rate of 10 °C min^−1^ up to 600 °C; under a nitrogen atmosphere at 25 °C). FT-IR spectra were analyzed by Perkin‐Elmer FT-IR-17259 instrument by KBr disks. ICP‐OES was made on an Agilent 7700 × apparatus. CHN elemental analyses were carried out by using the Thermo Finnigan apparatus (FlashEA 1112 series). Diffuse reflectance UV–Vis spectroscopy (DRUVS) was used by a V-670, JASCO spectrophotometer to determine band gaps over the wavelength range of 200–800 nm. High-resolution mass spectra were attained on a Finnigan MAT 95 XPAPI-GC-Trap tandem Mass spectrometer system.

### General procedure for the synthesis of [(VO)TPP][(TCM)_4_]

#### Initial step

A mixture of 10 mL (0.15 mol) of pyrrole, 13.5 mL (0.15 mol) of pyridine-4-carbaldehyde, and 100 mL of a boiling propionic acid was refluxed for 3 h and cooled down. Then, propionic acid was distilled off in a vacuum for 60 min, and 60 mL of methanol and 5 mL of concentrated ammonia were added to the residue. The precipitate was filtered off, washed with methanol, and dried for 60 min. The reaction product was extracted in a rotary evaporation apparatus with chloroform, and the attained solution was subjected to chromatography on alumina. The reaction product was precipitated with ethanol, filtered off, washed with methanol, and dried at 60 °C in a vacuum for 60 min. The residue was dissolved in 150 mL of 3% HCl, and 5 mL of concentrated ammonia and the precipitate was filtered off, washed with water, and dried at 60 °C in a vacuum for 60 min^40^; isolated yield of **A**: 5.89 g (27%).

#### Second step

A mixture of 5 g of first-step product **A** and 1.2 g of V_2_O_5_ was refluxed for 3 h in 100 mL of chloroform. Then, the reaction mixture was cooled down to room temperature and poured into water. The precipitate was filtered off, dried in a vacuum at 60 °C for 60 min, and purified by column chromatography on alumina. The eluate was evaporated to dryness; isolate yield of **B**: 4.16 g (67%).

#### Third step

A solution of 4 g of second-step product **B** and 50 mL of HCl (1 N) was stirred for 60 min at room temperature. Then, the reaction mixture was diluted and the precipitate was filtered off, washed with diethyl ether, and dried in a vacuum at 60 °C for 60 min; isolate yield of **C**: 3.04 g (76%).

#### Final step

Potassium tricyanomethanide was synthesized according to the reported procedure^93–99^. Potassium tricyanomethanide (4 mmol; 0.52 g) dissolved in 50 mL of deionized water. Then 2 g of third-step product **C** was added to the revealed mixture and it was stirred for 24 h at room temperature. Then, the reaction mixture was diluted and the precipitate was filtered off and washed three times with diethyl ether. The obtained powder dried at 100 °C for 3 h (Fig. 3); isolate yield of **[(VO)TPP][(TCM)**_**4**_**]**: 2.07 g (82%).

### General procedure for the synthesis of compounds 8, 8', and 9 by using [(VO)TPP][(TCM)_4_]

A mixture of aldehyde (1 mmol), 3-amino-1,2,4-triazole (1 mmol; 0.084 g, 0.014 mL), malononitrile (1 mmol; 0.066 g) [for the synthesis of **8** and **8'**] or 4-hydroxycoumarin (1 mmol; 0.162 g) [for the synthesis of 9], and [(VO)TPP][(TCM)_4_] (0.01 g is equal to 9.54 mmol) was stirred and heated at 100 °C under solvent-free conditions for appropriate times (Table 3). After completion of the reaction as monitored by TLC (by using ethyl acetate *versus n*-hexane; 3:7), the mixture was diluted in hot ethanol, the catalyst was separated by filtration, and the solvent was removed under vacuum. Pure products **8**, **8'**, and **9** were achieved after recrystallization from ethanol. The [(VO)TPP][(TCM)_4_] catalyst was washed with ethyl acetate and water, dried at 100 °C for 60 min, and reused in six sequential runs (Table 4).

### General procedure for the synthesis of compound 10 by using [(VO)TPP][(TCM)_4_]

A mixture of aldehyde (1 mmol), cyclohexanone (1 mmol; 0.098 g, 0.104 mL), ammonium acetate (1 mmol; 0.077 g), 3-(1*H*-indol-3-yl)-3-oxopropanenitrile (1 mmol; 0.184 g), and [(VO)TPP][(TCM)_4_] (0.01 g is equal to 9.54 mmol) was stirred and heated at 100 °C under solvent-free conditions for appropriate times (Table 3). After the end of the reaction as observed by TLC (by using ethyl acetate *versus n*-hexane; 3:7), the mixture was diluted in hot ethanol, the catalyst was separated by filtration, and the solvent was removed under vacuum. Pure product **10** was attained after recrystallization from ethanol.

## Supplementary Information

Supplementary Information.

## Data Availability

The datasets generated during and/or analysed during the current study are available from the corresponding author on reasonable request.
