# Supplementary material for: Synthesis of pyrimidine-6-carbonitriles, pyrimidin-5-ones, and tetrahydroquinoline-3-carbonitriles by new superb oxovanadium(V)-[5,10,15,20-tetrakis(pyridinium)-porphyrinato]-tetra(tricyanomethanide) catalyst via anomeric based oxidation
Source: Sci Rep. 2022 Nov 14;12:19537. doi: 10.1038/s41598-022-23956-6 (PMC9663709; doi:10.1038/s41598-022-23956-6)
Supplement: Supplementary file 1 — Supplementary Information. [file 41598_2022_23956_MOESM1_ESM.docx]

**Supplementary Information**

**Synthesis of pyrimidine-6-carbonitriles, pyrimidin-5-ones, and tetrahydroquinoline-3-carbonitriles by new superb Oxovanadium(V)-[5,10,15,20-tetrakis(pyridinium)**-**porphyrinato]-tetra(tricyanomethanide) catalyst *via* anomeric based oxidation**

Mohammad Dashteh,^b^ Sajjad Makhdoomi,^a,b^ Saeed Baghery,^b^* Mohammad Ali Zolfigol,^b^* Ardeshir Khazaei,^b^* Yanlong Gu^c^

^a^ Department of Organic Chemistry, Faculty of Chemistry, Bu-Ali Sina University, Hamedan 6517838683, Iran.

^b^ Department of Pharmacology and Toxicology, School of Pharmacy, Hamedan University of Medicinal Science, Hamedan, Iran.

^c^ School of Chemistry and Chemical Engineering, Huazhong University of Science and Technology, 1037 Luoyu road, Hongshan District, Wuhan 430074.

Fax: +988133493009; E-mail: zolfi@basu.ac.ir and [mzolfigol@yahoo.com](mailto:mzolfigol@yahoo.com) (M.A. Zolfigol); [Khazaei_1326@yahoo.com](mailto:Khazaei_1326@yahoo.com) (A. Khazaei); saadybaghery@yahoo.com (S. Baghery).

Typically, the favored conformation of substituted saturated heterocycles should follow the main ideologies that dictate the conformational behavior of cycloalkanes. Though, the presence of non-carbon atoms in the ring makes substantial structural changes for example variations in bond lengths and angles; therefore, nonbonded interactions between substituent groups in heterocyclic compounds cannot be of a similar amount. By the way, the presence of lone pairs of electrons in heterocyclic systems can have obvious effects on the conformation of these systems (Fig. 1S) [1S-5S].

**Figure 1S.** The effect of the presence of lone electron pairs in heterocyclic systems and the direction of their conformation.

The anomeric effect is individual of the most significant factors that affect the conformational behavior and the reactivity of saturated [heterocyclic](https://www.sciencedirect.com/topics/chemistry/heterocyclic-compound) compounds. Generally, the collected experimental observations (such as spectroscopic data, structural variations, and relative reactivity) and theoretical descriptions offer that two reasons contribute to the anomeric effect: (*a*) [dipole-dipole interactions](https://www.sciencedirect.com/topics/chemistry/dipole-interaction) as a destabilizing of the equatorial [conformer](https://www.sciencedirect.com/topics/chemistry/conformational-isomer), and (*b*) a stereo-electronic interaction which stabilizes the axial orientation of the anomeric substituent (Fig. 2S) [1S-5S].

**Figure 2S.** The presence of two main factors in the anomeric effect.

FT-IR spectroscopy was employed to investigate the structure of [(VO)TPP][(TCM)_4_], and its spectrum was approved in the region 400-4000 cm^-1^ (Fig. 3S). Figure 1 displays strong peaks around 3408 to 3207 cm^-1^ owing to the stretching vibration of N−H groups. Nevertheless, again the peak achieved at 2204 cm^-1^ could be assigned because of the stretching vibration of C≡N groups which established the existence of tricyanomethanide counter ion. The peaks in the 1642 cm^-1^ could be related to the C=N bond. Also, the peaks at 990 cm^-1^ near the band at 1077 cm^-1^, which was regarded as the V=O stretching bond [6S]. The differences in the FT-IR spectra from **a** to **d** demonstrate the synthesis of [(VO)TPP][(TCM)_4_].

| 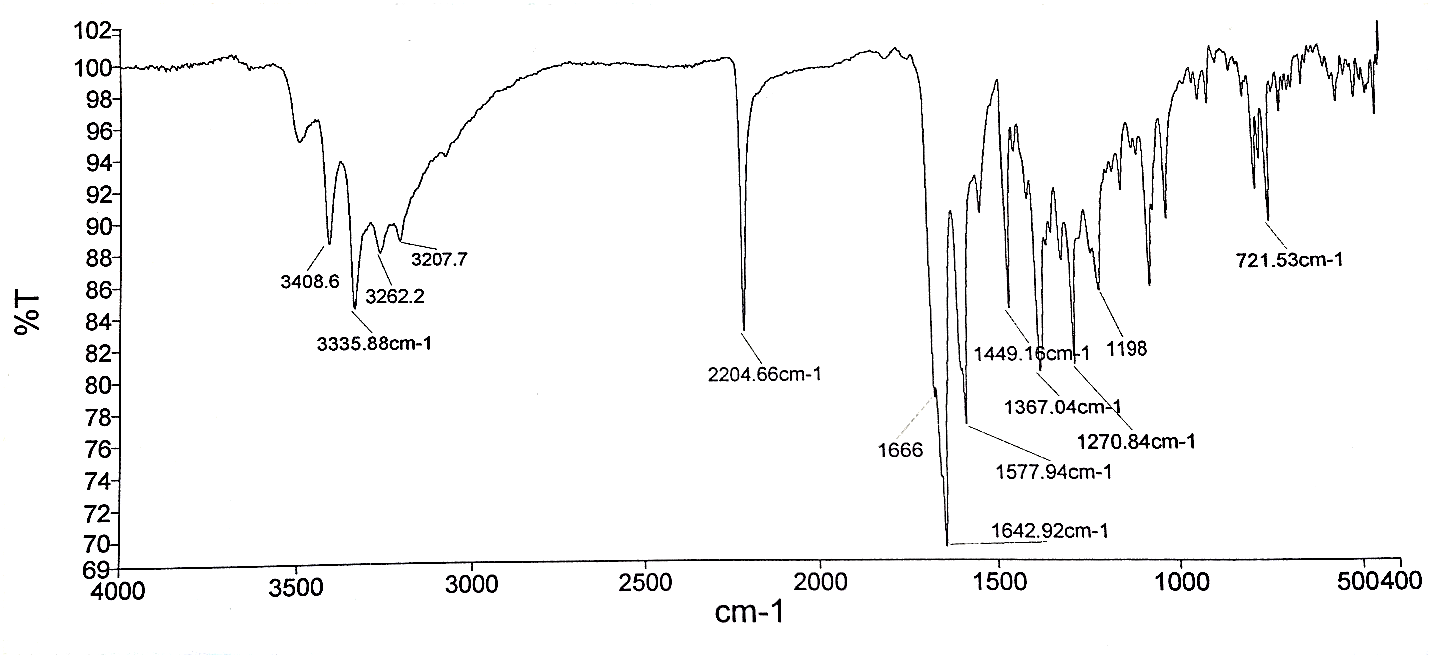  (a) |
| --- |
| 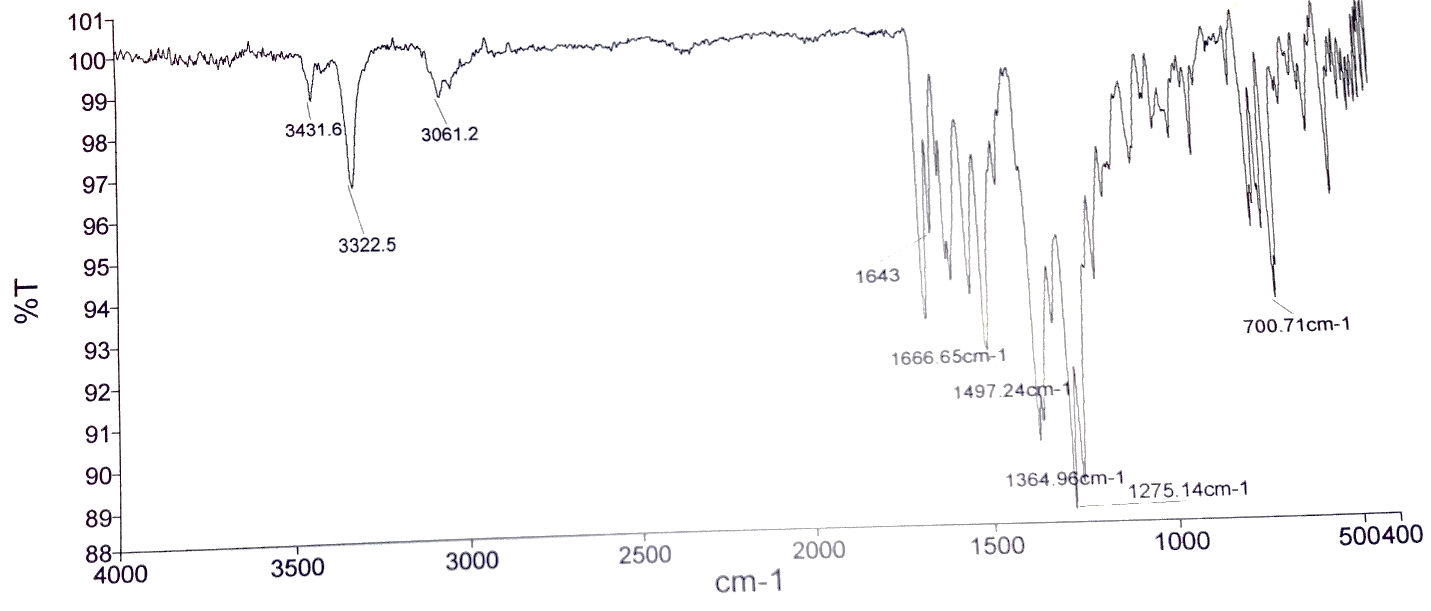  (b) |
| 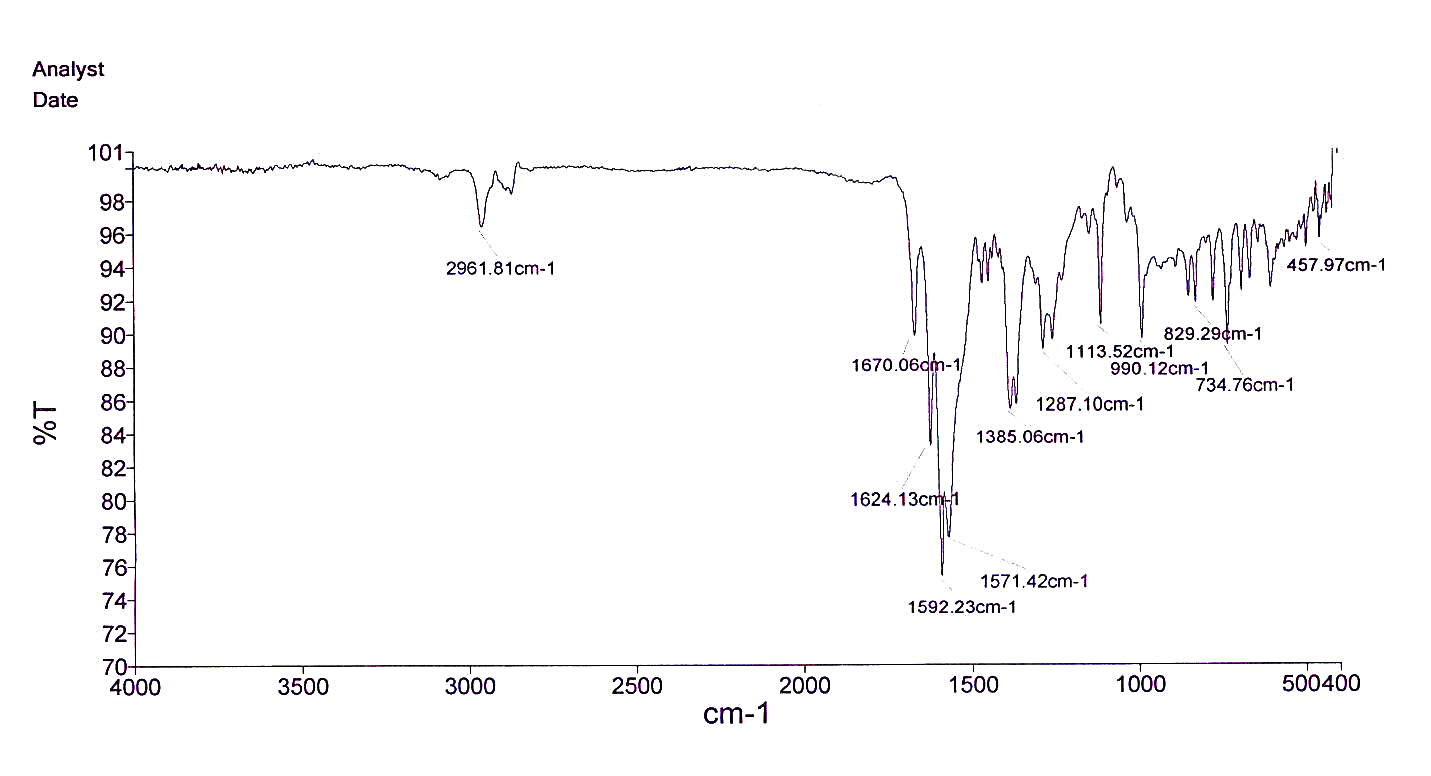  (c) |
| 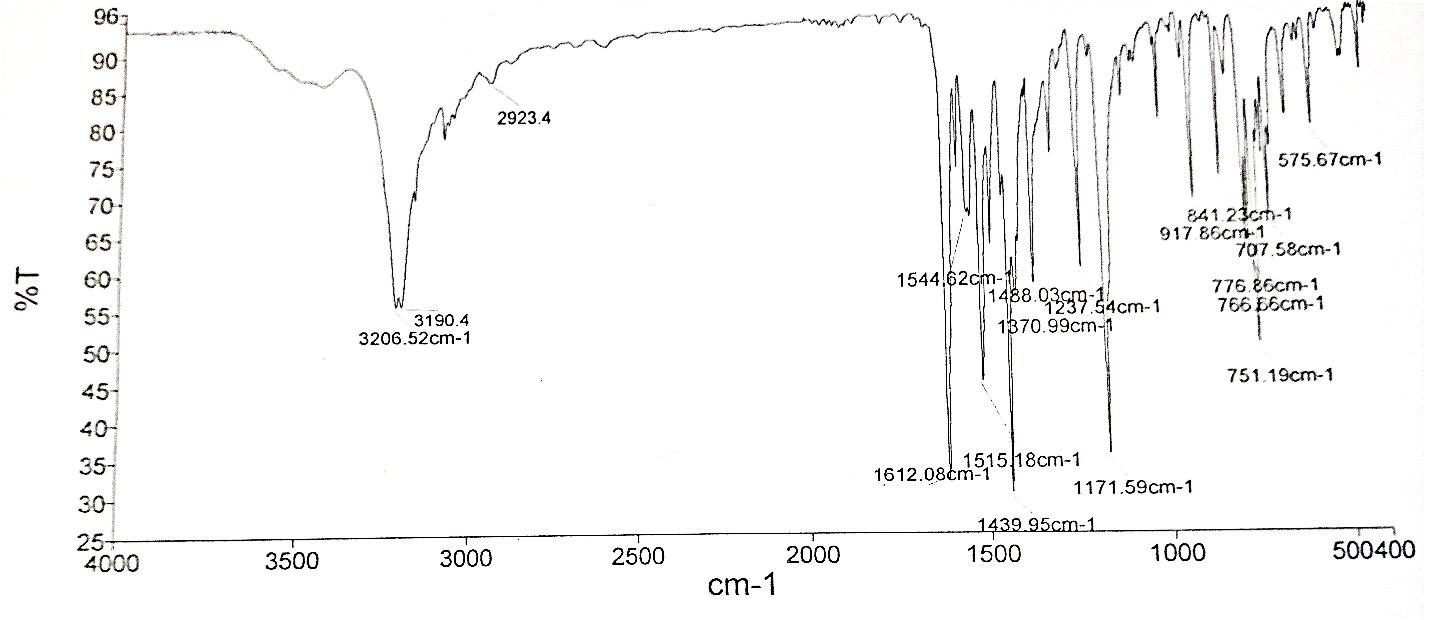  (d) |

**Figure 3S.** FT-IR spectra of [(VO)TPP][(TCM)_4_] (a), **A** (b), **B** (c) and **C** (d).

To determine the content of the elements, EDX analysis was studied, and the obtained results are shown in Fig. 4S. The EDX results of [(VO)TPP][(TCM)_4_] show the presence of C, N, O, and V. The results display that the [(VO)TPP][(TCM)_4_] has been produced. These outcomes confirmed the homogeneous dispersion of V=O on the structure of [(VO)TPP][(TCM)_4_] and indicate fewer impurities in synthesized [(VO)TPP][(TCM)_4_]*.*


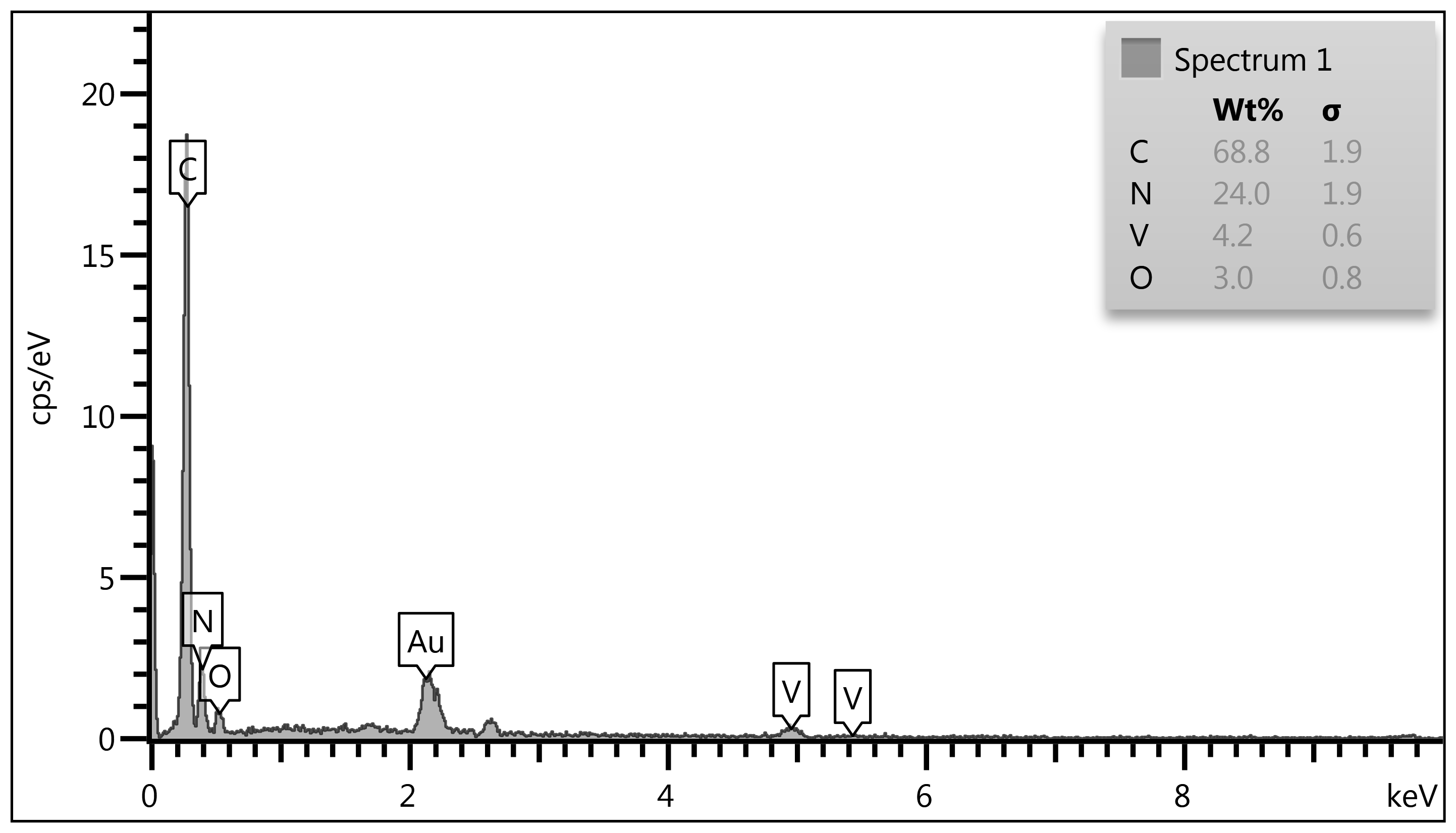


**Figure 4S.** EDX analysis of [(VO)TPP][(TCM)_4_].

The stability of the [(VO)TPP][(TCM)_4_] was determined by using TGA and DTA with a flow rate N_2_ of 10 mL per minute as displayed in Fig. 5S. Four stages of weight loss are detected. The first thermal event in the temperature range of 25 to 120 ^ο^C was associated with the dehydration of the [(VO)TPP][(TCM)_4_], with a weight loss of 5.07 %. The second thermal event happening in the temperature range between 120 and 300 ^ο^C was ascribed to the decomposition of tricyanomethanide counter ion with a weight loss of 4.08 %. The third thermal event between 300 and 390 ^ο^C was associated with the decomposition of the V=O band with a weight loss of 3.82 %. The final thermal event in the temperature range of 390 to 600 ^ο^C was related to the decomposition of organic species of [(VO)TPP][(TCM)_4_], with a weight loss of 15.47 %. Above 600 ^ο^C, no weight loss was detected.

| (a) |
| --- |
| (b) |

**Figure 5S.** TGA (a) and DTA (b) analyses of [(VO)TPP][(TCM)_4_].

The DRS spectrum of [(VO)TPP][(TCM)_4_] is displayed in Fig. 6S-a. The DRS spectrum of the vanadium [(VO)TPP][(TCM)_4_] heated at 350 ^o^C exhibits an absorption band at 323.93 nm ascribed to vanadium(V)-oxide charge transfer transition [7S]. This transition intensity shrinkage upon thermal treating, proposing that upon heating V^V^ centers are reduced to V^IV^. This result is following that reported in the literature (dehydration of V_2_O_5_.nH_2_O leads to some reduction of vanadium ion) [8S]. While, for the [(VO)TPP][(TCM)_4_] treated at 600^o^C, the intensity of the charge transfer band increased with a bathochromic shift, demonstrating the oxidation of vanadium V^IV^ sites. In other words, we can say that the absorption band in the wavelength range of 230−600 nm results from the electron transfer from O_2p_→V_3d_, and the absorption band in the wavelength range of 270−360 nm results from electron transfer from octahedron coordinate vanadium. But the absorption band in the wavelength range of 200−270 nm is from completely isolated octahedron vanadium [9S].

The band gap energy (*E_g_*) of the V_2_O_5_ powders can be assessed by the Tauc’s plot [*αhυ = A(hυ − E_g_)^n/2^*], where *α*, *h*, *υ*, *A,* and *E*_g_ are an indication of the absorption coefficient, Planck’s constant, light frequency, constant value, and the band gap energy, respectively, and *n* depends on the characteristics of the transitions in a semiconductor (indirect transition *n* = 1 and direct transition *n* = 4). The Kubelka–Munk function (derived from DRS) of [(VO)TPP][(TCM)_4_] is shown in Figure 6S-b, and its band gap is determined to be 3.87 eV. The attained value is in good agreement with the data reported in the literature [10S].

| (a) |
| --- |
| (b) |

**Figure 6S.** DRS spectrum (a) and Kubelka–Munk function plot (b) of [(VO)TPP][(TCM)_4_].

The UV–vis absorption spectrum of [(VO)TPP][(TCM)_4_] solutions was displayed in Fig. 7S. From Fig. 7S, it can be seen that the [(VO)TPP][(TCM)_4_] solution shows one characteristic band at 334 nm, which is because of the V=O charge-transfer absorption peak in the porphyrin cage. The [(VO)TPP][(TCM)_4_] solution appearances, one clear absorption peak at 563 nm (B-band) ascribed to the deeper *π*-levels-LUMO transition, and at 621 nm (Q-band) related to the characteristic absorption of porphyrin base [11S].

**Figure 7S.** UV–Vis absorption spectrum of [(VO)TPP][(TCM)_4_].

The UV-Vis and EDX spectra, SEM equipped with EDX mappings, SEM image, and ICP-OES analysis of [(VO)TPP][(TCM)_4_] after six consecutive runs approve the stability of its structure in the course of the recycling process (Fig. 8S). The structure of recycled catalyst was approved by a UV-visible investigation (Figure 8S-a) that displays absorption bands at 331 nm related to V=O charge-transfer absorption peak in the porphyrin cage, at 564 nm (B-band) attributed to the deeper *π*-levels-LUMO transition, and at 623 nm (Q-band) linked to the characteristic absorption of porphyrin base. The elemental composition of the recycled catalyst was determined by the EDX spectrum. As can be seen in Fig. 8S-b, C, O, N, and V species have been detected in the recycled catalyst. EDX mapping was scanned from the surface of the recycled catalyst, and the composition of elements was approved with the surface composition of the structure (Fig. 8S-c). SEM micrograph of the recycled catalyst (Fig. 8S-d) showed that no clear conglutinated particles were detected and the surface seemed to be covered with scale particles. The catalyst showed a regular shape and uniform distribution of particle size. The content of the vanadium in the recycled catalyst was found to be 0.95 wt% as determined by ICP-OES analysis. The detected decrease in catalytic activity perhaps originates from the V=O separation from the total structure of the catalyst and the deformation of the structure of [(VO)TPP][(TCM)_4_] during the catalytic method.

| (a) |
| --- |
| 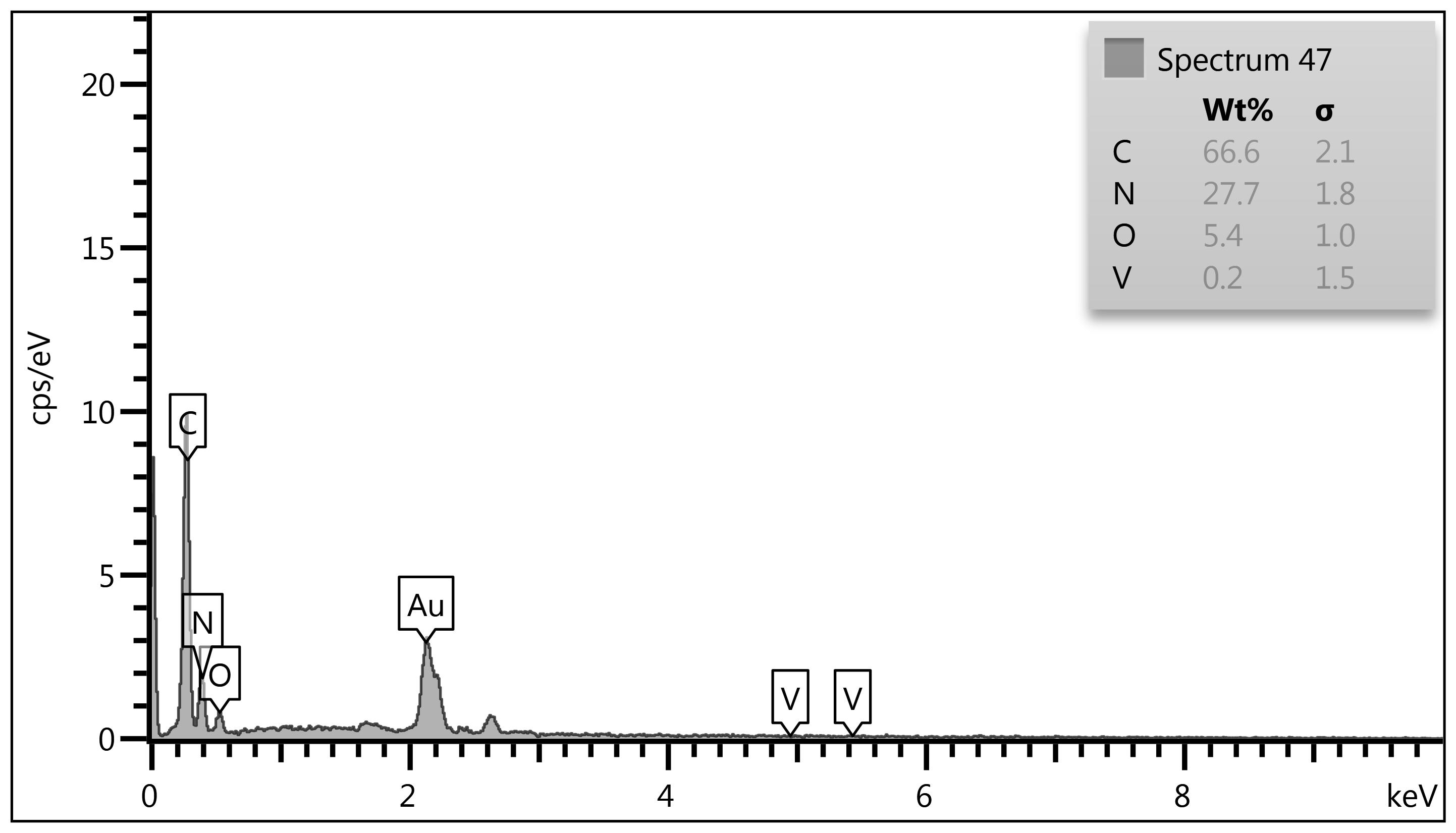  (b) |
| 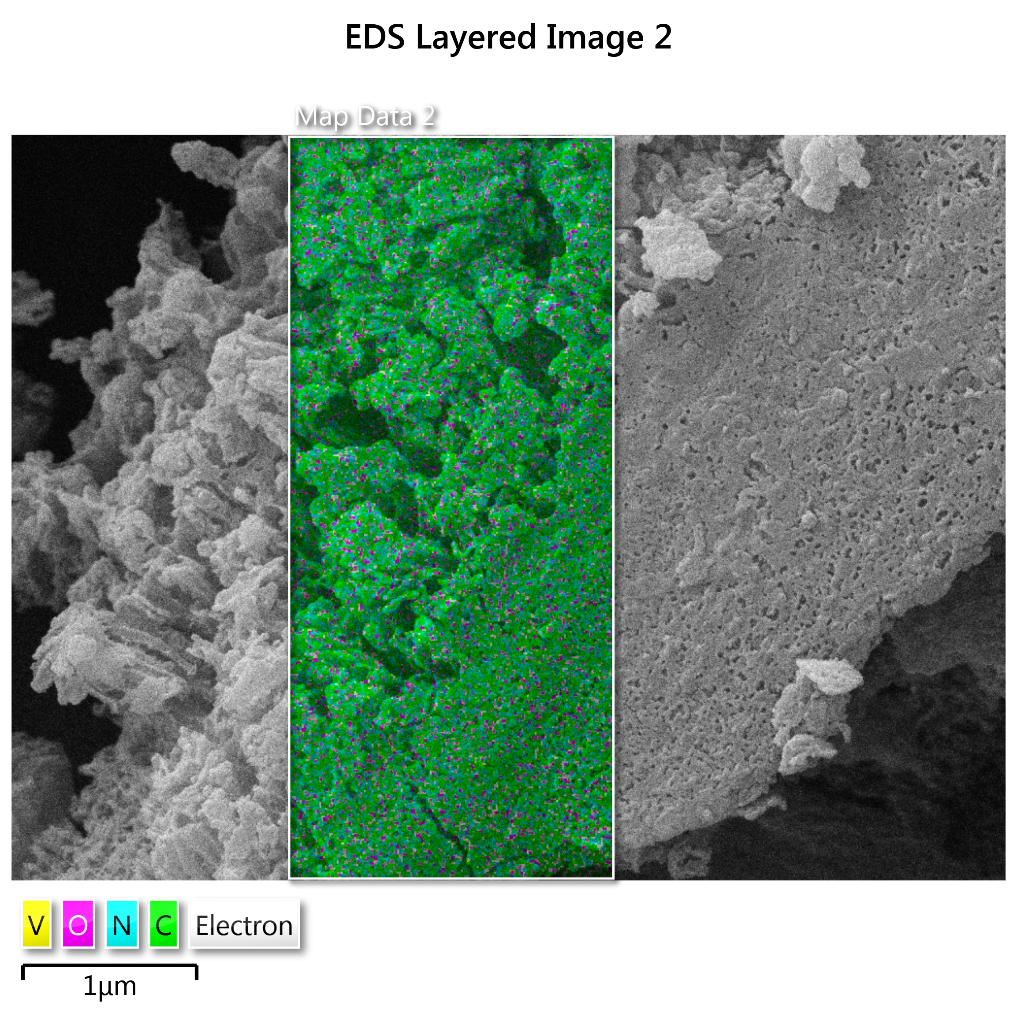  (c) |
| 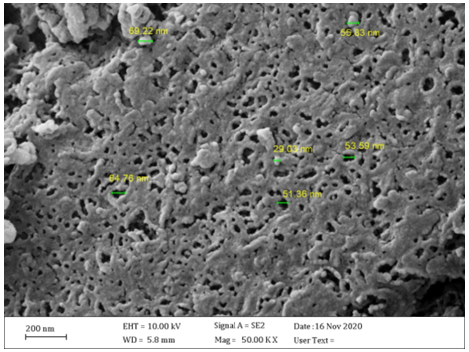  (d) |

**Figure 8S.** UV-Vis (a), EDX (b), SEM equipped with EDX mapping (c), and SEM image (d) of recycled [(VO)TPP][(TCM)_4_].

**Table 1S.** XRD data for [(VO)TPP][(TCM)_4_].

| Entry | 2θ | Peak width [FWHM] (degree) | Size (nm)^a^ | Inter planar distance (nm)^b^ |
| --- | --- | --- | --- | --- |
| 1 | 20.42 | 0.70 | 19.92 | 0.434901 |
| 2 | 21.63 | 0.30 | 46.57 | 0.410838 |
| 3 | 27.44 | 0.77 | 18.34 | 0.325027 |
| 4 | 29.20 | 1.00 | 14.18 | 0.307019 |
| 5 | 45.53 | 0.2 | 74.41 | 0.199221 |
| ^a^ Calculated by Scherrer equation: [D = *Kλ*/(*β* cos *θ*)], D: crystallite size (nm), *K*: shape factor = 0.9, *λ*: Cu radiation (0.154178 nm), *β*: full width at half maximum of the diffraction peak, *θ*: Bragg diffraction angle in degrees; ^b^ Calculated by Bragg equation: *dhkl* = *λ*/(2 sin *θ*). | | | | |

**References**

1. Alabugin, I. V., Gilmore, K. M. & Peterson, P. W. Hyperconjugation. *Wiley Interdisciplinary Rev. Comput. Mol. Sci*. **1**, 109-141 (2011).
2. Deslongchamps, G. & Deslongchamps, P. Bent bonds, the antiperiplanar hypothesis and the theory of resonance. A simple model to understand reactivity in organic chemistry. *Org. Biomol. Chem*. **9**, 5321-5333 (2011).
3. Finch, P. (ed.), *Carbohydrates. Structures, Synthesis and Dynamics*, Kluwer Academic Publishers, The Netherlands, (1999).
4. Lemieux, R.U. & Chu, P. *Abstracts of Papers: 133^rd^ NationalMeeting of the American Chemical Society*, American Chemical Society, Washington, (1958).
5. Edward, J. T. Stability of Glycosides to Acid Hydrolysis. Chem. Ind. (London), **3**, 1102-1104 (1955).
6. (a) Frederickson, L. D. & Hausen, D. M. Infrared Spectra-Structure Correlation Study of Vanadium-Oxygen Compounds. *Anal. Chem*. **35**, 818-827 (1963); (b) Tarama, K., Teranishi, S., Yoshida, S. & Tamura, N. *Proc. 3^rd^ Intern. Cong*. Catalysis, Amsterdam, p. 262 (1965); (c) Kera, Y., Teratani, S. & Hirota, K. [Infrared Spectra of Surface V= O Bond of Vanadium Pentoxide](https://www.journal.csj.jp/doi/pdf/10.1246/bcsj.40.2458). *Bull. Chem. Soc. Japan* **40**, 2458-2458 (1967).
7. (a) Barbosa, G. N., Graeff, C. F. O. & Oliveira, H. P. Thermal annealing effects on vanadium pentoxide xerogel films. *Ecl. Quím., São Paulo*, **30**, 7-15 (2005); (b) Oliveira, H. P., Graeff, C. F. O., Zanta, C. L. P. S., Galina, A. C. & Gonçalves, P. J. [Synthesis, characterization and properties of a melanin-like/vanadium pentoxide hybrid compound](https://pubs.rsc.org/en/content/articlehtml/2000/jm/a908736b). *J. Mater. Chem*. **10**, 371-375 (2000); (c) Gharbi, N. et al. Mixed-valence poly(vanadic acid) gels. *Inorg. Chem*. 21, 2758-2765 (1982).
8. (a) Livage, J. Vanadium pentoxide gels. *Chem. Mater*. **3**, 578-593 (1991); (b) Znaidi, L., Baffier, N. & Huber, M. Synthesis of vanadium bronzes M_x_V_2_O_5_ through sol-gel processes I - Monoclinic bronzes (M = Na, Ag). *Mat. Res. Bull*. **24**, 1501-1514 (1989).
9. (a) Liang, G. Y. et al. Preparation of TiO_2_ photocatalyst loaded with V_2_O_5_ for O_2_ evolution. *J. Cent. South Univ. Technol*. **16**, (2009) 919-925; (b) Bengoa, J. F., Gallegos, N. G., Marchetti, S. G., Alvarez, A. M. & Cagnoli, M. V. Influence of TS-1 structural properties and operation conditions on benzene catalytic oxidation with H_2_O_2_. *Microporous and Mesoporous Mater*. 24, 163-172 (1998).
10. (a) Li, Y., Kuang, J. L., Lu, Y. & Cao. W. B. Facile Synthesis, Characterization of Flower-Like Vanadium Pentoxide Powders and Their Photocatalytic Behavior. *Acta Metallurgica Sinica (English Lett.)* **30**, 1017-1026 (2017); (b) Wang, Y., Li, Z., Sheng, X. & Zhang, Z. Synthesis and optical properties of V2O5 nanorods. *J. Chem. Phys*. **126**, 164701 (2007).
11. Zhu, W. et al. Fabrication and electrochemical sensing performance of a composite film containing a phosphovanadomolybdate and cobalt(II) tetrasulfonate phthalocyanine. *Sens. Actuators B Chem.* **181**, 773-781 (2013).

Figure 9S. The proposed mechanism for the *in situ* oxidation-reduction in the Cannizzaro reaction by unusual hydride transfer *via* the ABO mechanism [31].

Figure 10S. A striking example that had been identified for an unusual hydride transfer from tricyclic orthoamide (A) by the ABO mechanism [32].

Figure 11S. Synthesis of 1,4-dihydropyrano-[2,3-*c*]-pyrazole derivatives *via* ABO mechanism [33a].

Figure 12S. Synthesis of 2,4,6-triarylpyridines by ABO mechanism [33b,c].

Figure 13S. Synthesis of 2-amino-3-cyanopyridines *via* ABO mechanism [33d-f].

Figure 14S. Synthesis of 2-substituted benz-(imida, oxa, and othia)-zole derivatives by ABO mechanism [33g].

Figure 15S. Concerted oxidation *via* hydrogen abstraction-addition mechanism for the synthesis of 1,3,5-trisubstituted pyrazolines [33h].

Figure 16S. Stepwise ABO mechanism for the aromatization of dihydropyridines [33h].

Figure 17S. The synthesis of 2-amino-3,5-dicarbonitrile-6-sulfanylpyridines by ABO mechanism [33i].

Figure 18S. The synthesis of 2-amino-4-aryl-6-(arylamino)pyridine-3,5-dicarbonitriles catalyzed by [TEATNM] as an IL and [TEATCM] as a NMS [33j].

***Selected spectral data analysis for compounds***

*5-Amino-7-(naphthalen-2-yl)-4,5-dihydro-[1,2,4]triazolo[1,5-a]pyrimidine-6-carbonitrile (8a)*: White solid; Yield: 92%; M.p.: 265-266 ^o^C; FT-IR (KBr): (cm^–1^) 3448, 3307, 3173, 2216, 1558, 1293; ^1^H NMR (400 MHz, DMSO-*d_6_*): *δ* (ppm) 6.24 (s, 1H, –CH), 7.27 (s, 2H, –NH_2_), 7.49-7.59 (m, 4H, Ar–H), 7.74 (s, 1H, Ar–H), 7.91-7.92 (d, 1H, –NH), 7.97-7.98 (d, 1H, Ar–H), 8.34-8.36 (d, 1H, Ar–H), 8.76 (s, 1H, =CH); ^13^C NMR (101 MHz, DMSO-*d_6_*): *δ* (ppm) 154.7, 152.5, 147.9, 137.99, 134.2, 130.6, 129.3, 129.1, 126.7, 126.4, 125.9, 125.7, 124.2, 119.3, 56.3, 51.8; HRMS Calcd for C_16_H_12_N_6_ [M+H]^+^: 289.1209, found: 289.12.

*5-Amino-7-(2-chlorophenyl)-4,5-dihydro-[1,2,4]triazolo[1,5-a]pyrimidine-6-carbonitrile (8b)*: Yellow solid; Yield: 91%; M.p.: 270-271 ^o^C; FT-IR (KBr): (cm^–1^) 3392, 3322, 3205, 2192, 1596, 1391; ^1^H NMR (400 MHz, DMSO-*d_6_*): *δ* (ppm) 5.75 (s, 1H, –CH), 7.25 (s, 2H, –NH_2_), 7.37-7.40 (m, 3H, Ar–H), 7.46-7.47 (d, 1H, Ar–H), 7.70 (s, 1H, –NH), 8.66 (s, 1H, =CH); ^13^C NMR (101 MHz, DMSO-*d_6_*): *δ* (ppm) 154.3, 152.3, 147.7, 140.0, 131.9, 130.4, 129.6, 128.3, 118.9, 55.4, 52.5; HRMS Calcd for C_12_H_9_ClN_6_ [M+H]^+^: 273.0664, found: 273.06.

*5-Amino-7-(2-methoxyphenyl)-4,5-dihydro-[1,2,4]triazolo[1,5-a]pyrimidine-6-carbonitrile (8c)*:

Pale yellow solid; Yield: 94%; M.p.: 255-256 ^o^C; FT-IR (KBr): (cm^–1^) 3400, 3324, 3207, 2199, 1615, 1385; ^1^H NMR (400 MHz, DMSO-*d_6_*): *δ* (ppm) 3.68 (s, 3H, –OCH_3_), 5.45 (s, 1H, –CH), 6.90-6.93 (t, 1H, Ar–H), 7.00-7.02 (d, 1H, Ar–H), 7.06 (s, 2H, –NH_2_), 7.10-7.11 (d, 1H, Ar–H), 7.26-7.29 (t, 1H, Ar–H), 7.66 (s, 1H, –NH), 8.41 (s, 1H, =CH); ^13^C NMR (101 MHz, DMSO-*d_6_*): *δ* (ppm), 157.1, 154.7, 151.9, 147.7, 130.9, 129.8, 127.5, 120.8, 119.4, 111.9, 55.9, 50.7; HRMS Calcd for C_13_H_12_N_6_O [M+H]^+^: 269.1189, found: 269.11.

*5-Amino-7-(naphthalen-1-yl)-4,5-dihydro-[1,2,4]triazolo[1,5-a]pyrimidine-6-carbonitrile (8d)*: White solid; Yield: 88%; M.p.: 251-253 ^o^C; FT-IR (KBr): (cm^–1^) 3459, 3367, 3307, 2184, 1655, 1362; ^1^H NMR (400 MHz, DMSO-*d_6_*): *δ* (ppm) 5.52 (s, 1H, –CH), 7.23 (s, 2H, –NH_2_), 7.46-7.48 (dd, 1H, Ar–H), 7.51-7.52 (t, 2H, Ar–H), 7.72 (s, 1H, –NH), 7.75 (d, 1H, Ar–H), 7.89-7.91 (m, 2H, Ar–H), 7.93-7.95 (d, 1H, Ar–H), 8.83 (s, 1H, =CH); ^13^C NMR (101 MHz, DMSO-*d_6_*): *δ* (ppm) 154.4, 152.4, 147.5, 140.8, 133.1, 133.0, 129.2, 128.4, 128.1, 128.0, 126.9, 125.1, 124.9, 119.5, 56.4, 54.9; HRMS Calcd for C_16_H_12_N_6_ [M+H]^+^: 289.1213, found: 289.12.

*5-Amino-7-phenyl-4,5-dihydro-[1,2,4]triazolo[1,5-a]pyrimidine-6-carbonitrile (8e)*: Yellow solid; Yield: 84%; M.p.: 260-262 ^o^C; FT-IR (KBr): (cm^–1^) 3417, 3305, 3141, 2214, 1554, 1348; ^1^H NMR (400 MHz, DMSO-*d_6_*): *δ* (ppm) 5.36 (s, 1H, –CH), 7.24 (s, 2H, –NH_2_), 7.31-7.44 (m, 5H, Ar–H), 7.74 (s, 1H, –NH), 8.81 (d, 1H, =CH); ^13^C NMR (101 MHz, DMSO-*d_6_*): *δ* (ppm) 154.5, 152.4, 147.5, 143.7, 130.2, 129.2, 128.5, 128.3, 126.5, 119.5, 56.5, 54.5; Anal Calcd for C_12_H_10_N_6_: C: 60.00, H: 4.01, N: 34.87%, found: C: 60.50, H: 4.23, N: 35.27%.

*5-Amino-7-(4-fluorophenyl)-4,5-dihydro-[1,2,4]triazolo[1,5-a]pyrimidine-6-carbonitrile (8f)*: White solid; Yield: 90%; M.p.: 281-283 ^o^C; FT-IR (KBr): (cm^–1^) 3501, 3396, 2206, 1609, 1494; ^1^H NMR (400 MHz, DMSO-*d_6_*): *δ* (ppm) 5.60 (s, 1H, –CH), 7.38 (s, 2H, –NH_2_), 7.59-7.62 (dd, 2H, Ar–H), 7.77 (s, 1H, –NH), 8.27-8.30 (dd, 2H, Ar–H), 8.97 (s, 1H, =CH); ^13^C NMR (101 MHz, DMSO-*d_6_*): *δ* (ppm) 154.3, 152.5, 150.6. 147.7, 147.6, 127.9, 124.6, 119.3, 55.4, 53.8; Anal Calcd for C_12_H_9_FN_6_: C: 55.54, H: 3.20, N: 31.88%, found: C: 56.25, H: 3.54, N: 32.80%.

*5-Amino-7-(4-nitrophenyl)-4,5-dihydro-[1,2,4]triazolo[1,5-a]pyrimidine-6-carbonitrile (8g)*: Pale red solid; Yield: 81%; M.p.: 245-247 ^o^C; FT-IR (KBr): (cm^–1^) 3487, 3363, 3117, 2215, 1630, 1493; ^1^H NMR (400 MHz, DMSO-*d_6_*): *δ* (ppm) 5.41 (s, H, –CH), 7.38 (s, 2H, –NH_2_), 7.24-7.35 (d, 2H, Ar–H), 7.24-7.35 (d, 2H, Ar–H), 7.38-8.45 (d, 2H, Ar–H), 7.74 (s, 1H, –NH), 8.80 (s, 1H, =CH); ^13^C NMR (101 MHz, DMSO-*d_6_*): *δ* (ppm) 156.5, 154.3, 152.4, 152.0, 147.5, 139.9, 139.8, 131.8, 131.7, 128.8, 128.7, 119.4, 116.1, 116.0, 115.8, 115.8, 56.4, 53.8; Anal Calcd for C_12_H_9_N_7_O_2_: C: 49.78, H: 2.98, N: 33.58%, found: C: 50.88, H: 3.20, N: 34.62%.

*5-Amino-7-(3,4-dimethoxyphenyl)-[1,2,4]triazolo[1,5-a]pyrimidine-6-carbonitrile (8'a)*: Yellow solid; Yield: 85%; M.p.: 284-286 ^o^C; FT-IR (KBr) (cm^–1^) 3388, 3330, 3171, 2219, 1658, 1493; ^1^H NMR (400 MHz, DMSO-*d_6_*): *δ* (ppm) 3.87-3.88 (s, 6H, –OCH_3_), 7.15-7.17 (d, 1H, Ar–H), 7.48-7.51 (d, 1H, Ar–H), 7.52-7.55 (t, 1H, Ar–H), 8.61 (s, 1H, =CH), 9.92 (s, 2H, –NH_2_); ^13^C NMR (101 MHz, DMSO-*d_6_*): *δ* (ppm) 164.2, 156.4, 152.1, 151.4, 148.8, 129.6, 122.6, 116.5, 112.6, 111.6, 75.5, 56.1, 56.1; HRMS Calcd for C_14_H_12_N_6_O_2_ [M+H]^+^: 297.1128, found: 297.11.

*5-Amino-7-(4-hydroxy-3-methoxyphenyl)-[1,2,4]triazolo[1,5-a]pyrimidine-6-carbonitrile (8'b)*: Yellow solid; Yield: 91%; M.p.: 260-262 ^o^C; FT-IR (KBr) (cm^–1^) 3337, 3237, 3183, 2195, 1530, 1219; ^1^H NMR (400 MHz, DMSO-*d_6_*): *δ* (ppm) 4.07 (s, 3H, –OCH_3_), 7.14-7.16 (d, 1H, Ar–H), 7.61-7.62 (d, 1H, Ar–H), 7.68 (s, 1H, Ar–H), 8.78 (s, 1H, =CH), 9.37 (s, 2H, –NH_2_), 9.96 (s, 1H, –OH); ^13^C NMR (101 MHz, DMSO-*d_6_*): *δ* (ppm) 164.2, 156.3, 155.5, 152.1, 149.7, 147.7, 128.2, 122.9, 116.5, 115.5, 113.3, 73.5, 56.1; HRMS Calcd for C_13_H_10_N_6_O_2_ [M+H]^+^: 283.0979, found: 283.09.

*5-Amino-7-(4-hydroxyphenyl)-[1,2,4]triazolo[1,5-a]pyrimidine-6-carbonitrile (8'c)*: Yellow solid; Yield: 90%; M.p.: 265-266 ^o^C; FT-IR (KBr) (cm^–1^) 3534, 3413, 3313, 3186, 2220, 1591, 1457; ^1^H NMR (400 MHz, DMSO-*d_6_*): *δ* (ppm) 6.89-6.91 (d, 2H, Ar–H), 7.73-7.75 (d, 2H, Ar–H), 8.34 (s, 1H, =CH), 9.12 (s, 2H, –NH_2_), 10.07 (s, 1H, –OH); ^13^C NMR (101 MHz, DMSO-*d_6_*): *δ* (ppm) 164.3, 160.3, 156.3, 155.6, 152.1, 131.1, 127.9, 116.5, 115.5, 75.1; HRMS Calcd for C_12_H_8_N_6_O [M+H]^+^: 253.0844, found: 253.08.

*5-Amino-7-(2-bromophenyl)-[1,2,4]triazolo[1,5-a]pyrimidine-6-carbonitrile (8'd)*: Pale yellow solid; Yield: 90%; M.p.: 232-235 ^o^C; FT-IR (KBr): (cm^–1^) 3429, 3248, 3083, 2188, 1626, 1488; ^1^H NMR (400 MHz, DMSO-*d_6_*): *δ* (ppm) 7.58-7.61 (dd, 1H, Ar–H), 8.21-8.22 (d, 1H, Ar–H), 8.62 (s, 1H, =CH), 7.74-7.75 (d, 2H, Ar–H), 8.98 (s, 1H, Ar–H), 9.36 (s, 2H, –NH_2_); ^13^C NMR (101 MHz, DMSO-*d_6_*): *δ* (ppm) 162.4, 156.5, 155.5, 151.9, 151.7, 149.5, 136.8, 133.3, 123.9, 115.9, 76.4; HRMS Calcd for C_12_H_7_BrN_6_ [M+H]^+^: 313.0766, found: 313.99.

*5-Amino-7-(2-hydroxy-3-methoxyphenyl)-[1,2,4]triazolo[1,5-a]pyrimidine-6-carbonitrile (8'e)*: White solid; Yield: 92%; M.p.: 287-288 ^o^C; FT-IR (KBr): (cm^–1^) 3411, 3345, 3128, 2222, 1645, 1441; ^1^H NMR (400 MHz, DMSO-*d_6_*): *δ* (ppm) 3.88 (s, 3H, –OCH_3_), 6.94-6.97 (d, 1H, Ar–H), 7.41-7.49 (t, 2H, Ar–H), 8.59 (s, 1H, =CH), 9.17(s, 2H, –NH_2_), 9.76 (s, 1H, –OH); ^13^C NMR (101 MHz, DMSO-*d_6_*): *δ* (ppm) 164.3, 156.4, 155.5, 152.1, 149.8, 147.7, 128.2, 122.9, 116.6, 115.6, 113.3, 75.3, 56.2; Anal Calcd for C_13_H_10_N_6_O_2_: C: 55.10, H: 2.99, N: 28.79%, found: C: 55.32, H: 3.57, N: 29.77%.

*5-Amino-7-(4-methoxyphenyl)-[1,2,4]triazolo[1,5-a]pyrimidine-6-carbonitrile (8'f)*: Yellow solid; Yield: 90%; M.p.: 255-256 ^o^C; FT-IR (KBr): (cm^–1^) 3396, 3324, 3165, 2210, 1654, 1490; ^1^H NMR (400 MHz, DMSO-*d_6_*): *δ* (ppm) 3.87 (s, 3H, –OCH_3_), 7.12-7.15 (d, 2H, Ar–H), 7.86-7.89 (d, 2H, Ar–H), 8.60 (s, 1H, =CH), 9.22(s, 2H, –NH_2_); ^13^C NMR (101 MHz, DMSO-*d_6_*): *δ* (ppm) 164.2, 161.7, 156.4, 155.6, 152.1, 130.9, 129.6, 116.4, 114.2, 75.4, 55.9; Anal Calcd for C_13_H_10_N_6_O: C: 57.98, H: 3.19, N: 30.55%, found: C: 58.64, H: 3.79, N: 31.56%.

*5-Amino-7-(3-ethoxy-4-hydroxyphenyl)-[1,2,4]triazolo[1,5-a]pyrimidine-6-carbonitrile (8'g)*: Yellow solid; Yield: 89%; M.p.: 277-279 ^o^C; FT-IR (KBr): (cm^–1^) 3648, 3323, 3196, 2219, 1594, 1271; ^1^H NMR (400 MHz, DMSO-*d_6_*): *δ* (ppm) 1.38-1.42 (t, 3H, –CH_3_), 4.09-4.16 (q, 2H, –OCH_2_), 6.95-6.98 (d, 1H, Ar–H), 7.39-7.47 (d, 1H, Ar–H), 7.48 (s, 1H, Ar–H), 8.59 (s, 1H, =CH), 9.17(s, 2H, –NH_2_), 9.68 (s, 1H, –OH); ^13^C NMR (101 MHz, DMSO-*d_6_*): *δ* (ppm) 164.3, 156.4, 155.6, 152.1, 150.1, 146.8, 128.2, 122.9, 116.6, 115.7, 114.6, 75.3, 64.5, 15.2; Anal Calcd for C_14_H_12_N_6_O_2_: C: 56.20, H: 3.84, N: 27.99%, found: C: 56.75, H: 4.08, N: 28.36%.

*5-Amino-7-(3-hydroxyphenyl)-[1,2,4]triazolo[1,5-a]pyrimidine-6-carbonitrile (8'h)*: Yellow solid; Yield: 91%; M.p.: 275-277 ^o^C; FT-IR (KBr): (cm^–1^) 3403, 3324, 3161, 2213, 1650, 1555, 1382; ^1^H NMR (400 MHz, DMSO-*d_6_*): *δ* (ppm) 6.96-7.00 (d, 1H, Ar–H), 7.26-7.27 (d, 1H, Ar–H), 7.29 (s, 1H, Ar–H), 7.34-7.39 (t, 1H, Ar–H), 8.62 (s, 1H, =CH), 9.24(s, 2H, –NH_2_), 9.81 (s, 1H, –OH); ^13^C NMR (101 MHz, DMSO-*d_6_*): *δ* (ppm) 164.8, 157.7, 156.4, 155.5, 152.1, 138.7, 129.9, 119.9, 118.0, 116.1, 115.9, 75.9; Anal Calcd for C_12_H_8_N_6_O: C: 57.02, H: 3.54, N: 32.87%, found: C: 57.14, H: 3.20, N: 33.32%.

*7-Phenyl-7,12-dihydro-5H-isochromeno[4,3-d][1,2,4]triazolo[1,5-a]pyrimidin-5-one (9a)*: White solid; Yield: 91%; M.p.: 263-265 ^o^C; FT-IR (KBr): (cm^–1^) 3086, 1657, 1566, 1346; ^1^H NMR (400 MHz, DMSO-*d_6_*): *δ* (ppm) 6.50 (s, 1H, –CH), 7.30-7.40 (m, 4H, Ar–H), 7.54-7.59 (m, 2H, Ar–H), 7.59-7.60 (d, 1H, Ar–H), 7.70-8.05 (m, 2H, Ar–H), 8.48 (s, 1H, =CH), 10.49 (s, 1H, –NH); ^13^C NMR (101 MHz, DMSO-*d_6_*): *δ* (ppm) 166.0, 164.9, 152.8, 148.4, 143.7, 134.5, 132.5, 130.0, 124.4, 124.2, 121.9, 121.3, 118.3, 116.5, 104.0, 60.5.

*7-(4-Fluorophenyl)-7,12-dihydro-5H-isochromeno[4,3-d][1,2,4]triazolo[1,5-a]pyrimidin-5-one (9b)*: White solid; Yield: 92%; M.p.: 280-281 ^o^C; FT-IR (KBr): (cm^–1^) 3066, 1670, 1563, 1310; ^1^H NMR (400 MHz, DMSO-*d_6_*): *δ* (ppm) 6.43 (s, 1H, –CH), 7.06-7.12 (t, 2H, Ar–H), 7.23-7.26 (t, 1H, Ar–H), 7.28-7.62 (m, 1H, Ar–H), 7.62-7.65 (t, 2H, Ar–H), 7.96-7.99 (d, 2H, Ar–H), 8.51 (s, 1H, =CH), 10.46 (s, 1H, –NH); ^13^C NMR (101 MHz, DMSO-*d_6_*): *δ* (ppm) 165.4, 165.1, 162.7, 159.5, 152.6, 135.7, 132.6, 129.2, 129.1, 124.4, 124.3, 117.8, 116.6, 115.4, 115.1, 104.9, 60.1.

*7-(4-Bromophenyl)-7,12-dihydro-5H-isochromeno[4,3-d][1,2,4]triazolo[1,5-a]pyrimidin-5-one (9c)*: White solid; Yield: 92%; M.p.: 274-276 ^o^C; FT-IR (KBr): (cm^–1^) 3080, 1669, 1603, 1490, 1352; ^1^H NMR (400 MHz, DMSO-*d_6_*): *δ* (ppm) 6.45 (s, 1H, –CH), 6.46-7.26 (d, 2H, Ar–H), 7.27-7.61 (m, 2H, Ar–H), 7.61-7.64 (d, 2H, Ar–H), 7.96-8.00 (d, 2H, Ar–H), 8.48 (s, 1H, =CH), 10.49 (s, 1H, –NH); ^13^C NMR (101 MHz, DMSO-*d_6_*): *δ* (ppm) 165.4, 165.2, 152.6, 138.9, 132.7, 130.9, 129.2, 128.5, 124.5, 124.3, 117.7, 116.6, 104.6, 60.6.

*7-(3,4-Dimethoxyphenyl)-7,12-dihydro-5H-isochromeno[4,3-d][1,2,4]triazolo[1,5-a]pyrimidin-5-one (9d)*: White solid; Yield: 84%; M.p.: 245-246 ^o^C; FT-IR (KBr): (cm^–1^) 3063, 1670. 1605, 1566, 1351; ^1^H NMR (400 MHz, DMSO-*d_6_*): *δ* (ppm) 3.59-3.73 (s, 6H, –OCH_3_), 6.33 (s, 1H, –CH), 6.69-6.76 (d, 1H, Ar–H), 6.83-6.86 (d, 1H, Ar–H), 7.33 (s, 1H, Ar–H) ,7.35-7.59 (m, 1H, Ar–H), 7.60-7.65 (m, 1H, Ar–H), 7.93-7.96 (d, 2H, Ar–H), 8.45 (s, 1H, =CH), 10.31 (s, 1H, –NH); ^13^C NMR (101 MHz, DMSO-*d_6_*): *δ* (ppm) 165.4, 165.4, 152.6, 149.0, 147.6, 132.4, 124.3, 119.3, 118.2, 116.5, 112.1, 111.8, 104.9, 57.5, 56.1, 55.9.

*7-([1,1'-Biphenyl]-4-yl)-7,12-dihydro-5H-isochromeno[4,3-d][1,2,4]triazolo[1,5-a]pyrimidin-5-one (9e)*: White solid; Yield: 90%; M.p.: 280-282 ^o^C; FT-IR (KBr): (cm^–1^) 3077, 1668, 1603, 1490, 1352; ^1^H NMR (400 MHz, DMSO-*d_6_*): *δ* (ppm) 6.52 (s, 1H, –CH), 7.20-7.22 (d, 2H, Ar–H), 7.30-7.38 (t, 2H, Ar–H), 7.40-7.60 (m, 4H, Ar–H) ,7.60-7.67 (m, 5H, Ar–H), 8.02 (s, 1H, =CH), 10.14 (s, 1H, –NH); ^13^C NMR (101 MHz, DMSO-*d_6_*): *δ* (ppm) 165.5, 165.3, 152.7, 140.5, 139.2, 138.2, 132.7, 129.3, 127.9, 127.6, 127.0, 126.9, 124.5, 124.4, 117.9, 116.6, 104.8, 60.4.

*2-(1H-Indol-3-yl)-4-phenyl-5,6,7,8-tetrahydroquinoline-3-carbonitrile (10a)*: Yellow solid; Yield: 91%; M.p.: 300-301 ^o^C; FT-IR (KBr): (cm^–1^) 3261, 2202, 1614, 1548, 1438; ^1^H NMR (400 MHz, DMSO-*d_6_*): *δ* (ppm) 1.59-1.73 (m, 2H, –CH_2_), 1.74-1.78 (m, 2H, –CH_2_), 2.20-2.24 (t, 2H, –CH_2_), 2.70-2.75 (t, 2H, –CH_2_), 7.26-7.32 (m, 2H, Ar–H), 7.34-7.51 (m, 2H, Ar–H), 7.57-7.60 (m, 1H, Ar–H), 8.14-8.22 (m, 3H, Ar–H), 8.28 (s, 1H, Ar–H), 8.49 (s, 1H, Ar–H), 12.29 (s, 1H, –NH); ^13^C NMR (101 MHz, DMSO-*d_6_*): *δ* (ppm) 181.8, 166.2, 162.9, 151.3, 137.2, 136.6, 133.5, 133.4, 129.6, 129.5, 126.6, 124.1, 1222.9, 121.8, 118.1, 116.9, 116.7, 114.0, 113.0, 111.7, 111.7, 33.3, 31.2, 26.2, 22.9, 22.6; Anal Calcd for C_24_H_19_N_3_: C: 82.08, H: 4.95, N: 12.08%, found: C: 82.49, H: 5.48, N: 12.03%.

*2-(1H-Indol-3-yl)-4-(naphthalen-2-yl)-5,6,7,8-tetrahydroquinoline-3-carbonitrile (10b)*: Pale yellow solid; Yield: 84%; M.p.: 287-288 ^o^C; FT-IR (KBr): (cm^–1^) 3224, 1645, 1525, 1440, 1240; ^1^H NMR (400 MHz, DMSO-d_6_): δ (ppm) 1.62-1.63 (m, 2H, –CH_2_), 1.75-1.76 (m, 2H, –CH_2_), 2.18-2.21 (t, 2H, –CH_2_), 2.72-2.76 (t, 2H, –CH_2_), 7.28-7.36 (m, 2H, Ar–H), 7.58-7.73 (m, 3H, Ar–H), 8.03-8.05 (m, 2H, Ar–H), 8.12-8.15 (d, 1H, Ar–H), 8.23-8.28 (m, 2H, Ar–H), 8.42 (s, 1H, Ar–H), 8.54-8.57 (t, 2H, Ar–H), 12.29 (s, 1H, –NH); ^13^C NMR (101 MHz, DMSO-*d_6_*): *δ* (ppm) 181.9, 161.6, 158.4, 152.5, 137.2, 136.5, 134.9, 133.4, 132.9, 130.5, 129.2, 129.1, 128.3, 127.8, 126.6, 125.4, 124.1, 122.9, 121.9, 118.4, 114.1, 113.0, 111.9, 33.3, 26.2, 22.8, 22.5; Anal. Calcd. For C_28_H_21_N_3_: C: 84.10; H: 5.21; N: 10.48%; Found C: 84.18; H: 5.30; N: 10.52%.

*2,4-Di(1H-indol-3-yl)-5,6,7,8-tetrahydroquinoline-3-carbonitrile (10c)*: Pale red solid; Yield: 92%; M.p.: 310-312 ^o^C; FT-IR (KBr): (cm^–1^) 3341, 2199, 1633, 1508, 1278; ^1^H NMR (400 MHz, DMSO-d_6_): δ (ppm) 1.58-1.62 (m, 2H, –CH_2_), 1.73-1.77 (m, 2H, –CH_2_), 2.19-2.23 (t, 2H, –CH_2_), 2.70-2.74 (t, 2H, –CH_2_), 7.24-7.57 (m, 4H, Ar-H), 7.59-7.61 (m, 2H, Ar-H), 7.95-7.97 (d, 1H, Ar-H), 8.30-8.33 (d, 1H, Ar-H), 8.55-8.56 (m, 1H, Ar–H), 8.68-8.70 (d, 2H, Ar–H), 12.18 (s, 1H, –NH), 12.52 (s, 1H, –NH); ^13^C NMR (101 MHz, DMSO-*d_6_*): *δ* (ppm) 181.3, 161.6, 158.4, 145.7, 136.9, 136.7, 134.4, 131.7, 127.9, 127.0, 123.9, 123.8, 122.8, 122.5, 122.3, 122.2, 119.0, 114.9, 113.3, 112.9, 110.9, 102.3, 33.3, 31.2, 26.3, 22.9, 22.5; Anal. Calcd. For C_26_H_20_N_4_: C: 79.57; H: 4.88; N: 14.08%; Found C: 80.39; H: 5.19; N: 14.42%.

*2-(1H-Indol-3-yl)-4-(naphthalen-1-yl)-5,6,7,8-tetrahydroquinoline-3-carbonitrile (10d)*: Yellow solid; Yield: 90%; M.p.: 271-272 ^o^C; FT-IR (KBr): (cm^–1^) 3350, 2198, 1632, 1507, 1427, 1278; ^1^H NMR (400 MHz, DMSO-d_6_): δ (ppm) 1.61-1.62 (m, 2H, –CH_2_), 1.74-1.75 (m, 2H, –CH_2_), 2.18-2.20 (t, 2H, –CH_2_), 2.72-2.74 (t, 2H, –CH_2_), 7.32-7.38 (m, 2H, Ar–H), 7.63-7.60 (m, 3H, Ar–H), 7.70-7.75 (d, 1H, Ar–H), 8.05-8.08 (m, 1H, Ar–H), 8.12-8.13 (d, 1H, Ar–H), 8.15-8.18 (m, 1H, Ar–H), 8.25-8.28 (m, 1H, Ar–H), 8.33-8.36 (s, 1H, Ar–H), 8.60-8.61 (d, 1H, Ar–H), 8.97 (s, 1H, Ar–H), 12.41 (s, 1H, –NH); ^13^C NMR (101 MHz, DMSO-*d_6_*): *δ* (ppm) 181.5, 161.6, 158.4, 150.9, 137.4, 137.3, 136.7, 133.5, 132.4, 131.3, 130.2, 129.2, 127.9, 126.6, 125.2, 124.3, 124.1, 123.0, 121.9, 117.9, 115.8, 114.4, 113.0, 33.3, 31.2, 26.2, 22.9 22.5; Anal. Calcd. For C_28_H_21_N_3_: C: 83.41; H: 4.59; N: 10.10%; Found C: 84.18; H: 5.30; N: 10.52%.

**FT-IR, ^1^H NMR, ^13^C NMR, CHN, and HRMS spectra**

**Figure 19S.** FT-IR spectrum of *5-amino-7-(naphthalen-2-yl)-4,5-dihydro-[1,2,4]triazolo[1,5-a]pyrimidine-6-carbonitrile (8a)*

^^
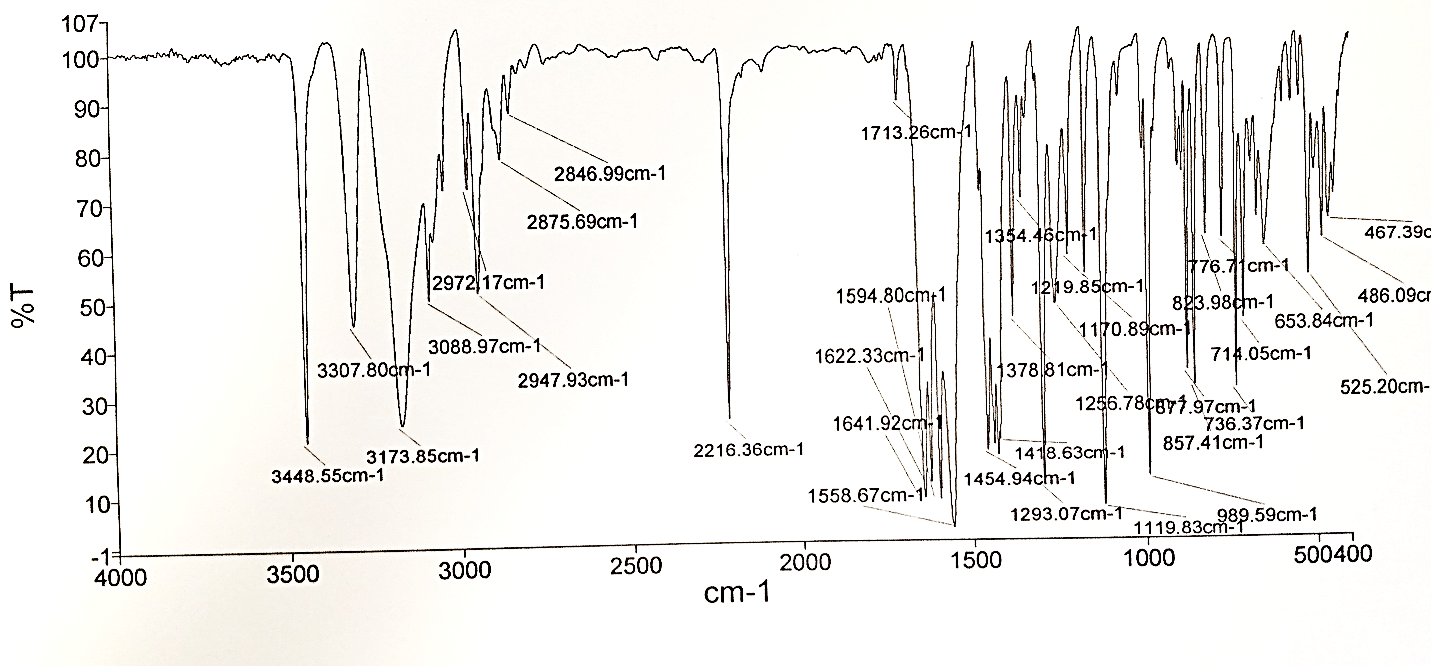


**Figure 20S.** ^1^H NMR spectrum of *5-amino-7-(naphthalen-2-yl)-4,5-dihydro-[1,2,4]triazolo[1,5-a]pyrimidine-6-carbonitrile (8a)*

^
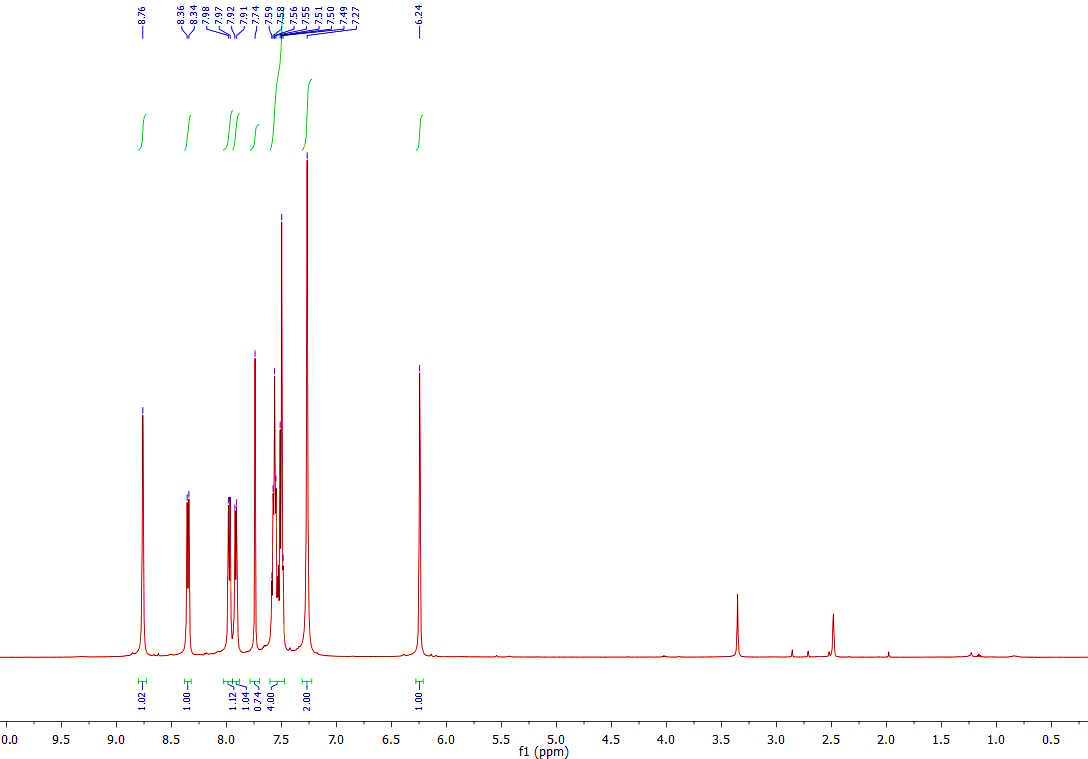
^

^
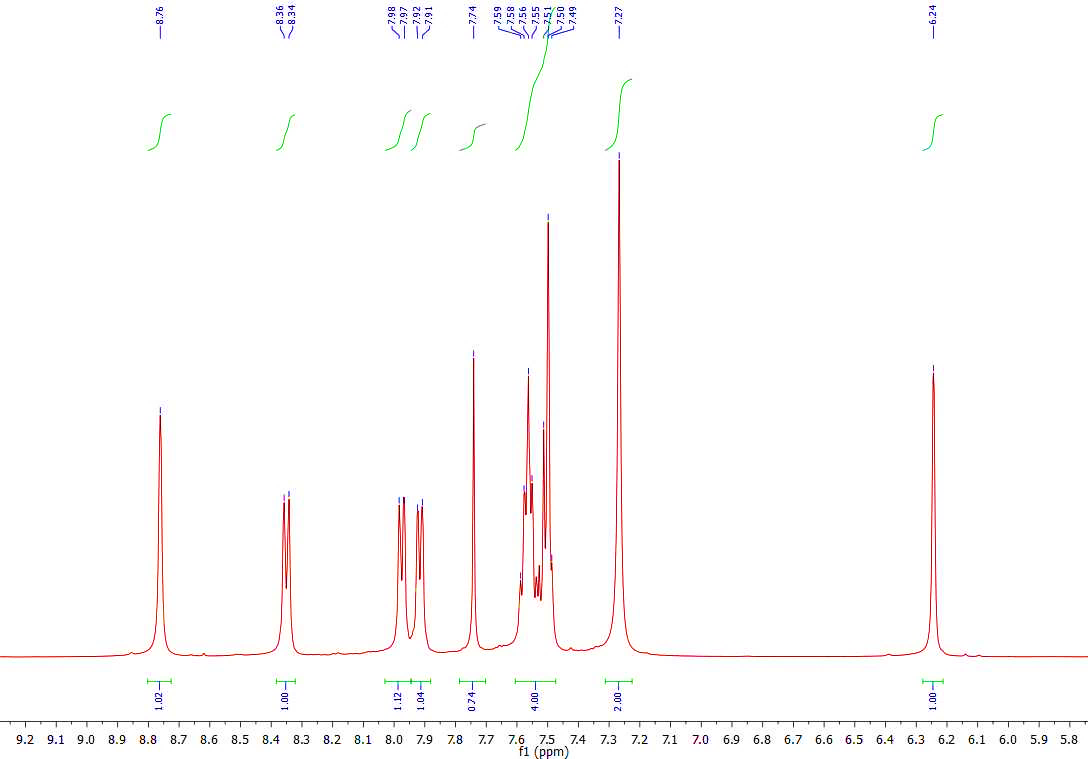
^

**Figure 21S.** ^13^C NMR spectrum of *5-amino-7-(naphthalen-2-yl)-4,5-dihydro-[1,2,4]triazolo[1,5-a]pyrimidine-6-carbonitrile (8a)*


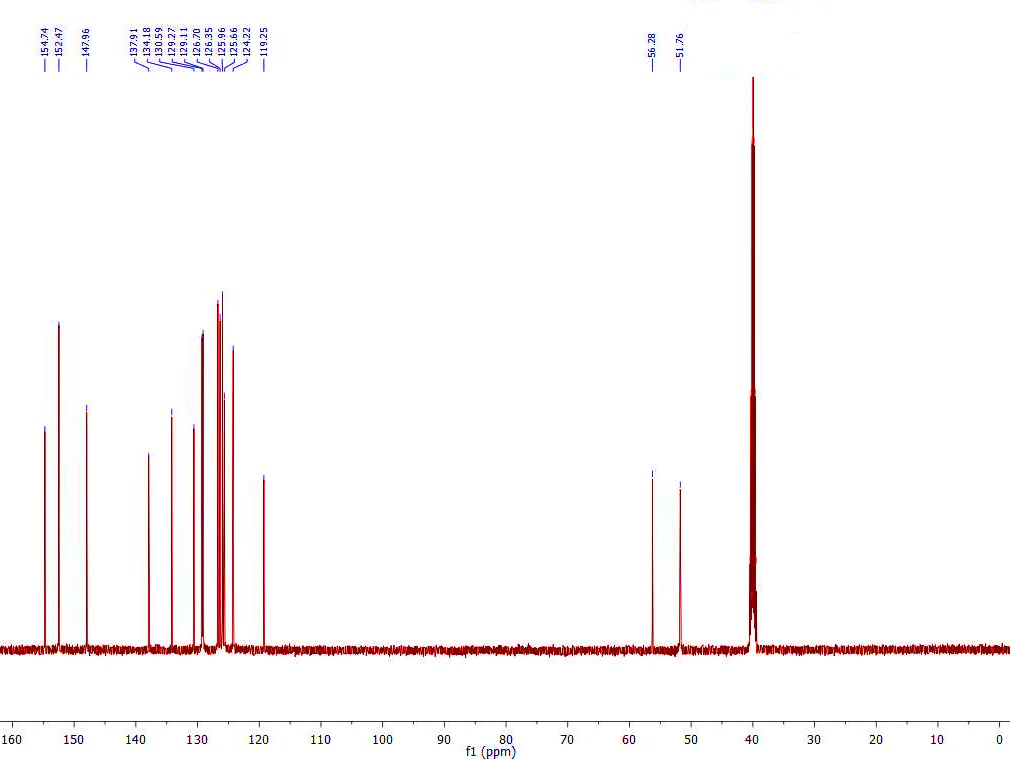


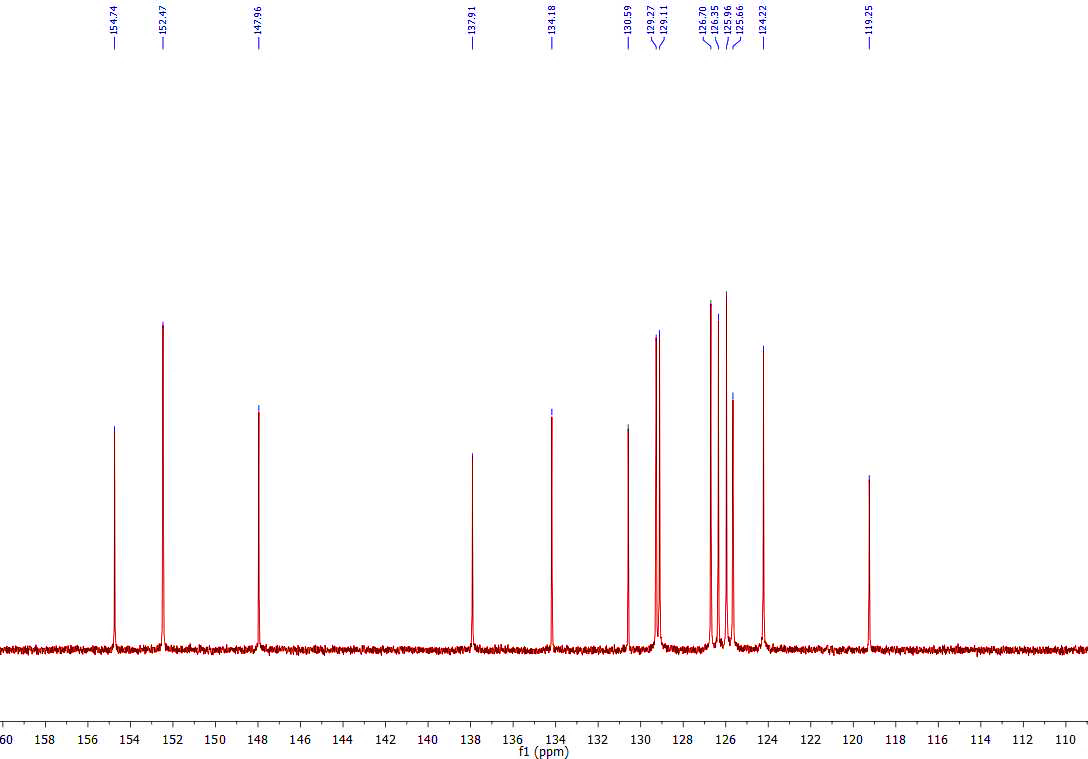


**Figure 22S.** HRMS of *5-amino-7-(naphthalen-2-yl)-4,5-dihydro-[1,2,4]triazolo[1,5-a]pyrimidine-6-carbonitrile (8a)*

**Figure 23S.** FT-IR spectrum of *5-amino-7-(2-chlorophenyl)-4,5-dihydro-[1,2,4]triazolo[1,5-a]pyrimidine-6-carbonitrile (8b)*

^^
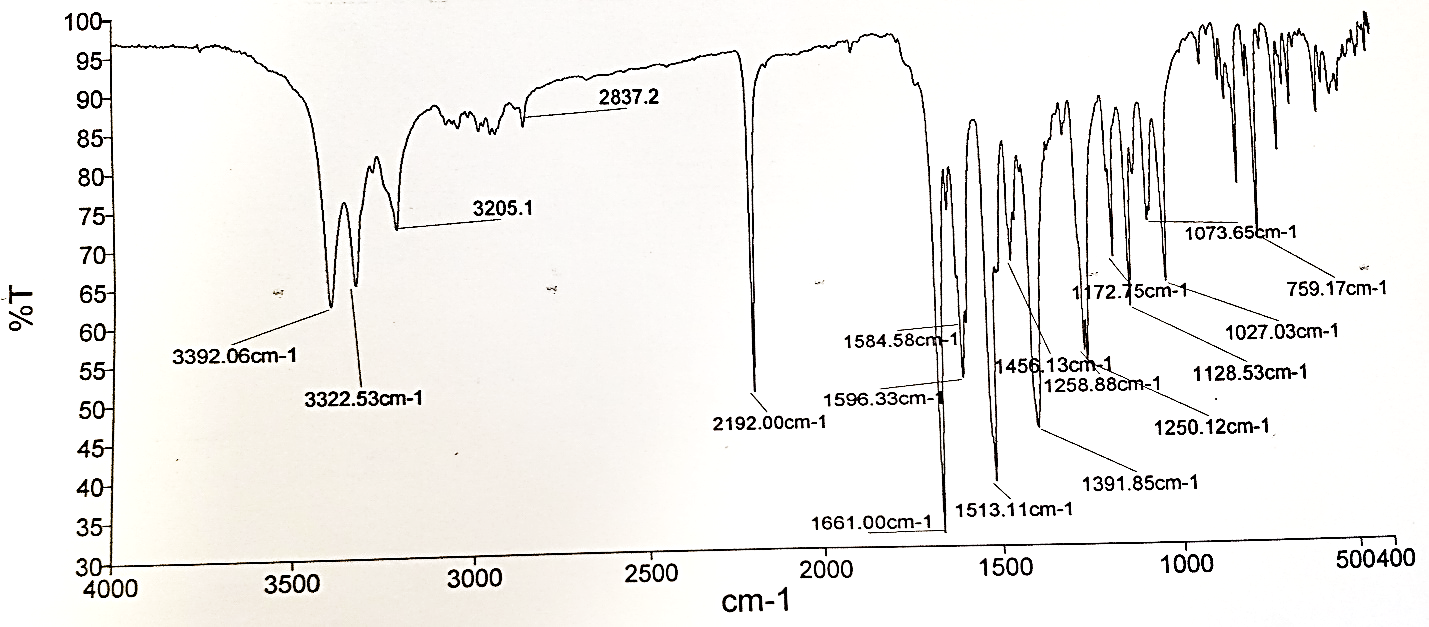


**Figure 24S.** ^1^H NMR spectrum of *5-amino-7-(2-chlorophenyl)-4,5-dihydro-[1,2,4]triazolo[1,5-a]pyrimidine-6-carbonitrile (8b)*


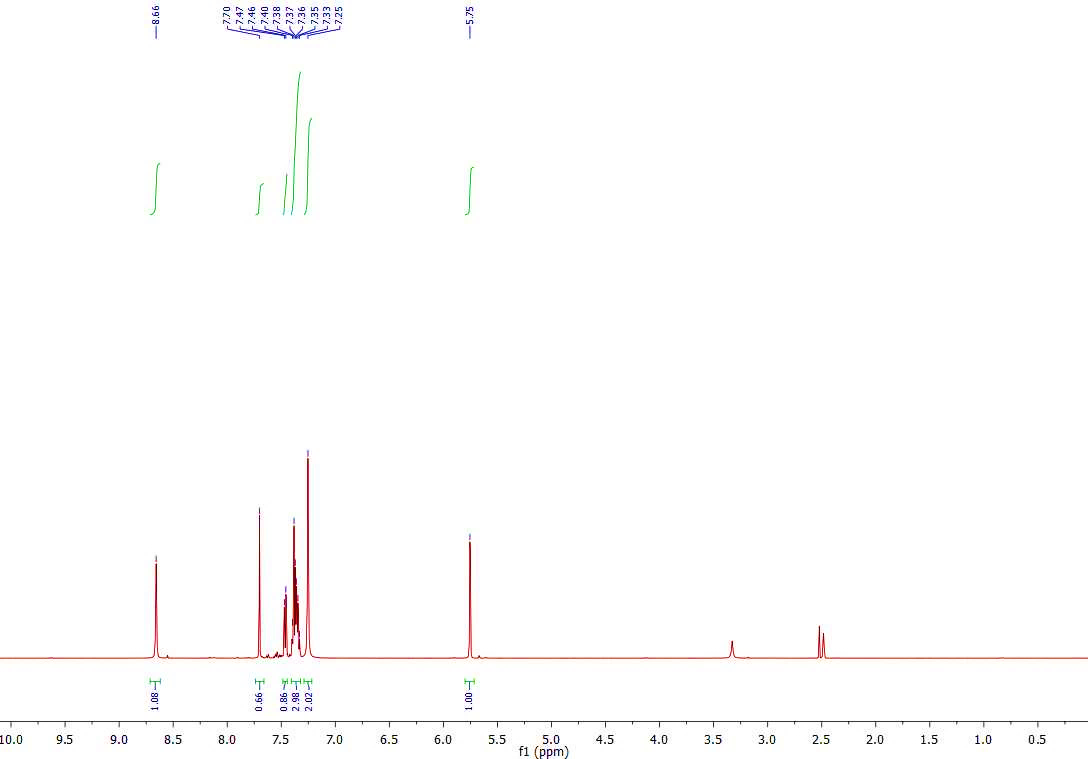


**Figure 25S.** ^13^C NMR spectrum of *5-amino-7-(2-chlorophenyl)-4,5-dihydro-[1,2,4]triazolo[1,5-a]pyrimidine-6-carbonitrile (8b)*


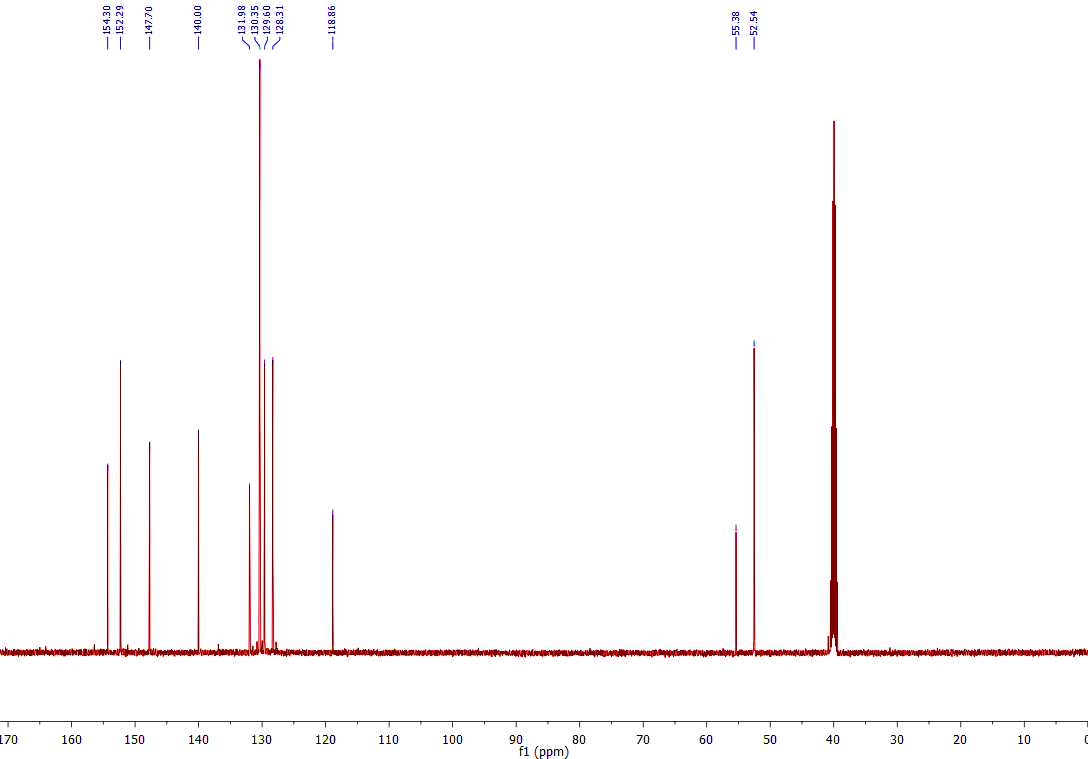


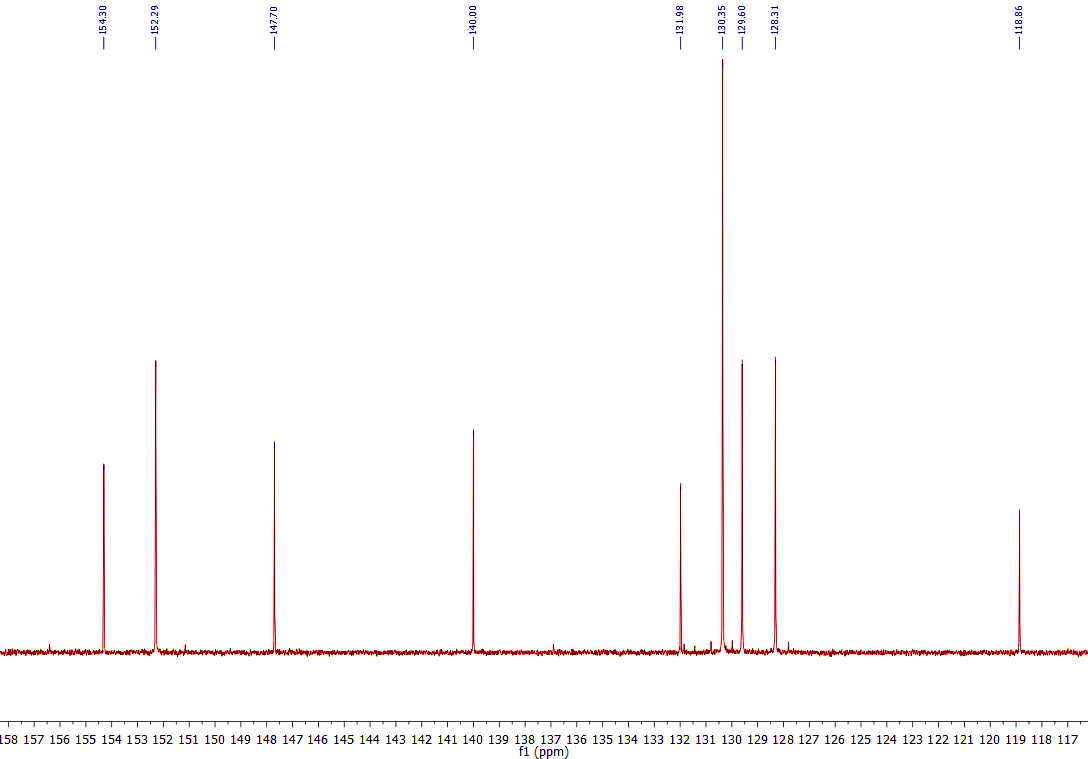


**Figure 26S.** HRMS of *5-amino-7-(2-chlorophenyl)-4,5-dihydro-[1,2,4]triazolo[1,5-a]pyrimidine-6-carbonitrile (8b)*

**Figure 27S.** FT-IR spectrum of *5-amino-7-(2-methoxyphenyl)-4,5-dihydro-[1,2,4]triazolo[1,5-a]pyrimidine-6-carbonitrile (8c)*


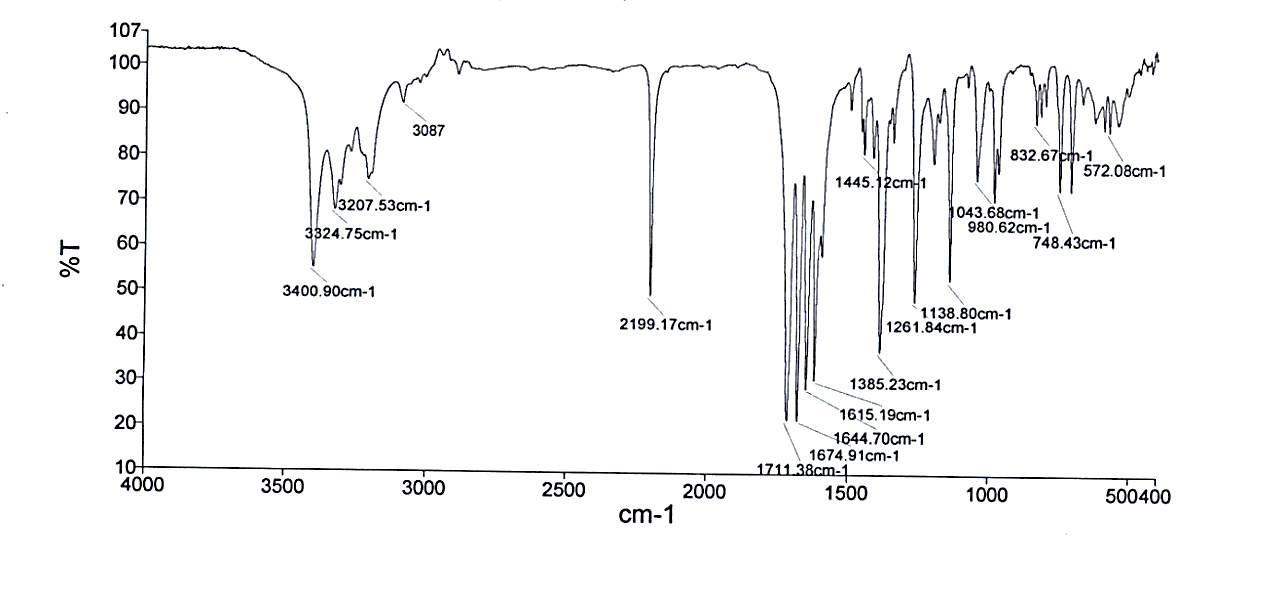


**Figure 28S.** ^1^H NMR spectrum of *5-amino-7-(2-methoxyphenyl)-4,5-dihydro-[1,2,4]triazolo[1,5-a]pyrimidine-6-carbonitrile (8c)*


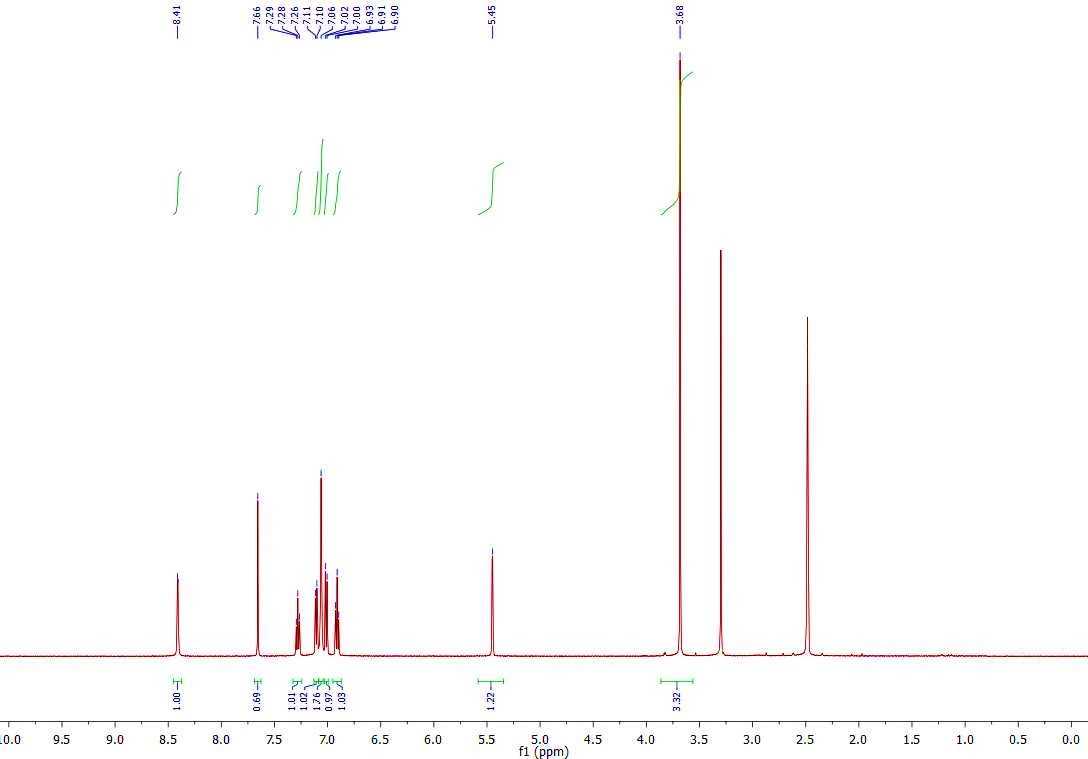


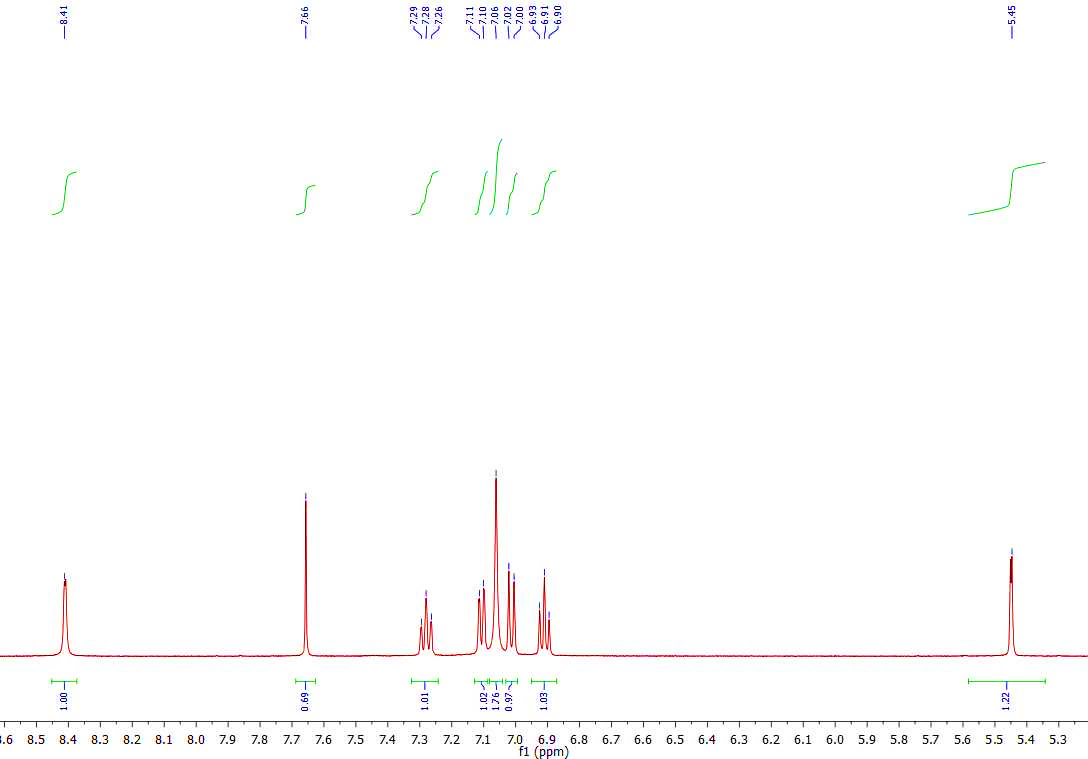

^
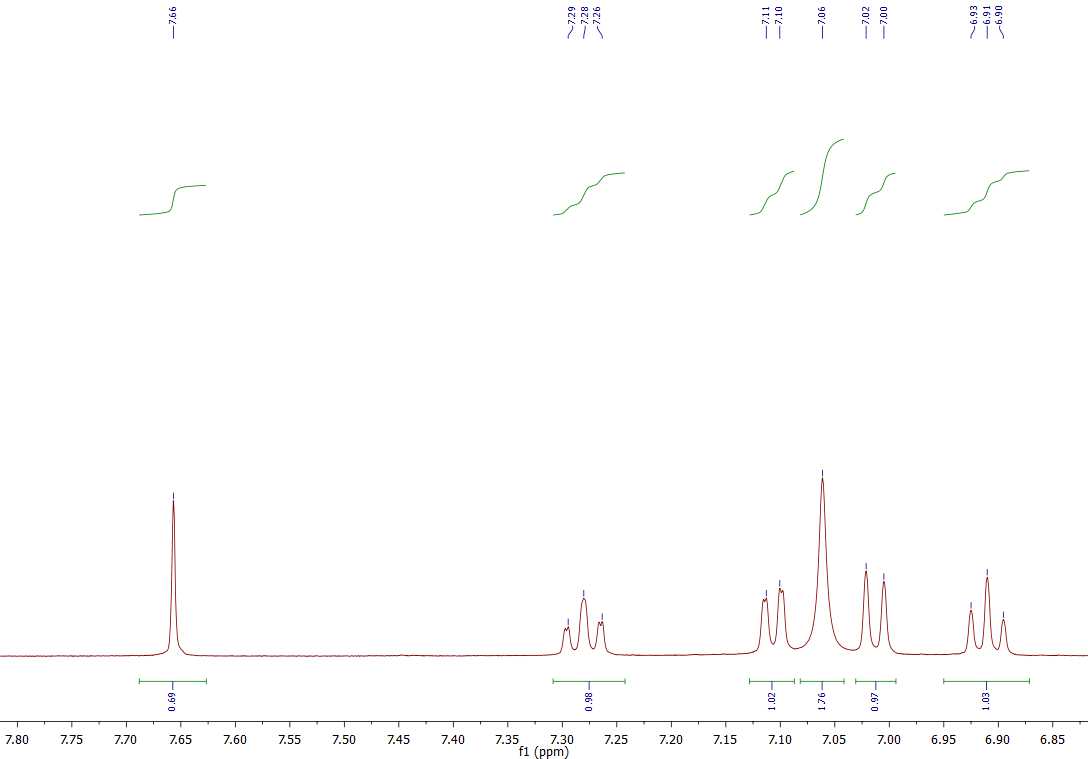
^

**Figure 29S.** ^13^C NMR spectrum of *5-amino-7-(2-methoxyphenyl)-4,5-dihydro-[1,2,4]triazolo[1,5-a]pyrimidine-6-carbonitrile (8c)*


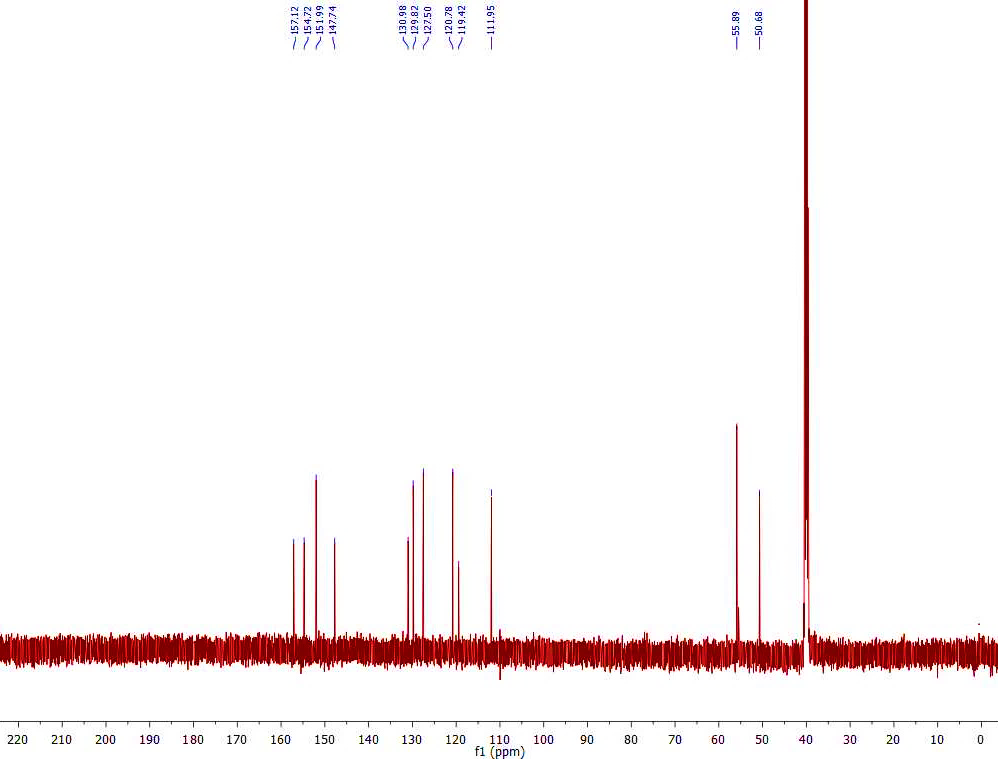


**Figure 30S.** HRMS of *5-amino-7-(2-methoxyphenyl)-4,5-dihydro-[1,2,4]triazolo[1,5-a]pyrimidine-6-carbonitrile (8c)*

**Figure 31S.** FT-IR spectrum of *5-amino-7-(naphthalen-1-yl)-4,5-dihydro-[1,2,4]triazolo[1,5-a]pyrimidine-6-carbonitrile (8d)*

**^
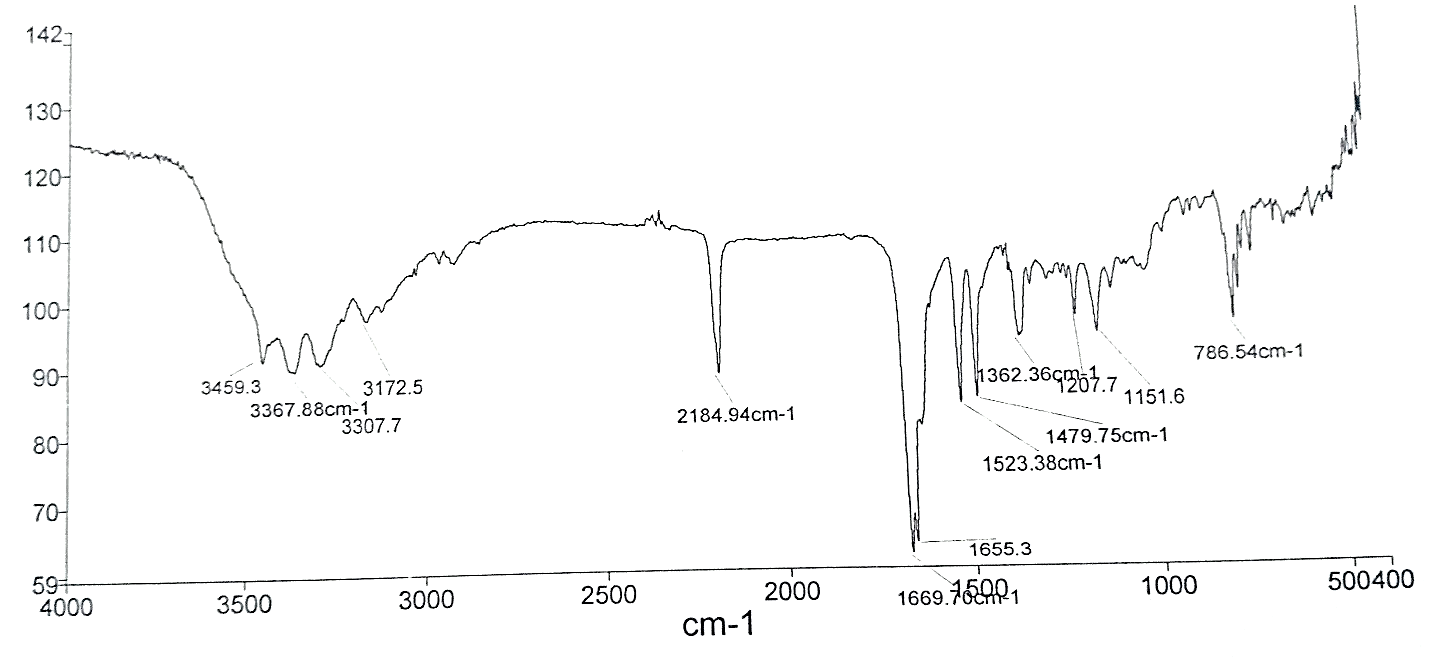
^**

**Figure 32S.** ^1^H NMR spectrum of *5-amino-7-(naphthalen-1-yl)-4,5-dihydro-[1,2,4]triazolo[1,5-a]pyrimidine-6-carbonitrile (8d)*


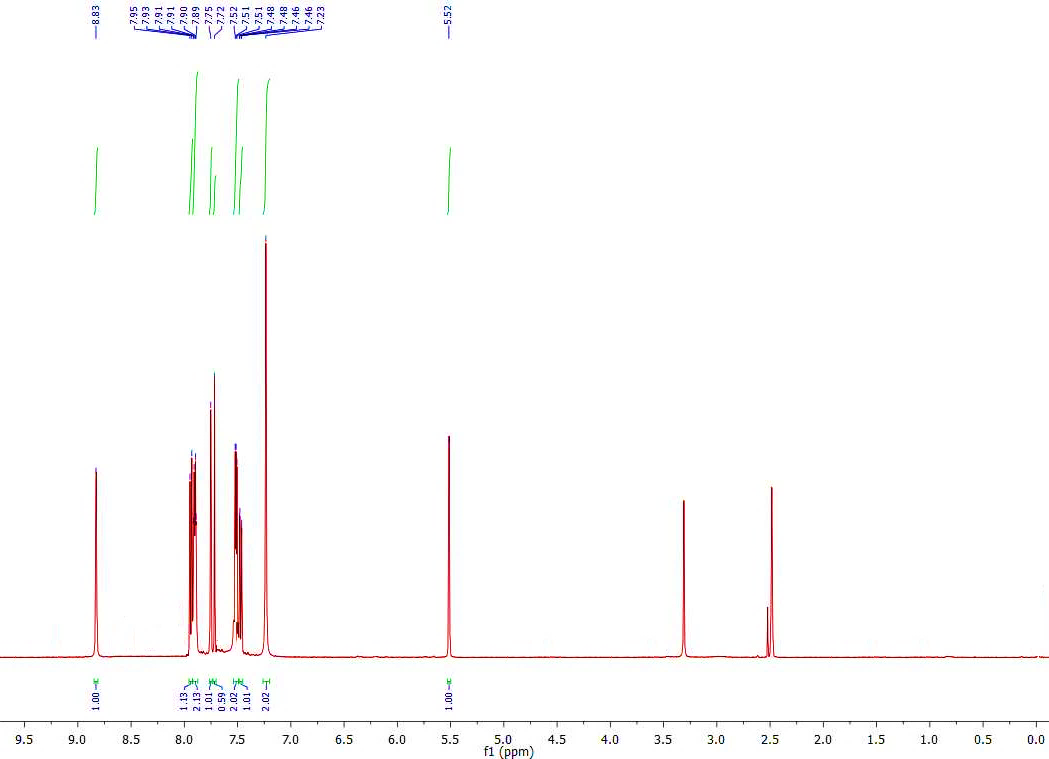


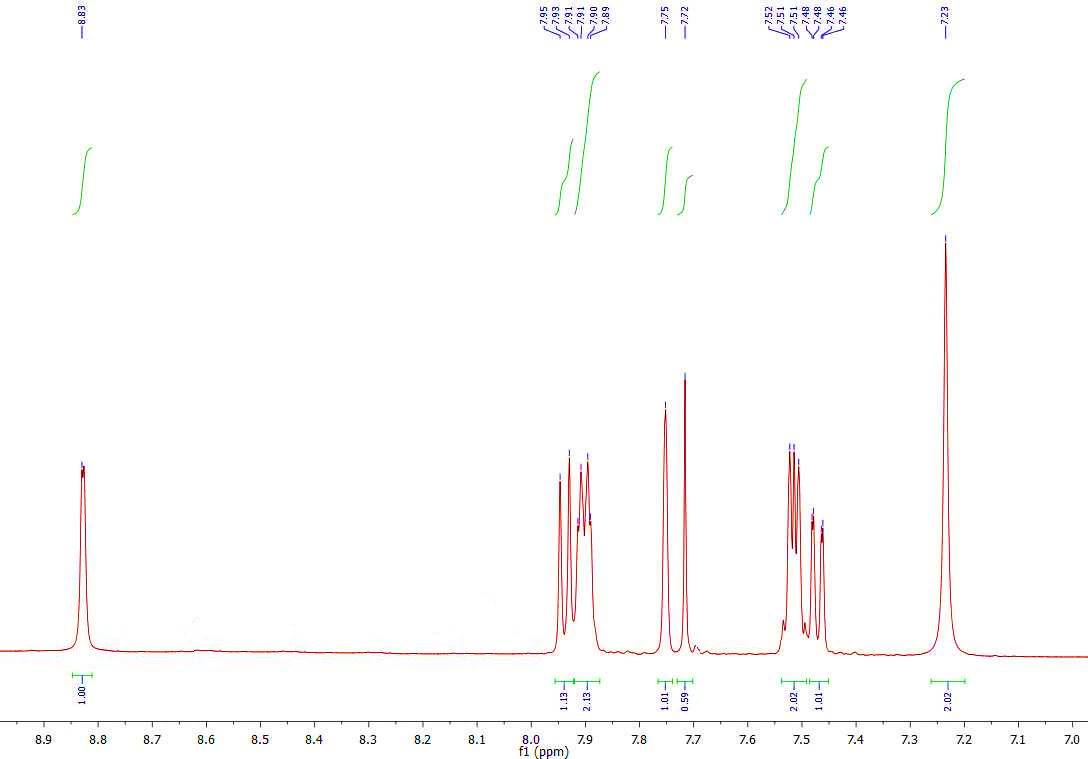


**Figure 33S.** ^13^C NMR spectrum of *5-amino-7-(naphthalen-1-yl)-4,5-dihydro-[1,2,4]triazolo[1,5-a]pyrimidine-6-carbonitrile (8d)*

^^
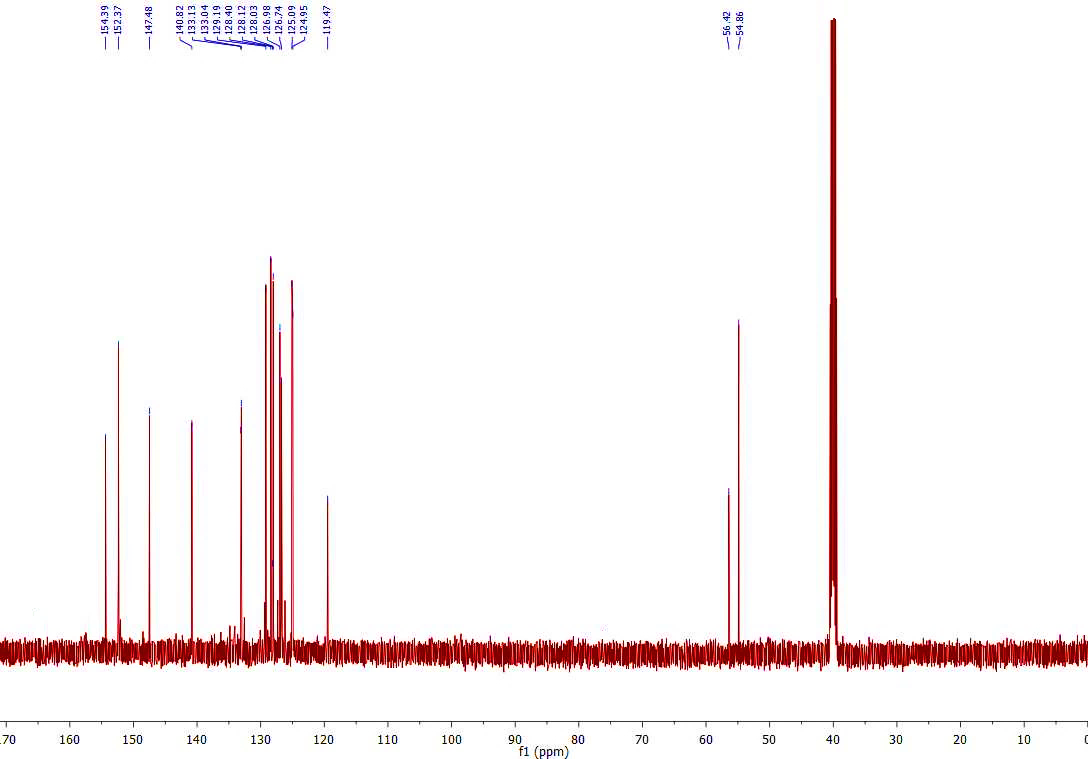


^^
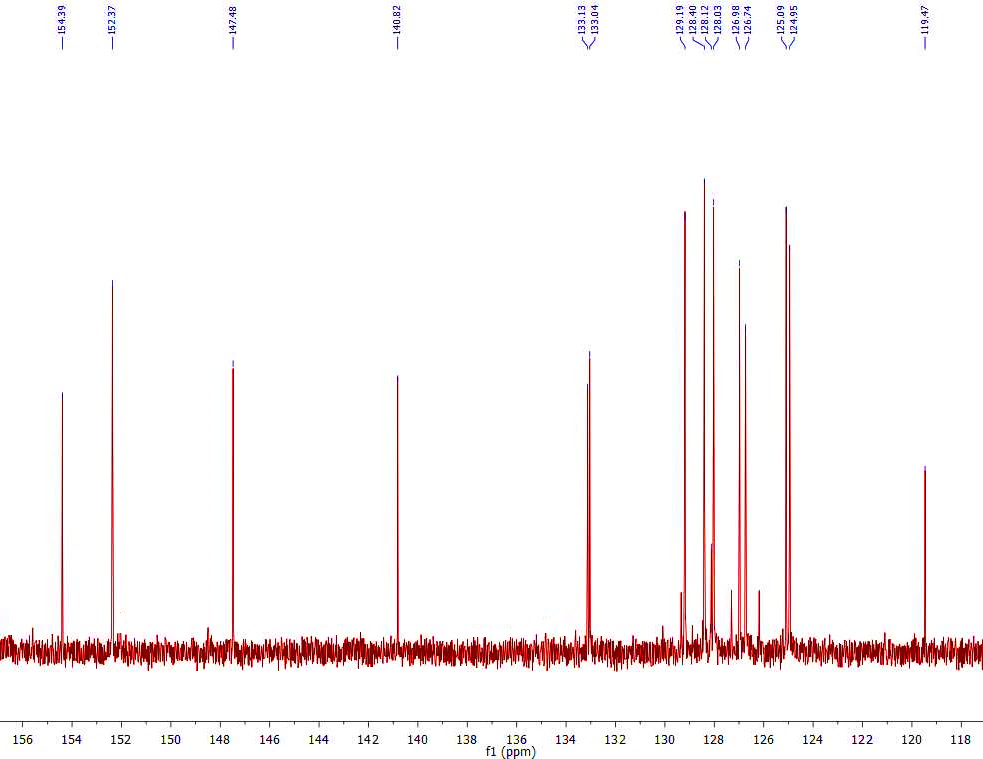


**Figure 34S.** HRMS spectrum of *5-amino-7-(naphthalen-1-yl)-4,5-dihydro-[1,2,4]triazolo[1,5-a]pyrimidine-6-carbonitrile (8d)*

**Figure 35S.** FT-IR spectrum of *5-amino-7-phenyl-4,5-dihydro-[1,2,4]triazolo[1,5-a]pyrimidine-6-carbonitrile (8e)*


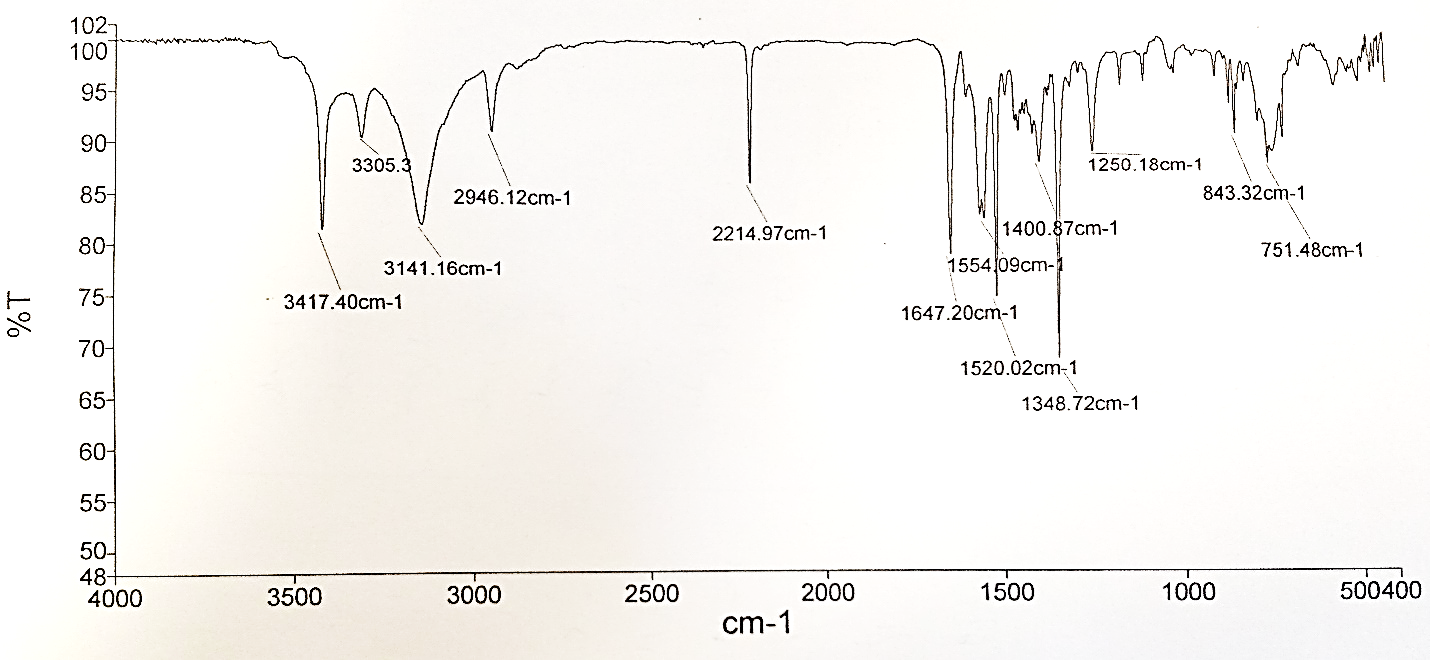


**Figure 36S.** ^1^H NMR spectrum of *5-amino-7-phenyl-4,5-dihydro-[1,2,4]triazolo[1,5-a]pyrimidine-6-carbonitrile (8e)*

^
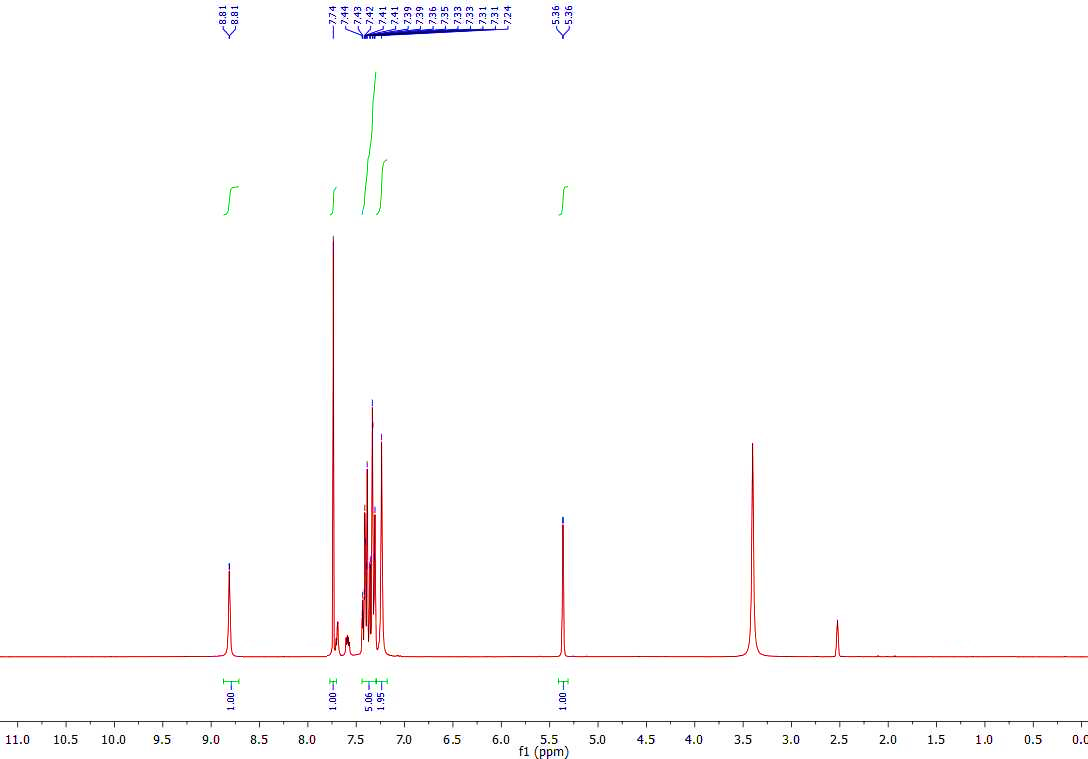
^

**Figure 37S.** ^13^C NMR spectrum of *5-amino-7-phenyl-4,5-dihydro-[1,2,4]triazolo[1,5-a]pyrimidine-6-carbonitrile (8e)*

^
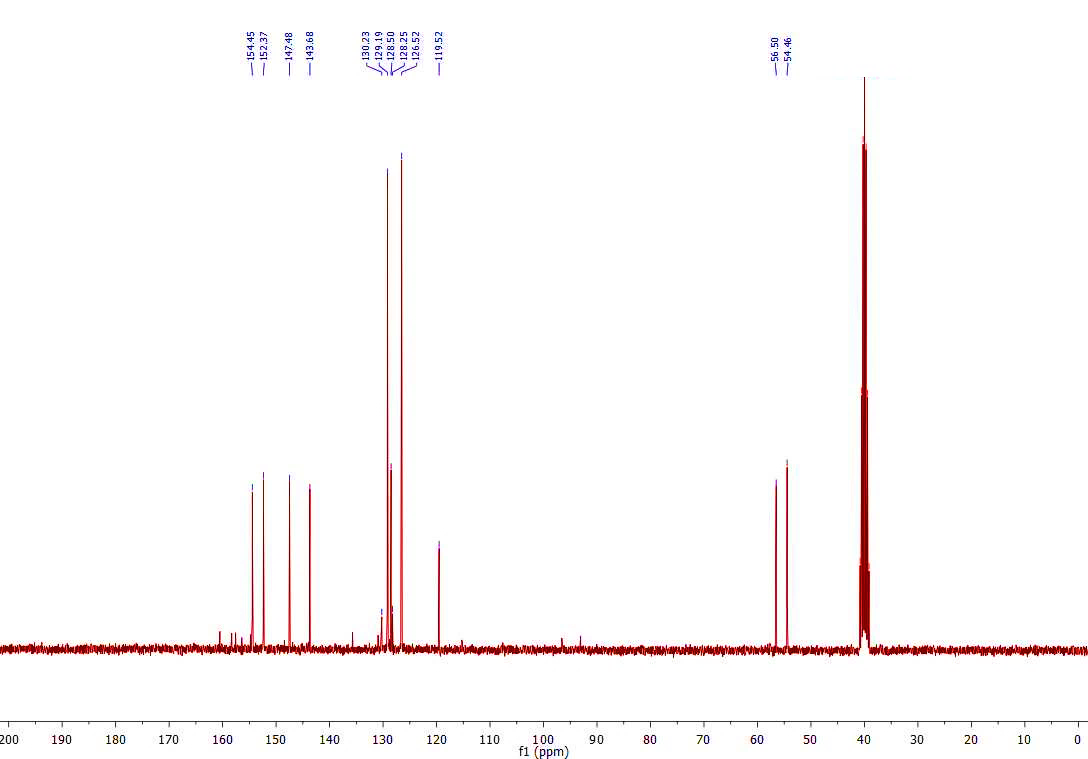
^

**Figure 38S.** FT-IR spectrum of *5-amino-7-(4-fluorophenyl)-4,5-dihydro-[1,2,4]triazolo[1,5-a]pyrimidine-6-carbonitrile (8f)*

^^
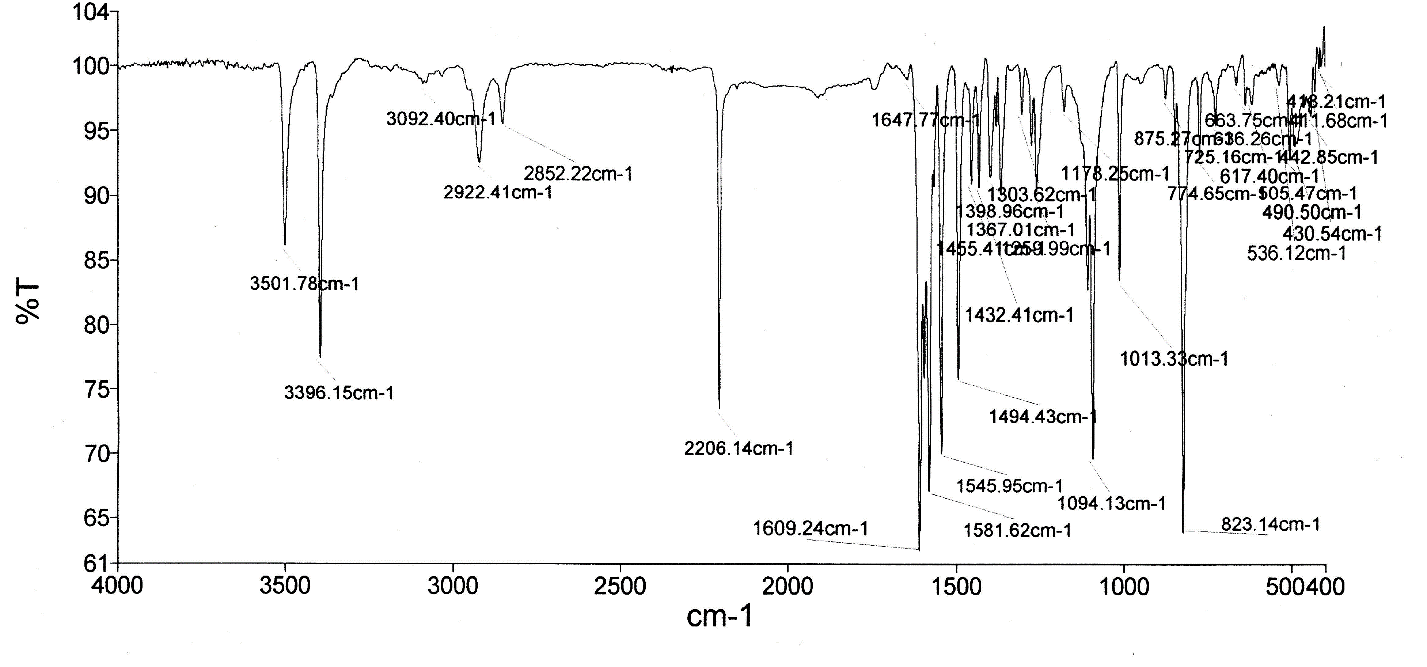


**Figure 39S.** ^1^H NMR spectrum of *5-amino-7-(4-fluorophenyl)-4,5-dihydro-[1,2,4]triazolo[1,5-a]pyrimidine-6-carbonitrile (8f)*

^
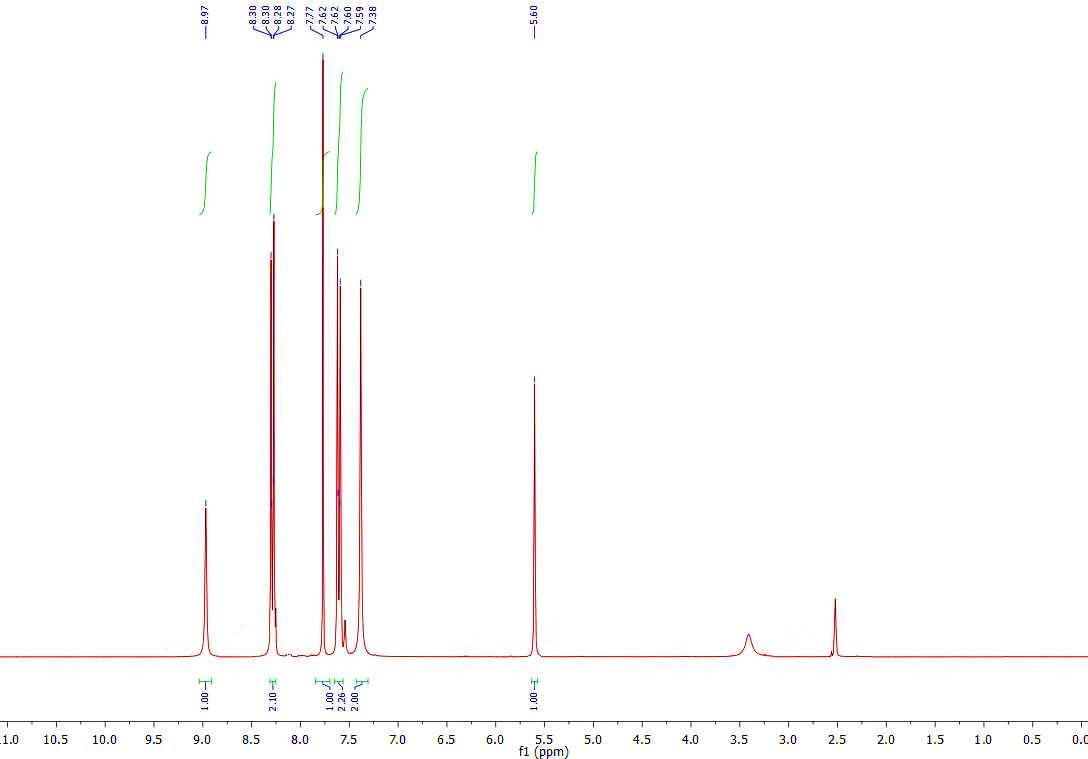
^

^
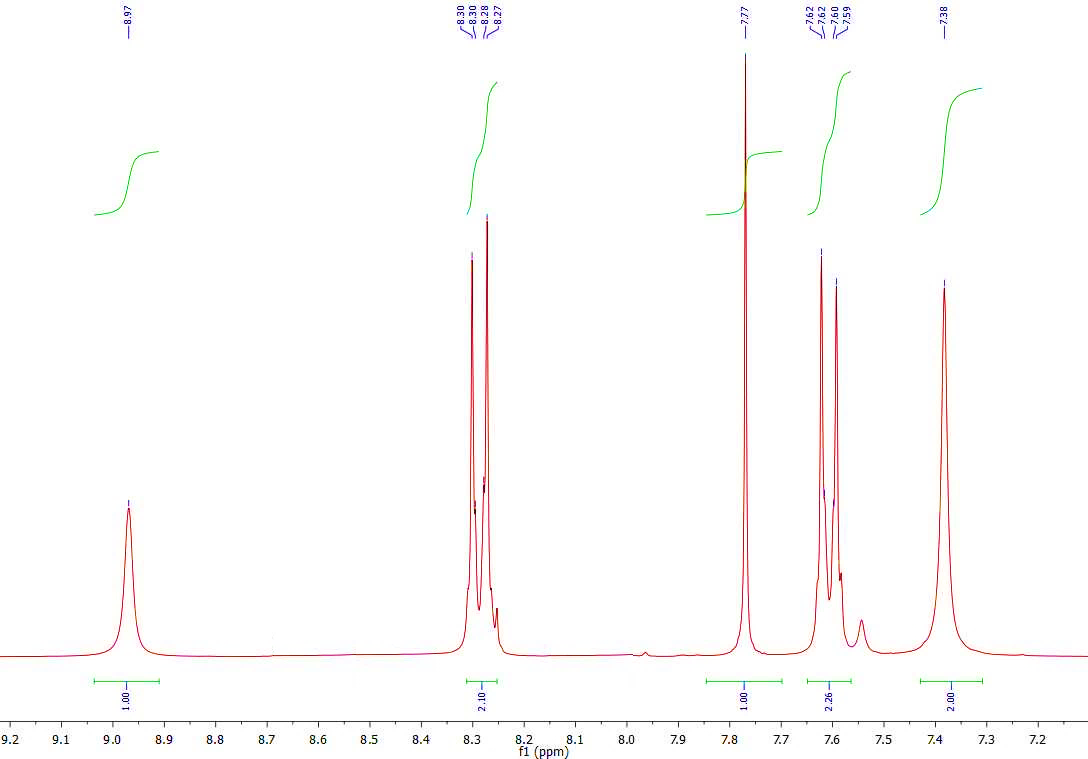
^

**Figure 40S.** ^13^C NMR spectrum of *5-amino-7-(4-fluorophenyl)-4,5-dihydro-[1,2,4]triazolo[1,5-a]pyrimidine-6-carbonitrile (8f)*

^^
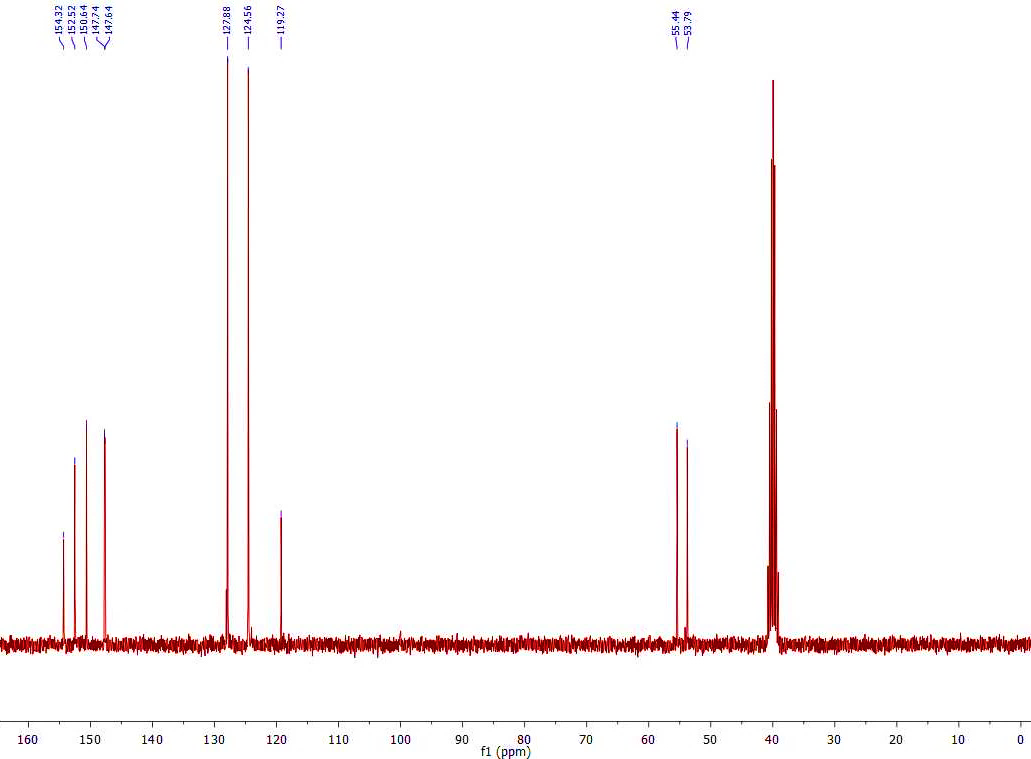


**Figure 41S.** FT-IR spectrum of *5-amino-7-(4-nitrophenyl)-4,5-dihydro-[1,2,4]triazolo[1,5-a]pyrimidine-6-carbonitrile (8g)*

^^
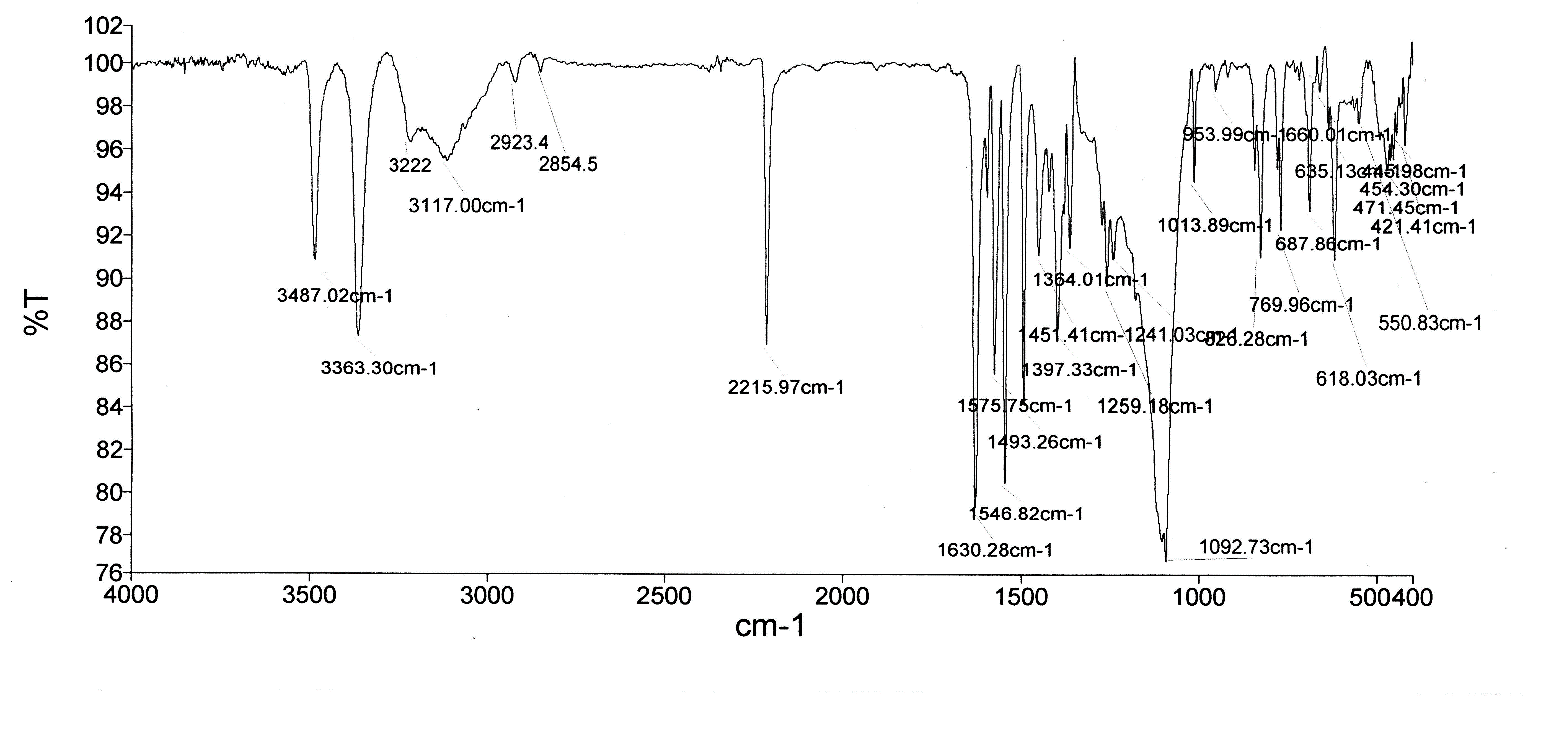


**Figure 42S.** ^1^H NMR spectrum of *5-amino-7-(4-nitrophenyl)-4,5-dihydro-[1,2,4]triazolo[1,5-a]pyrimidine-6-carbonitrile (8g)*

^
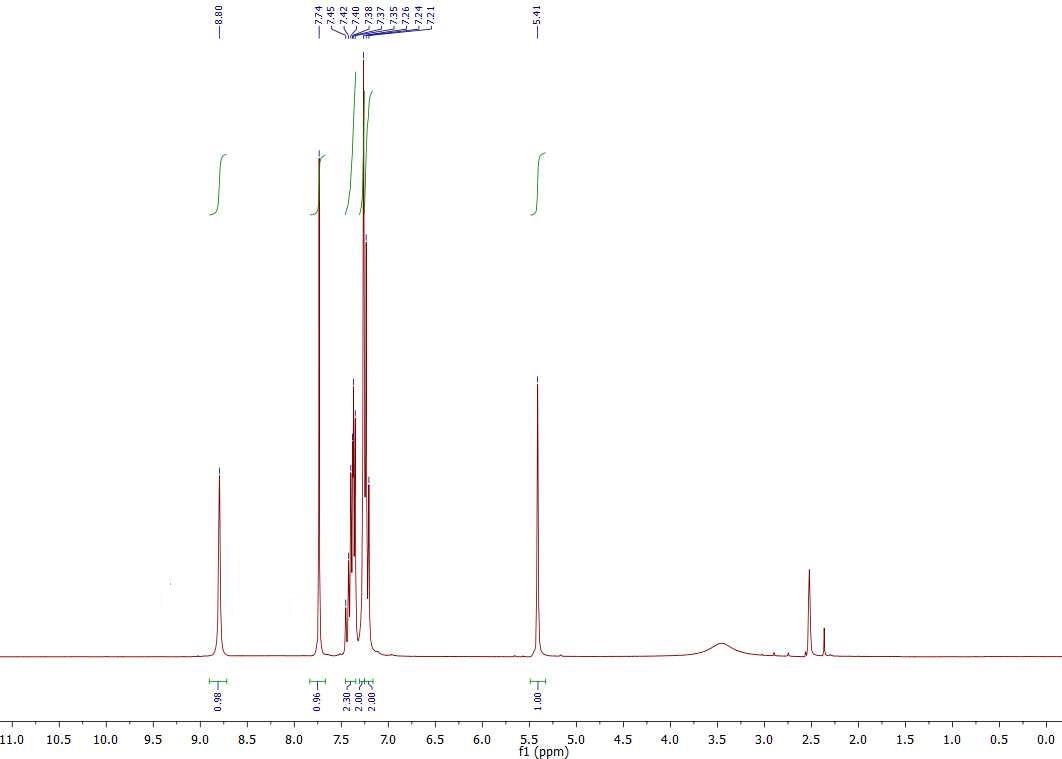
^

^
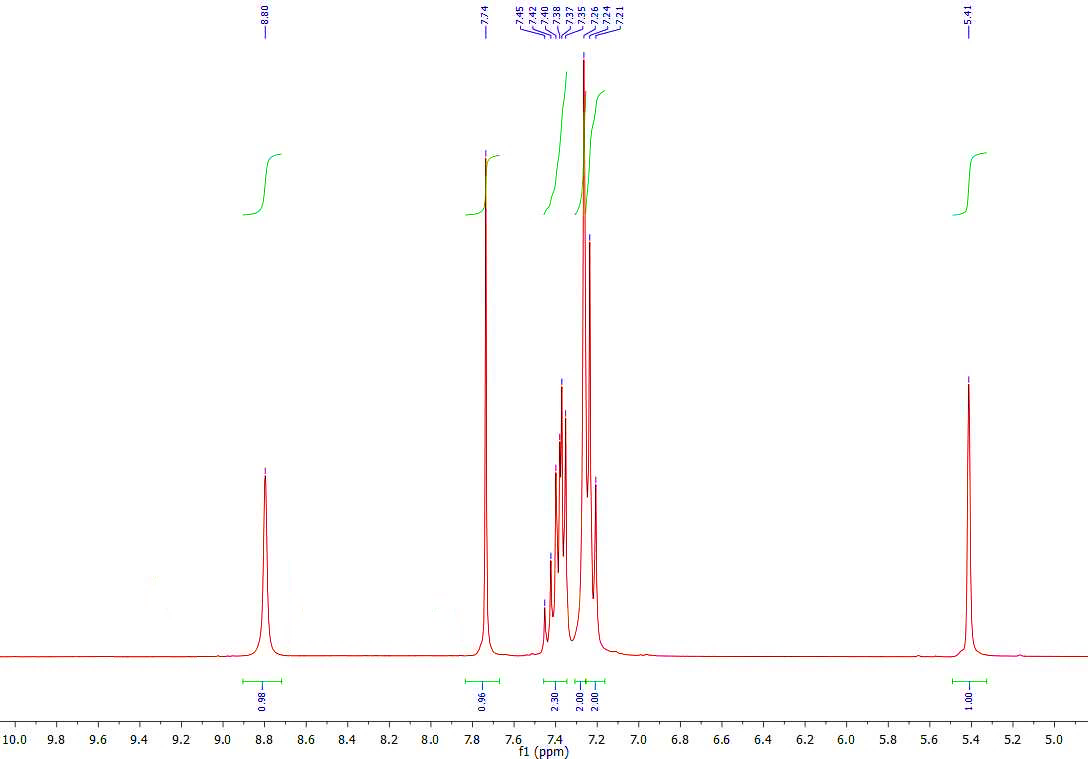
^

**Figure 43S.** ^13^C NMR spectrum of *5-amino-7-(4-nitrophenyl)-4,5-dihydro-[1,2,4]triazolo[1,5-a]pyrimidine-6-carbonitrile (8g)*

^^
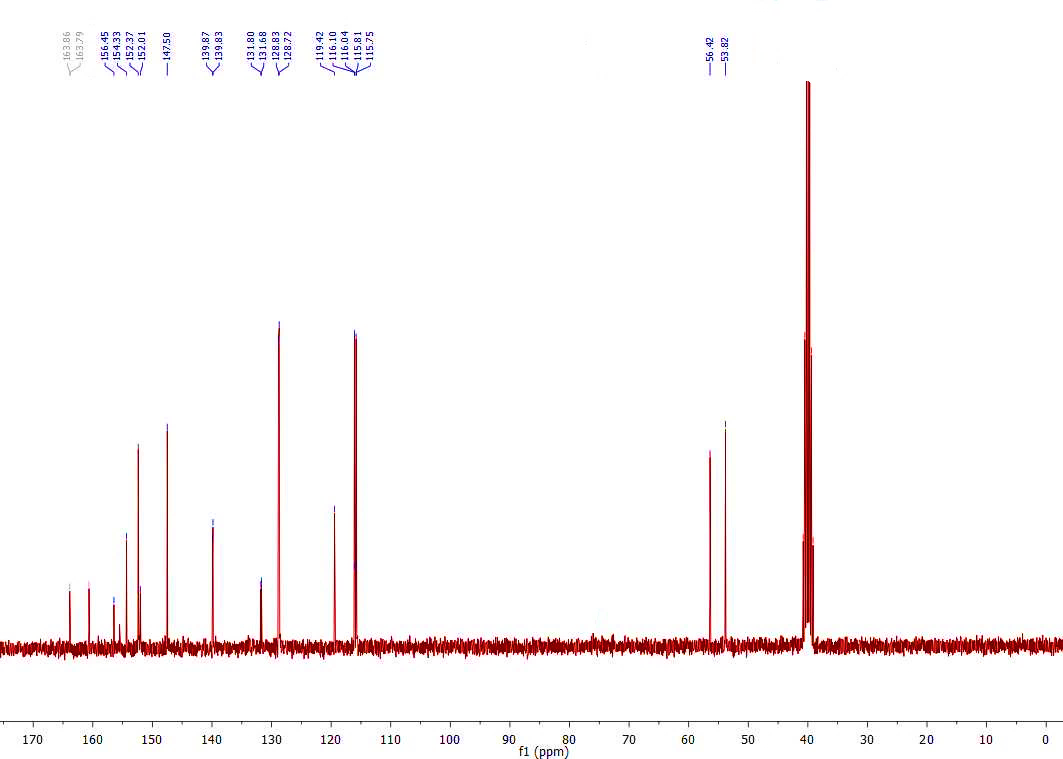


**Figure 44S.** FT-IR spectrum of *5-amino-7-(3,4-dimethoxyphenyl)-[1,2,4]triazolo[1,5-a]pyrimidine-6-carbonitrile (8'a)*


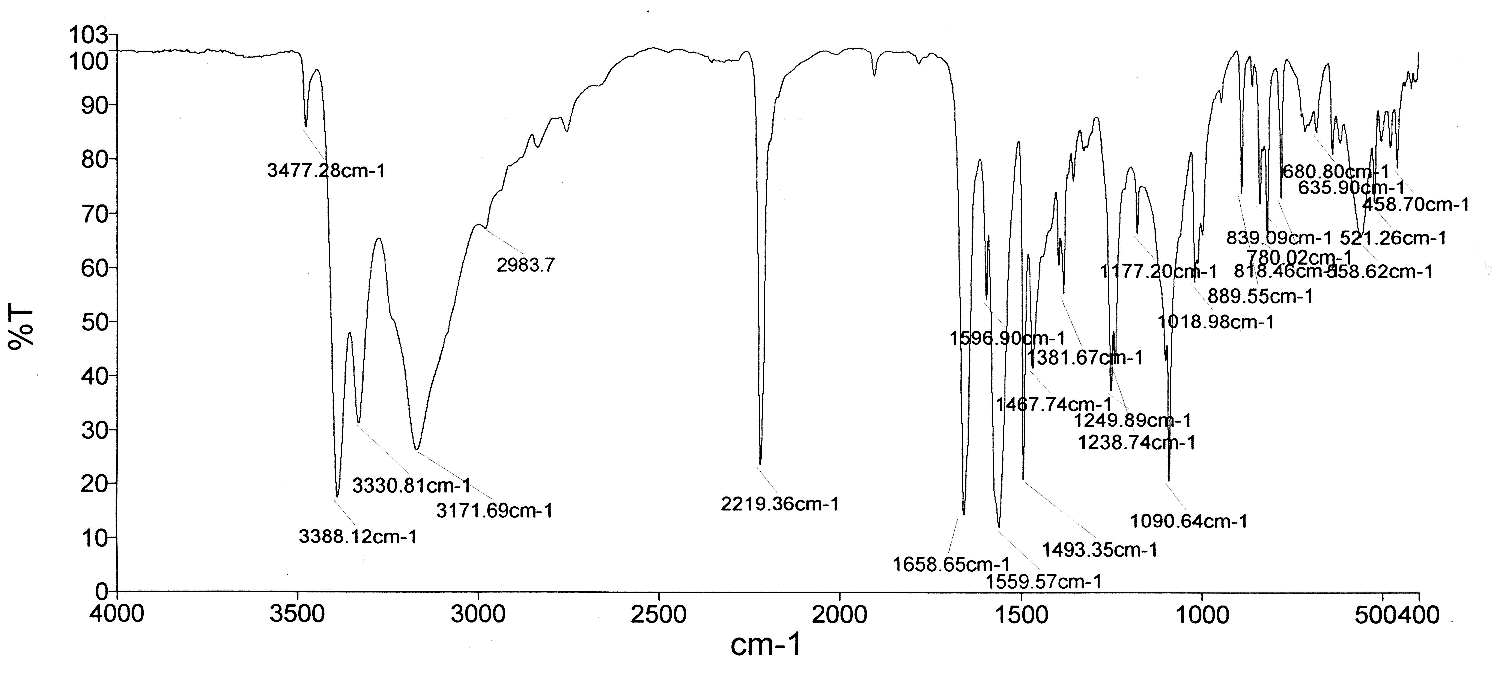


**Figure 45S.** ^1^H NMR spectrum of *5-amino-7-(3,4-dimethoxyphenyl)-[1,2,4]triazolo[1,5-a]pyrimidine-6-carbonitrile (8'a)*


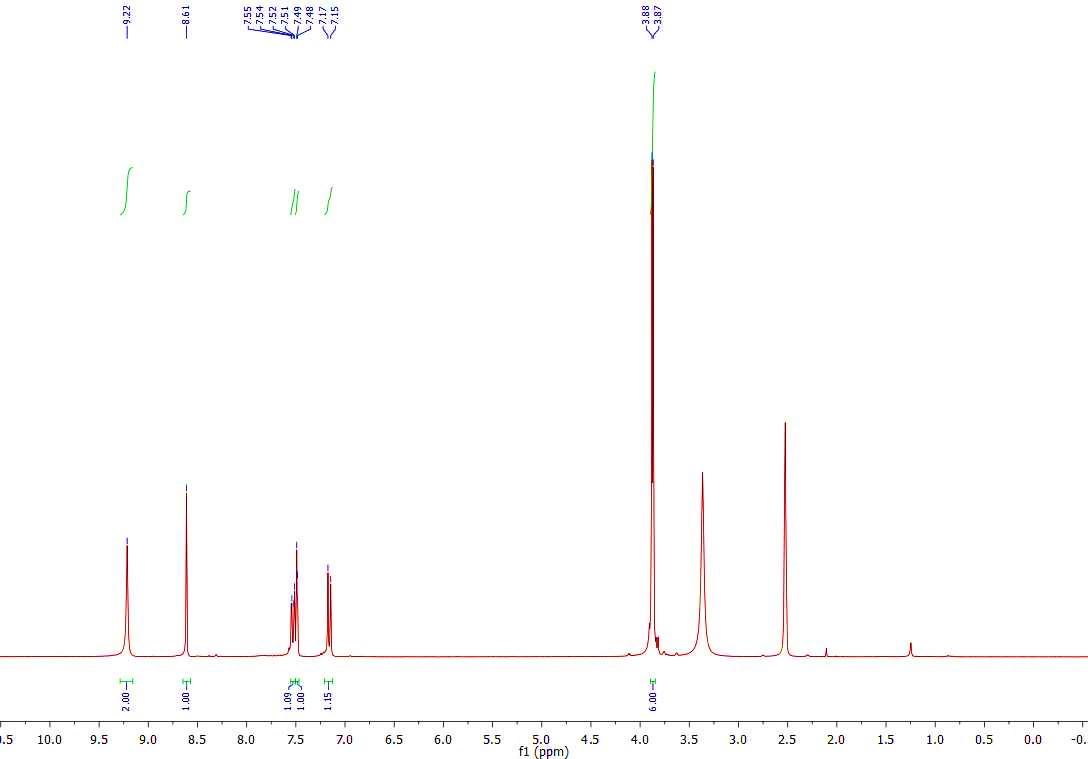


^
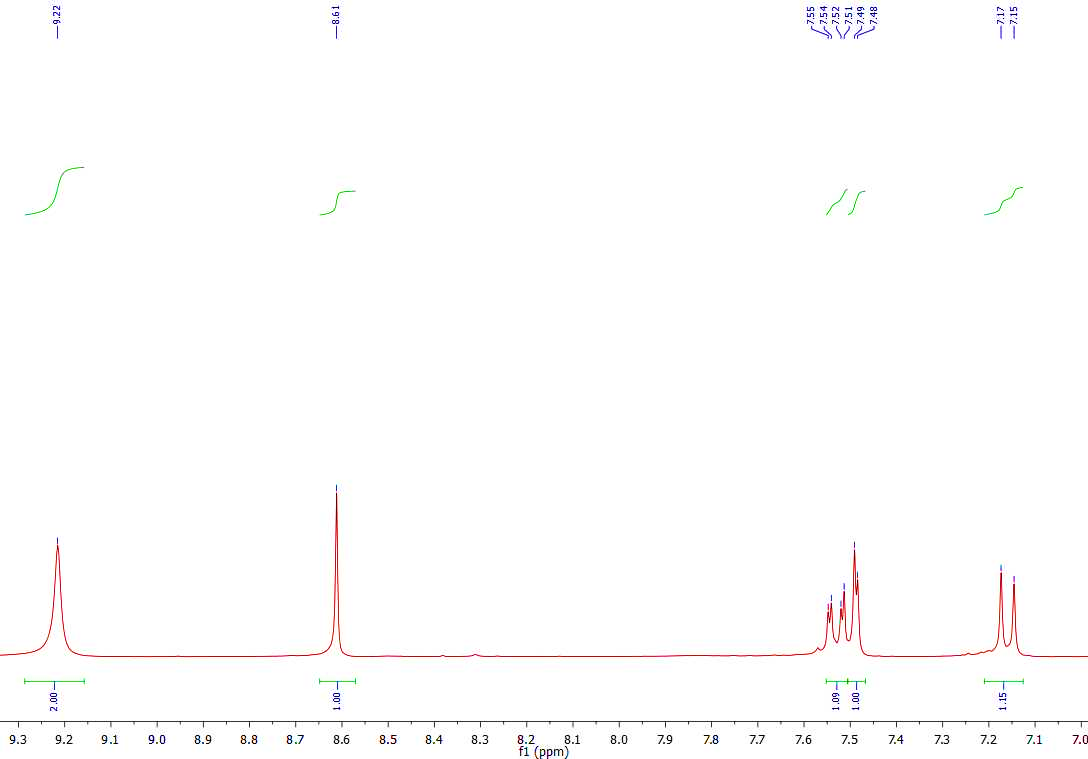
^

**Figure 46S.** ^13^C NMR spectrum of *5-amino-7-(3,4-dimethoxyphenyl)-[1,2,4]triazolo[1,5-a]pyrimidine-6-carbonitrile (8'a)*

^^
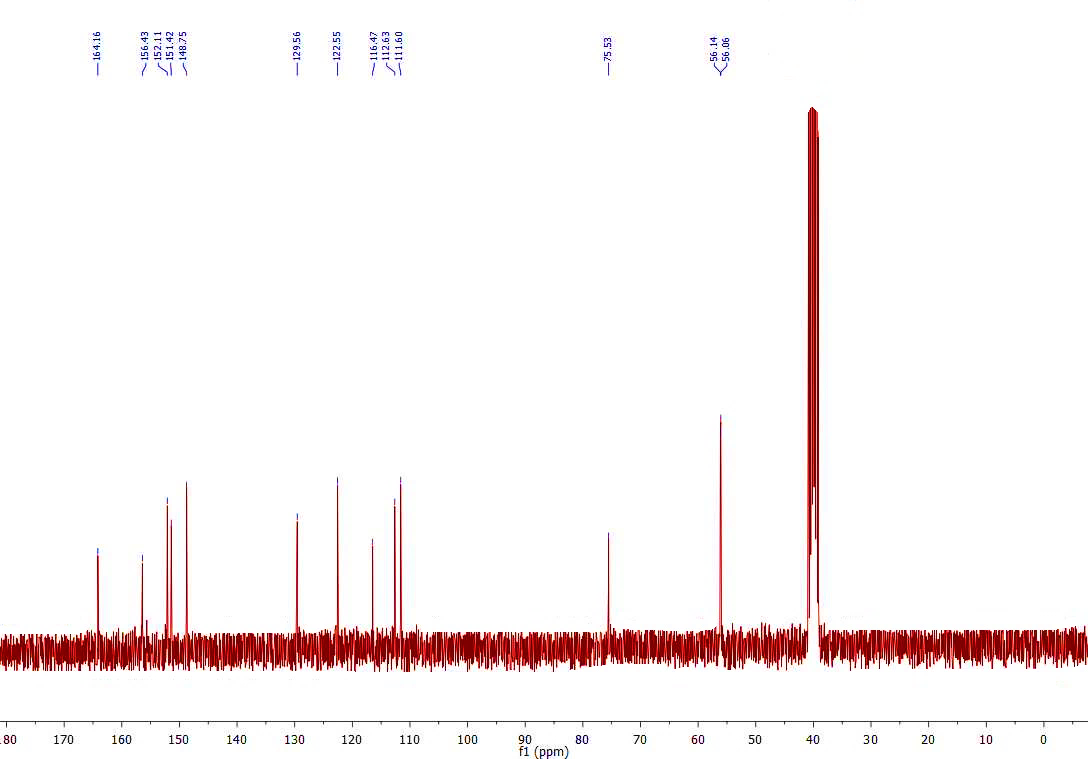


**Figure 47S.** HRMS spectrum of *5-amino-7-(3,4-dimethoxyphenyl)-[1,2,4]triazolo[1,5-a]pyrimidine-6-carbonitrile (8'a)*

**Figure 48S.** FT-IR spectrum of *5-amino-7-(4-hydroxy-3-methoxyphenyl)-[1,2,4]triazolo[1,5-a]pyrimidine-6-carbonitrile (8'b)*

**^
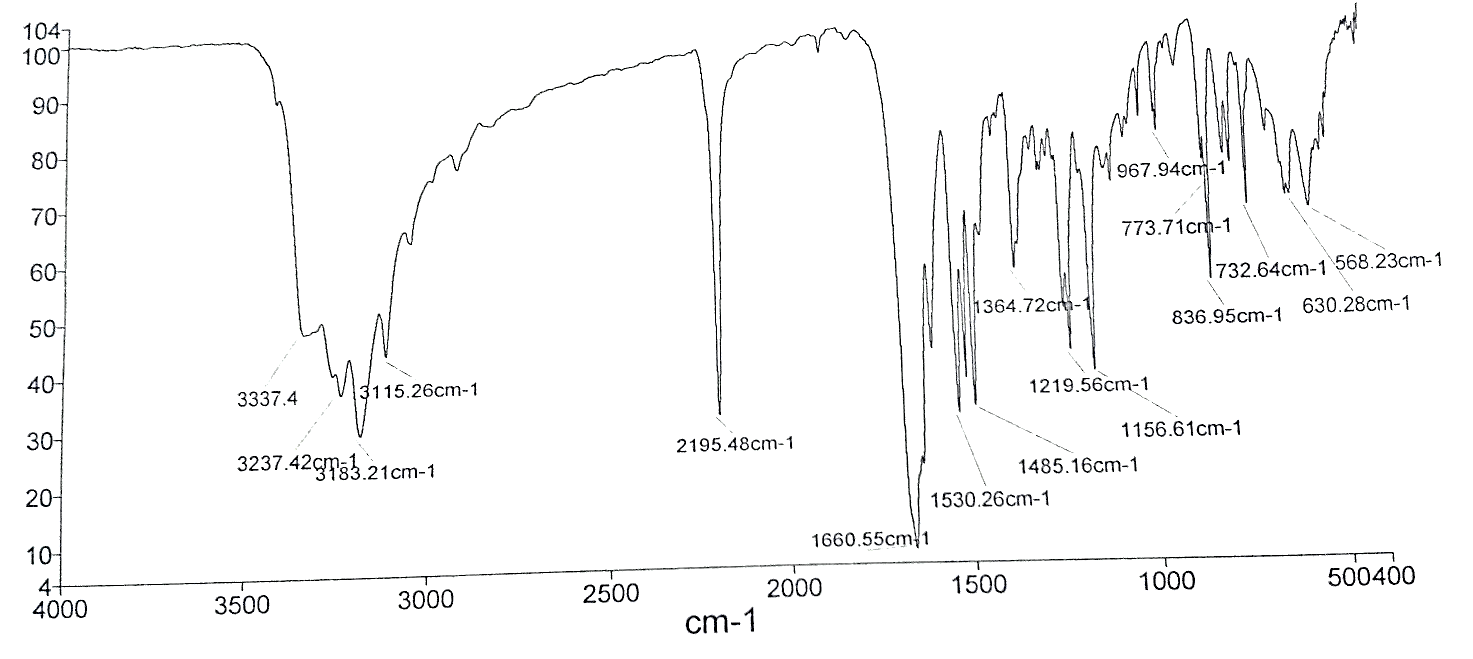
^**

**Figure 49S.** ^1^H NMR spectrum of *5-amino-7-(4-hydroxy-3-methoxyphenyl)-[1,2,4]triazolo[1,5-a]pyrimidine-6-carbonitrile (8'b)*


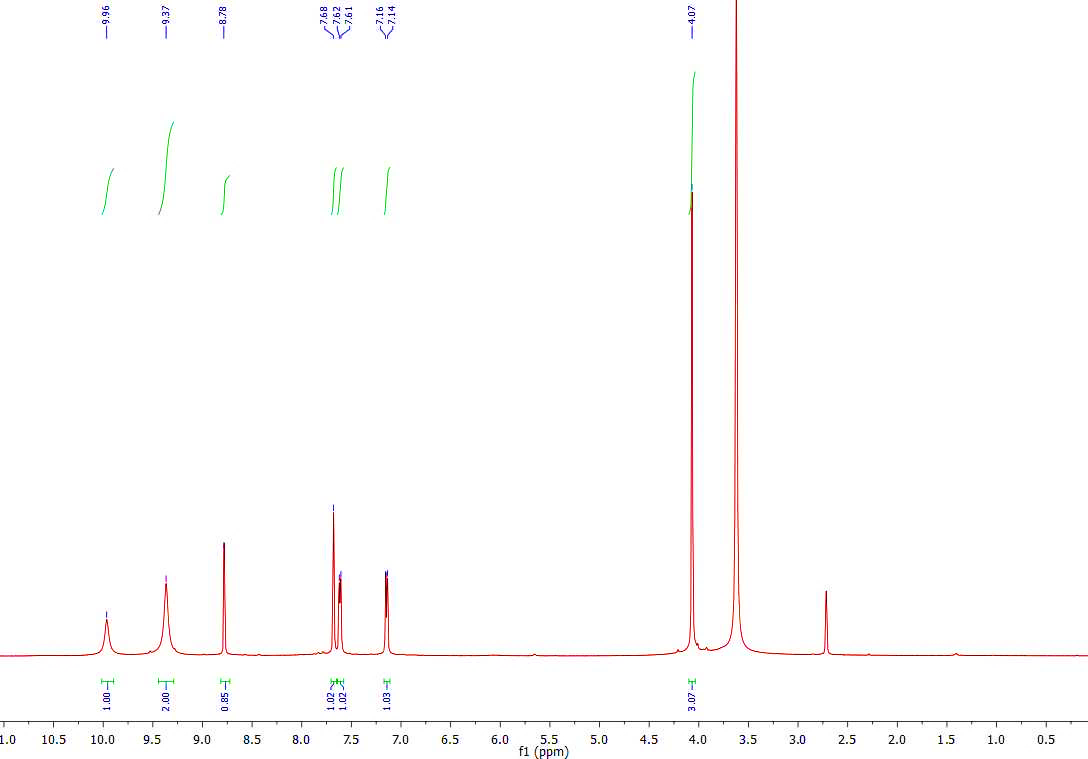


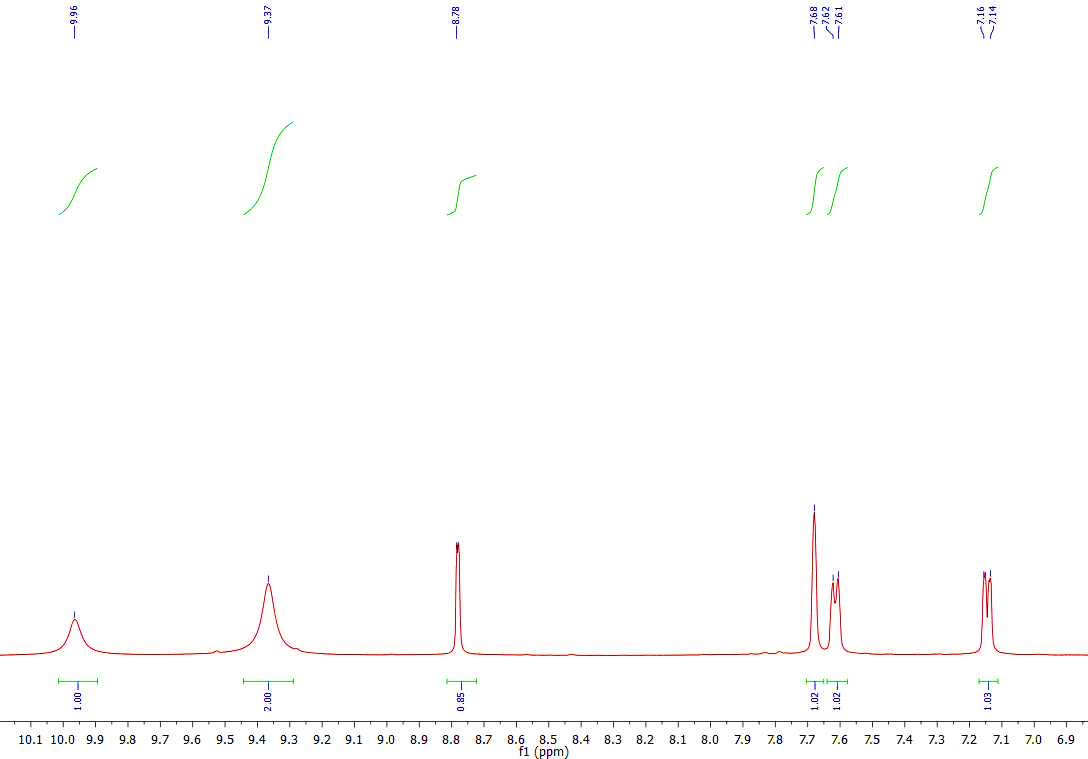


**Figure 50S.** ^13^C NMR spectrum of *5-amino-7-(4-hydroxy-3-methoxyphenyl)-[1,2,4]triazolo[1,5-a]pyrimidine-6-carbonitrile (8'b)*


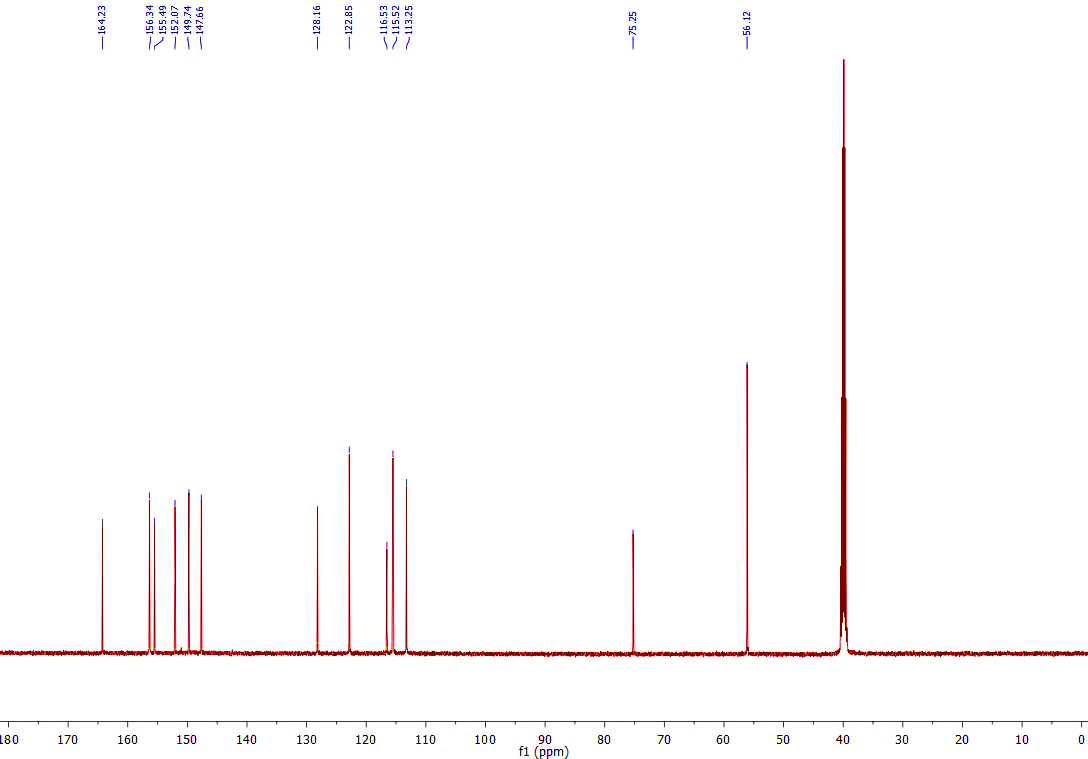


**Figure 51S.** HRMS of *5-amino-7-(4-hydroxy-3-methoxyphenyl)-[1,2,4]triazolo[1,5-a]pyrimidine-6-carbonitrile (8'b)*

**Figure 52S.** FT-IR spectrum of *5-amino-7-(4-hydroxyphenyl)-[1,2,4]triazolo[1,5-a]pyrimidine-6-carbonitrile (8'c)*

**^
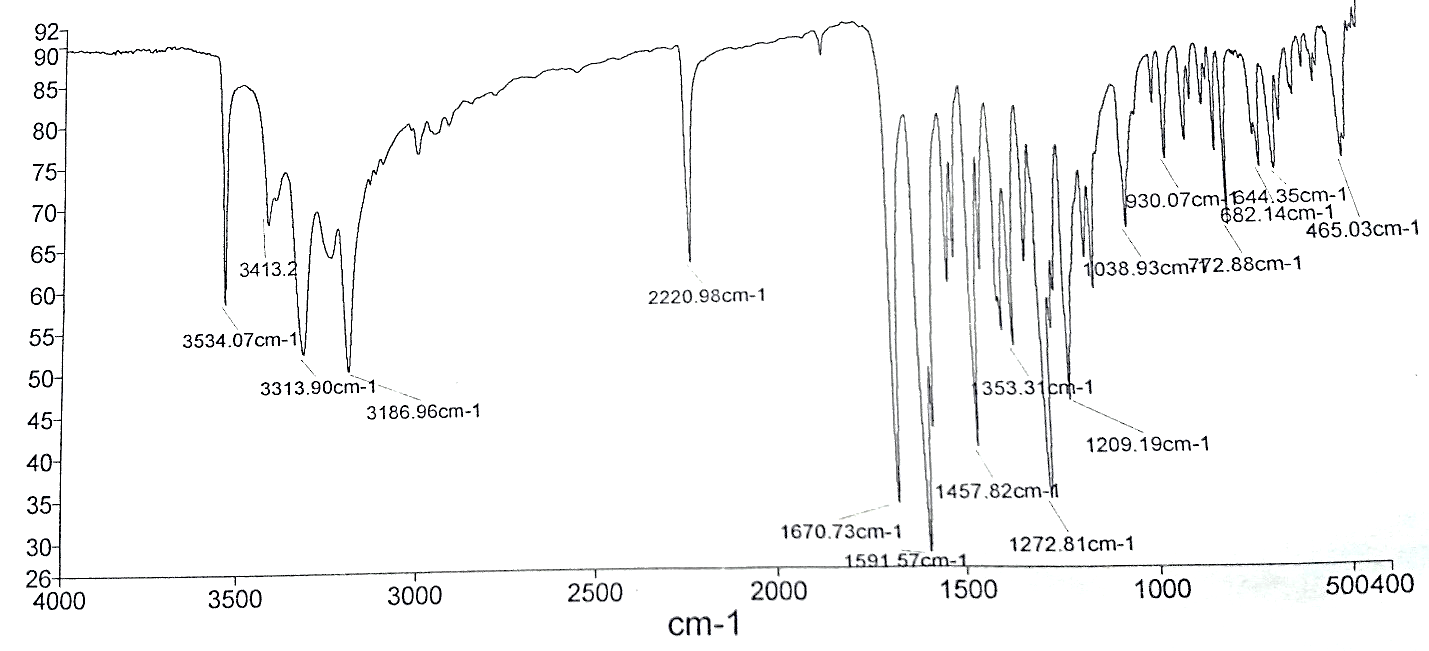
^**

**Figure 53S.** ^1^H NMR spectrum of *5-amino-7-(4-hydroxyphenyl)-[1,2,4]triazolo[1,5-a]pyrimidine-6-carbonitrile (8'c)*


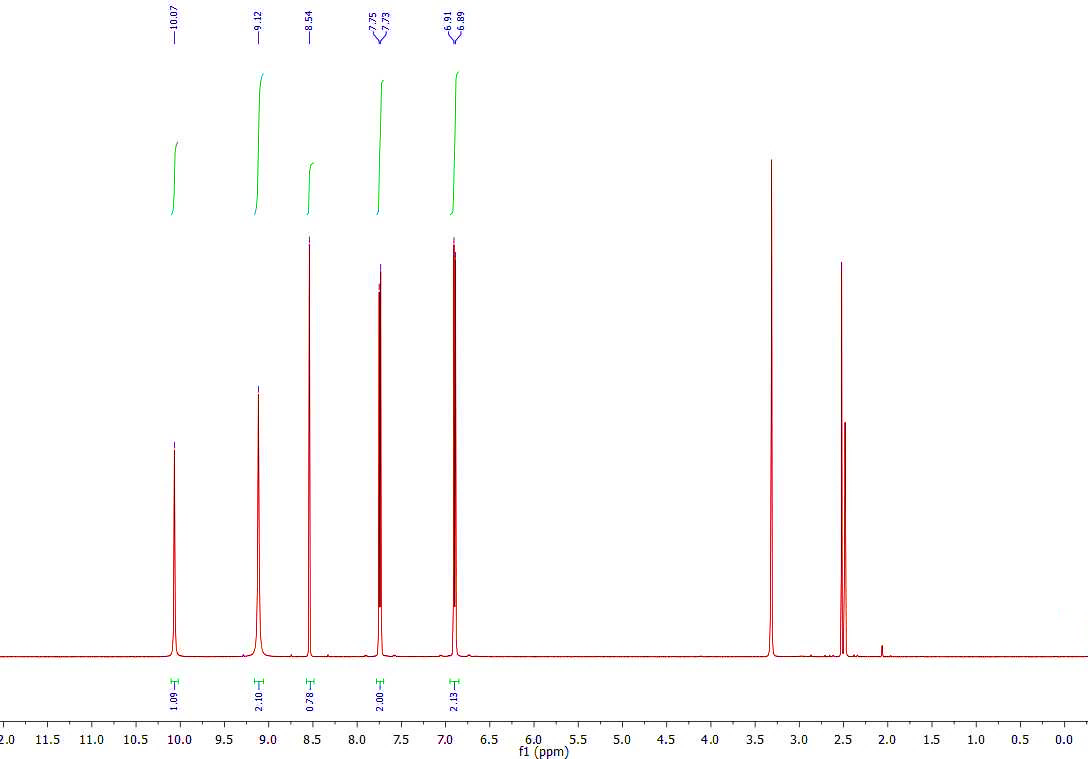


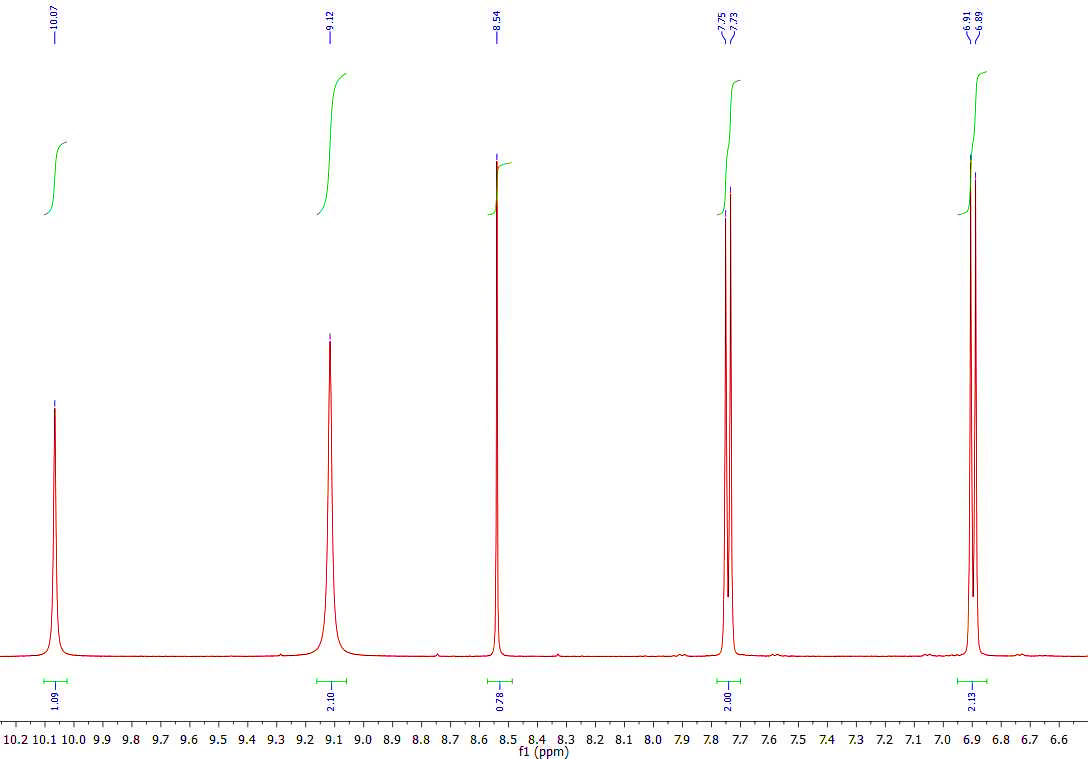


**Figure 54S.** ^13^C NMR spectrum of *5-amino-7-(4-hydroxyphenyl)-[1,2,4]triazolo[1,5-a]pyrimidine-6-carbonitrile (8'c)*


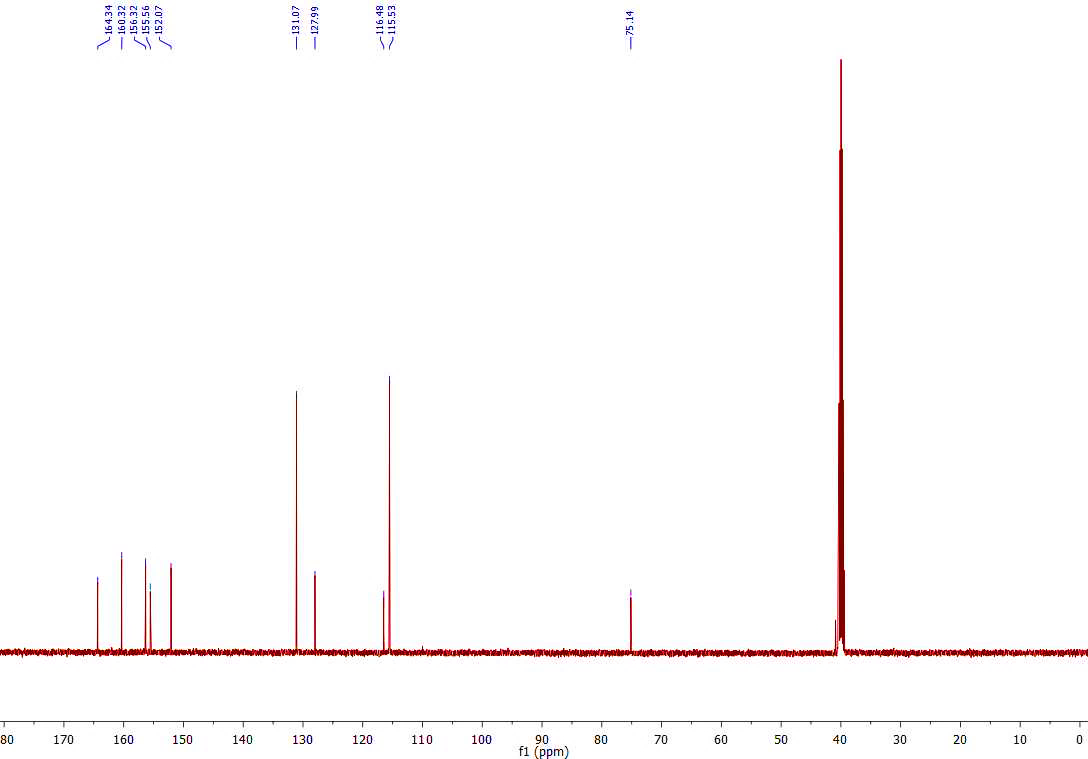


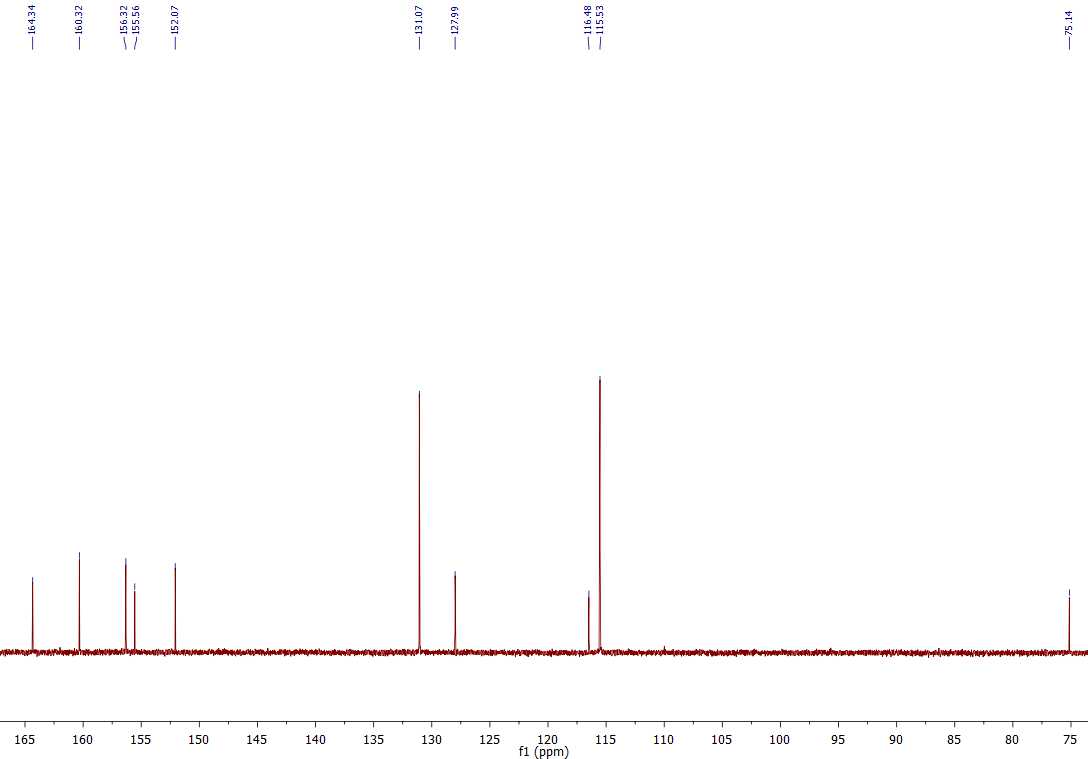


**Figure 55S.** HRMS of *5-amino-7-(4-hydroxyphenyl)-[1,2,4]triazolo[1,5-a]pyrimidine-6-carbonitrile (8'c)*

**Figure 56S.** FT-IR spectrum of *5-amino-7-(2-bromophenyl)-[1,2,4]triazolo[1,5-a]pyrimidine-6-carbonitrile (8'd)*

**^
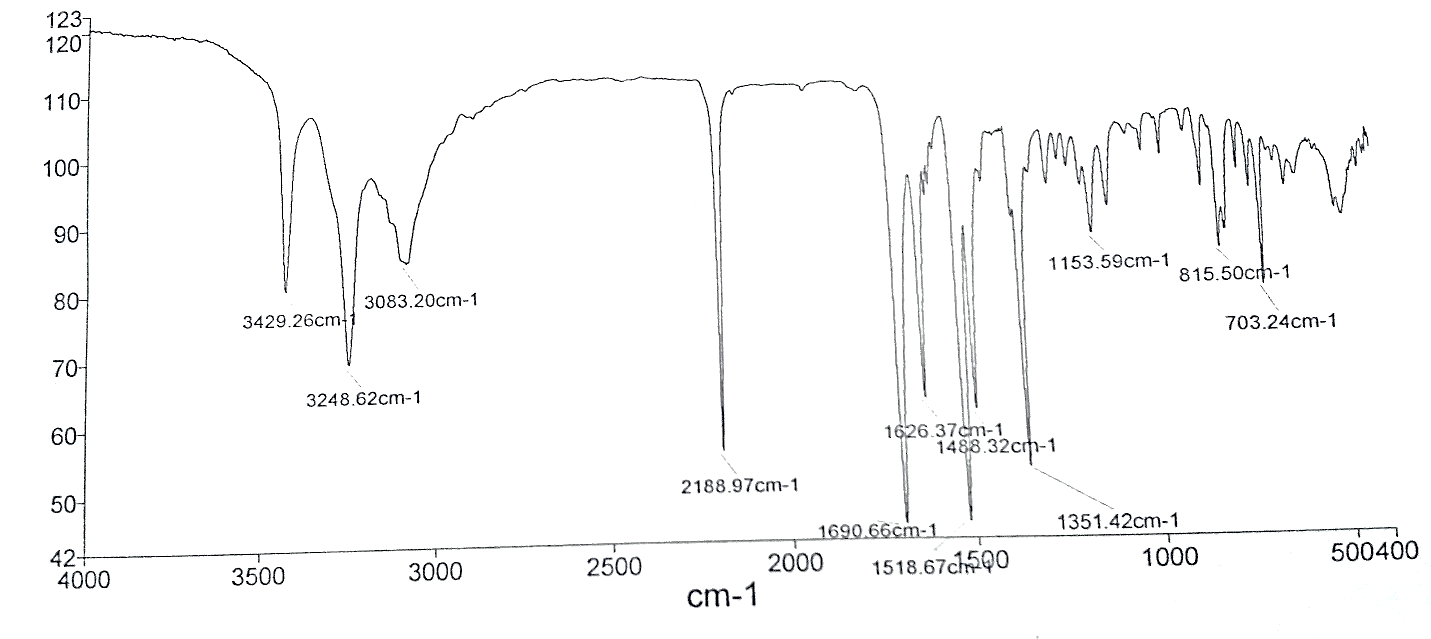
^**

**Figure 57S.** ^1^H NMR spectrum of *5-amino-7-(2-bromophenyl)-[1,2,4]triazolo[1,5-a]pyrimidine-6-carbonitrile (8'd)*


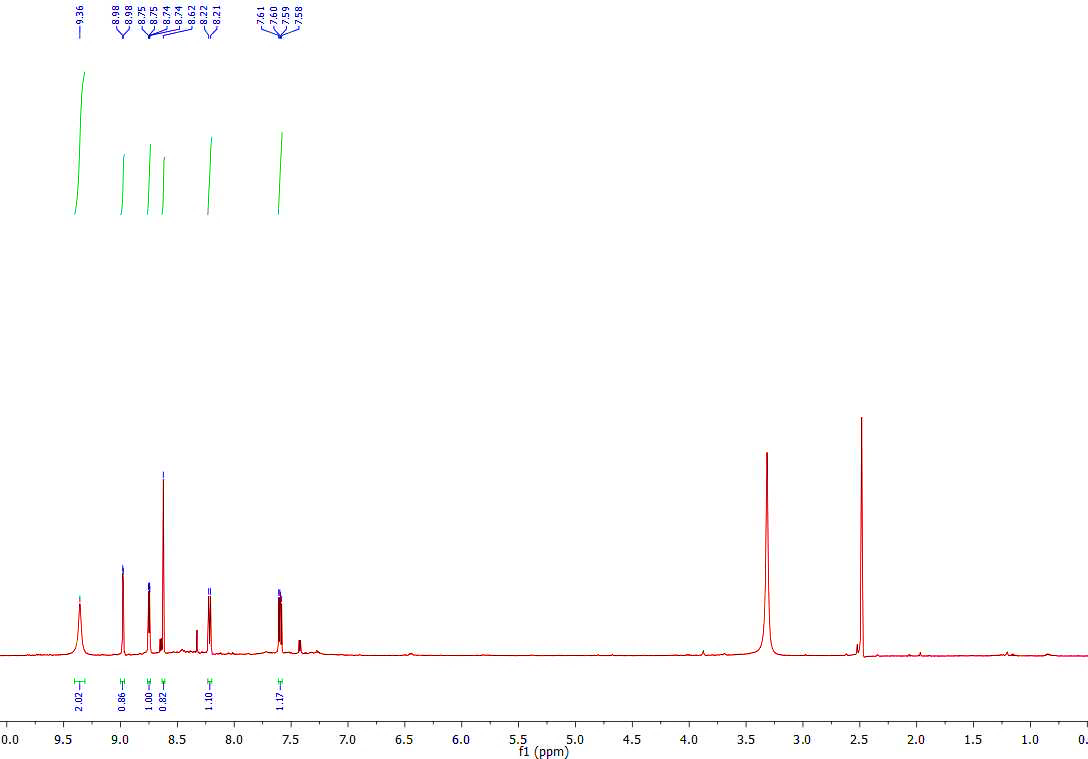


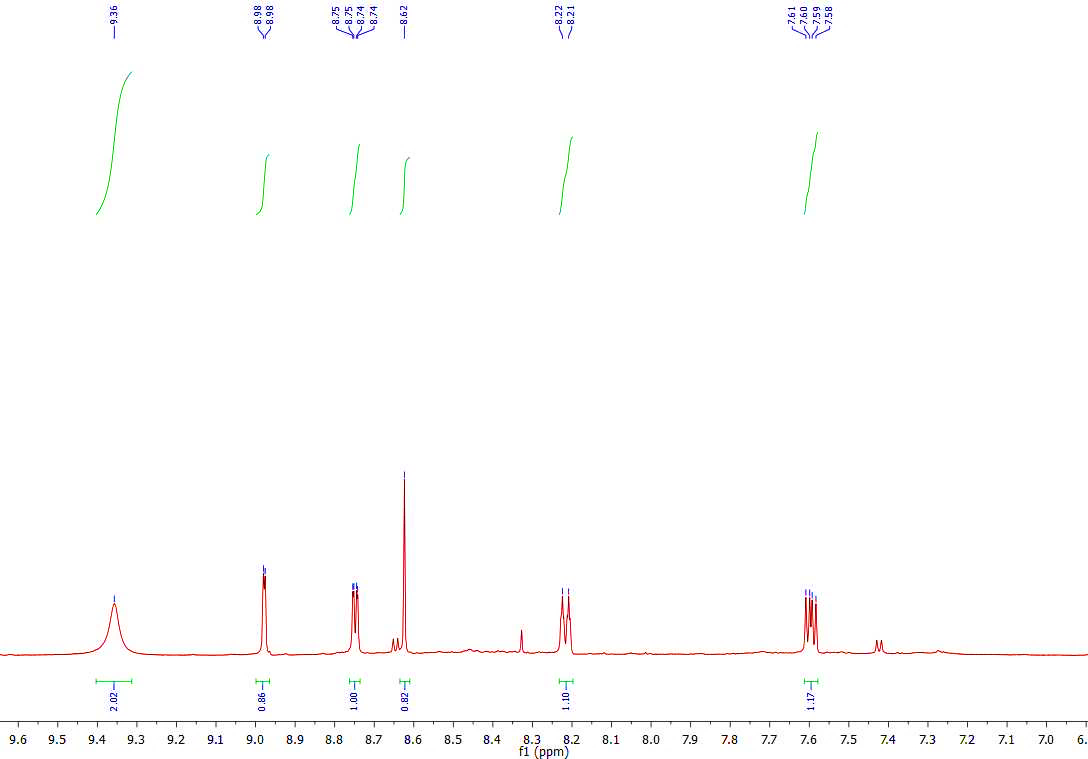


**Figure 58S.** ^13^C NMR spectrum of *5-amino-7-(2-bromophenyl)-[1,2,4]triazolo[1,5-a]pyrimidine-6-carbonitrile (8'd)*


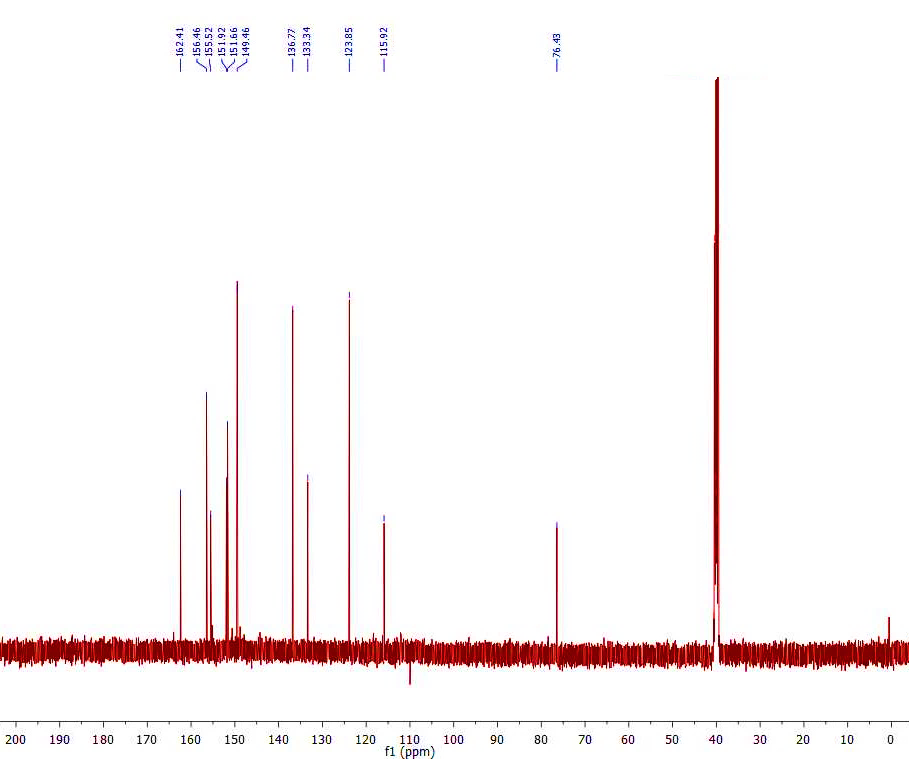


**Figure 59S.** HRMS spectrum of *5-amino-7-(2-bromophenyl)-[1,2,4]triazolo[1,5-a]pyrimidine-6-carbonitrile (8'd)*

**Figure 60S.** FT-IR spectrum of *5-amino-7-(2-hydroxy-3-methoxyphenyl)-[1,2,4]triazolo[1,5-a]pyrimidine-6-carbonitrile (8'e)*

**^
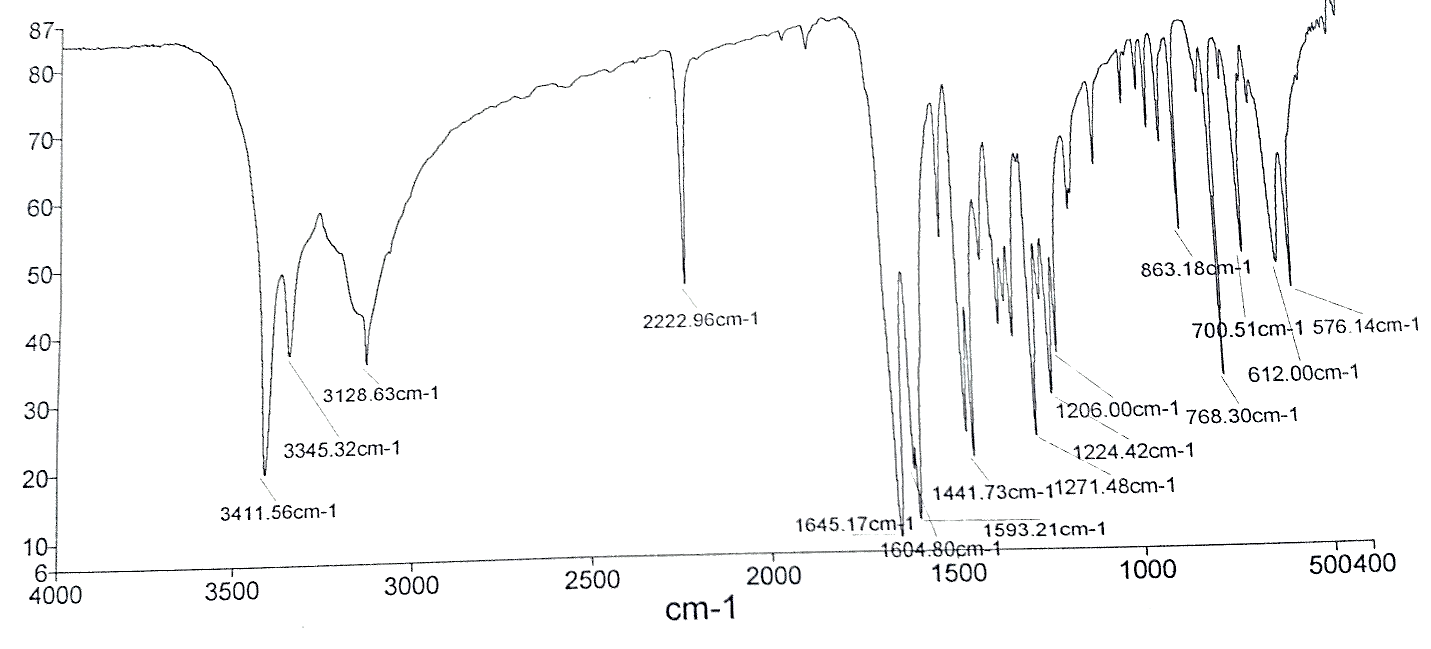
^**

**Figure 61S.** ^1^H NMR spectrum of *5-amino-7-(2-hydroxy-3-methoxyphenyl)-[1,2,4]triazolo[1,5-a]pyrimidine-6-carbonitrile (8'e)*


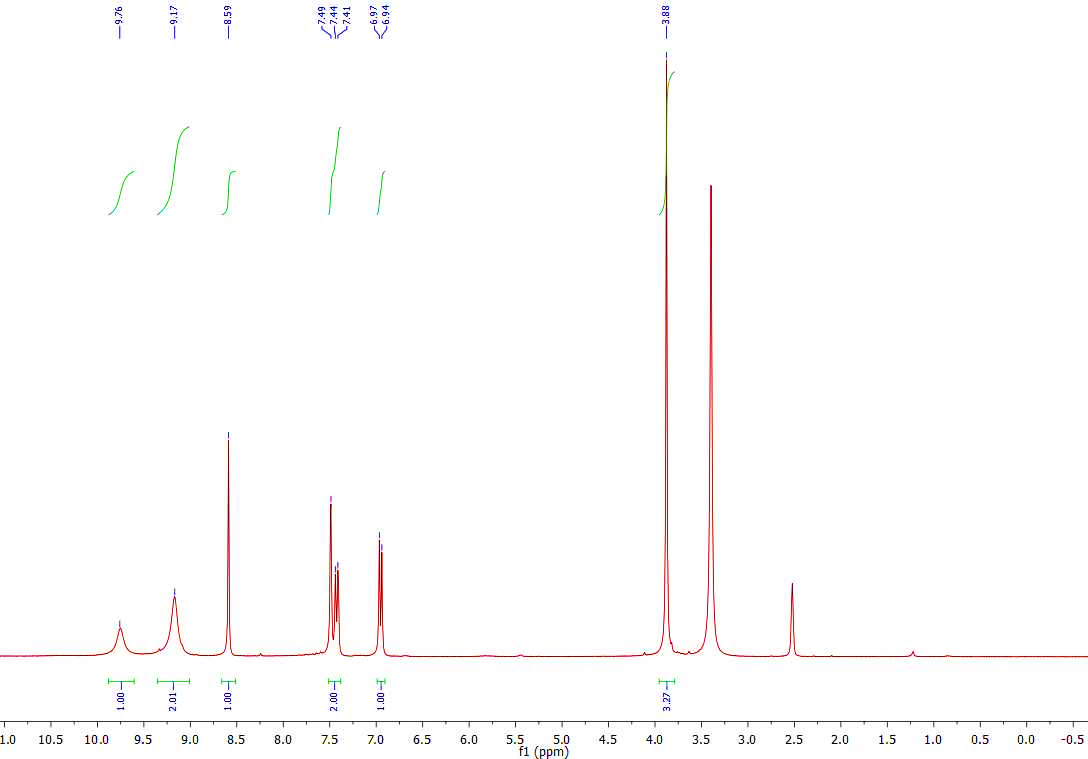

^
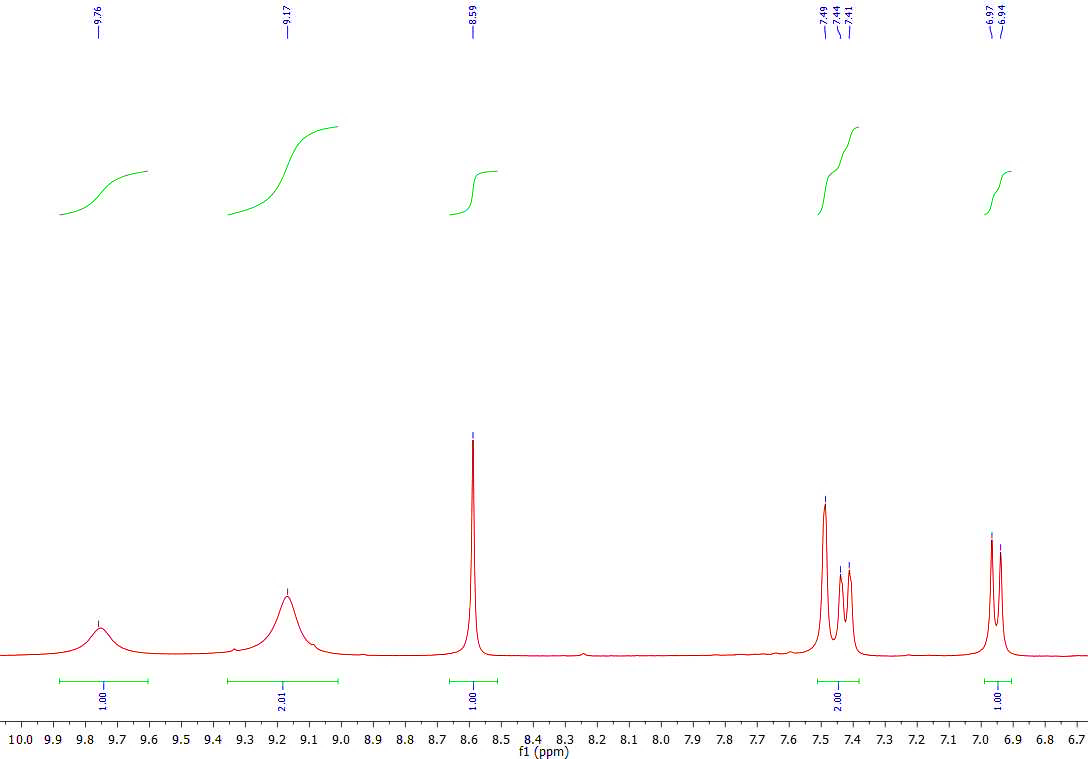
^

**Figure 62S.** ^13^C NMR spectrum of *5-amino-7-(2-hydroxy-3-methoxyphenyl)-[1,2,4]triazolo[1,5-a]pyrimidine-6-carbonitrile (8'e)*


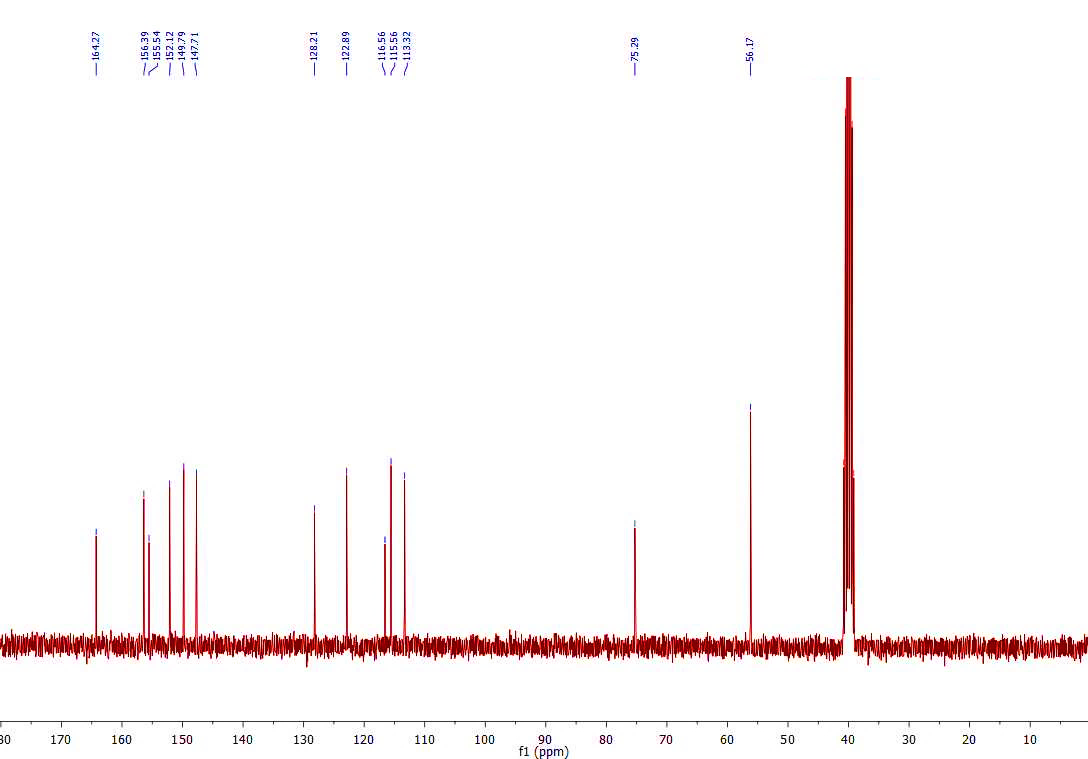


**Figure 63S.** FT-IR spectrum of *5-amino-7-(4-methoxyphenyl)-[1,2,4]triazolo[1,5-a]pyrimidine-6-carbonitrile (8'f)*


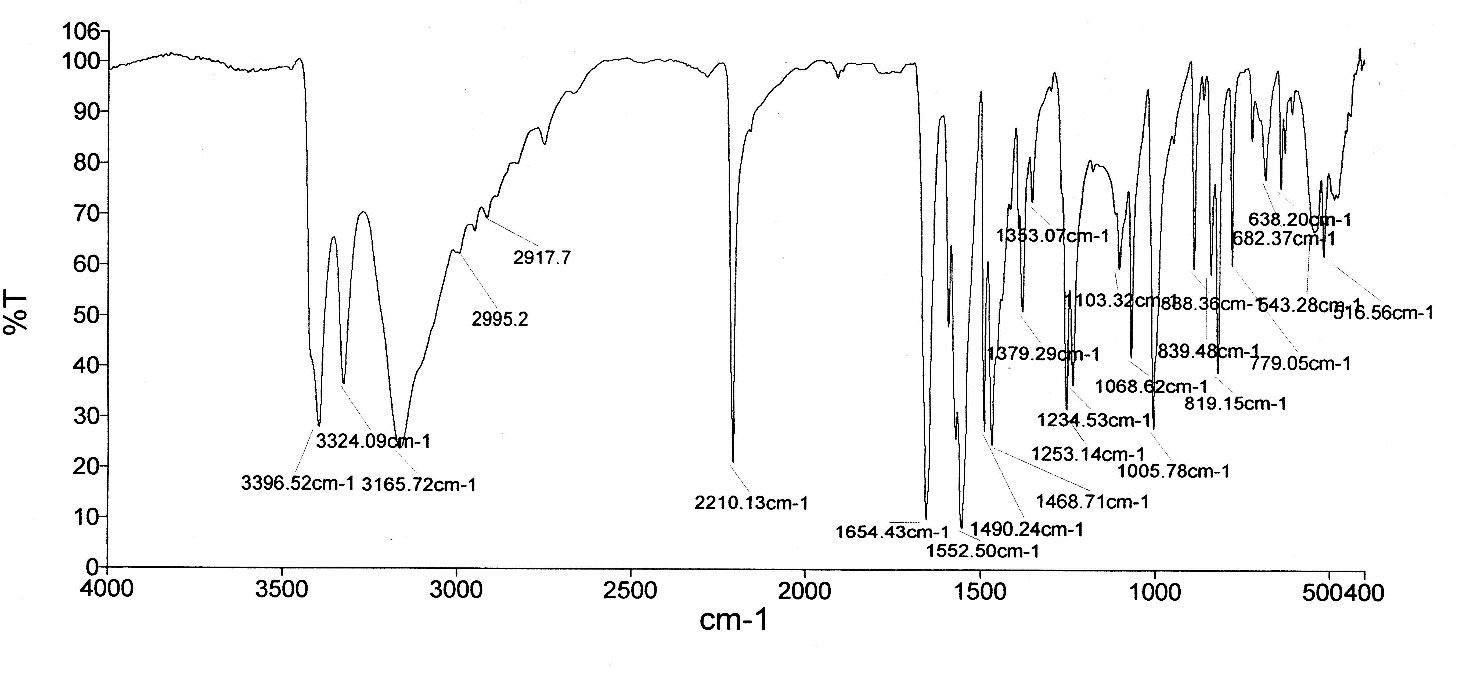


**Figure 64S.** ^1^H NMR spectrum of *5-amino-7-(4-methoxyphenyl)-[1,2,4]triazolo[1,5-a]pyrimidine-6-carbonitrile (8'f)*


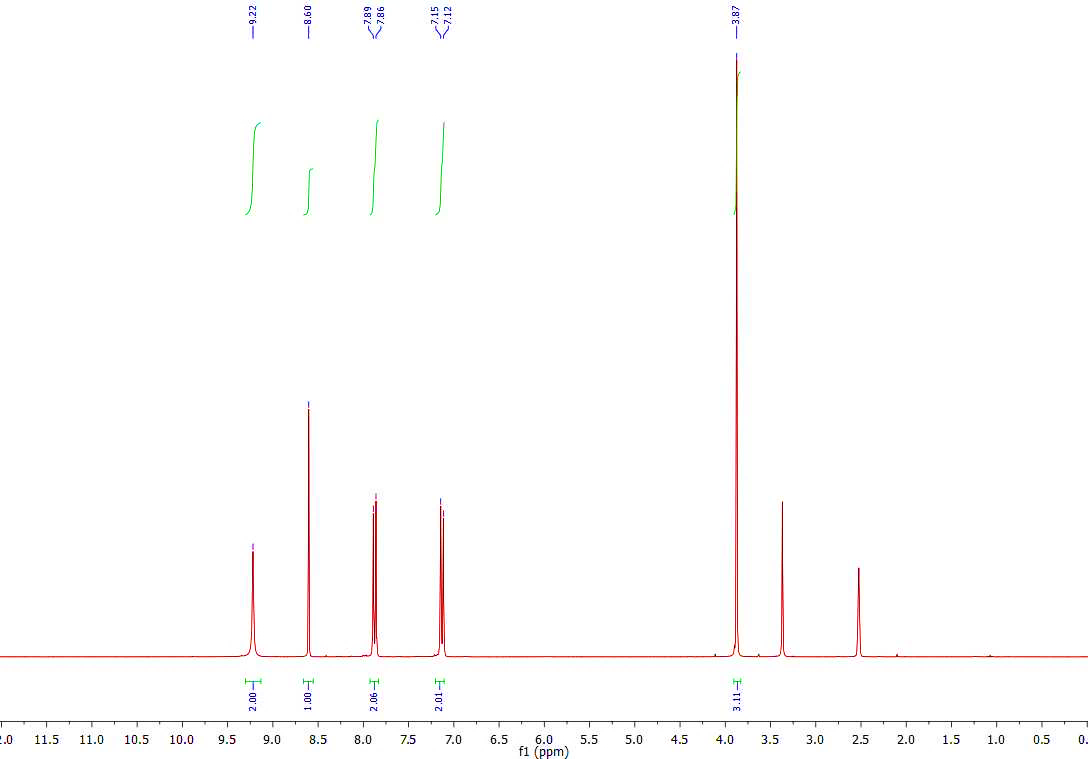


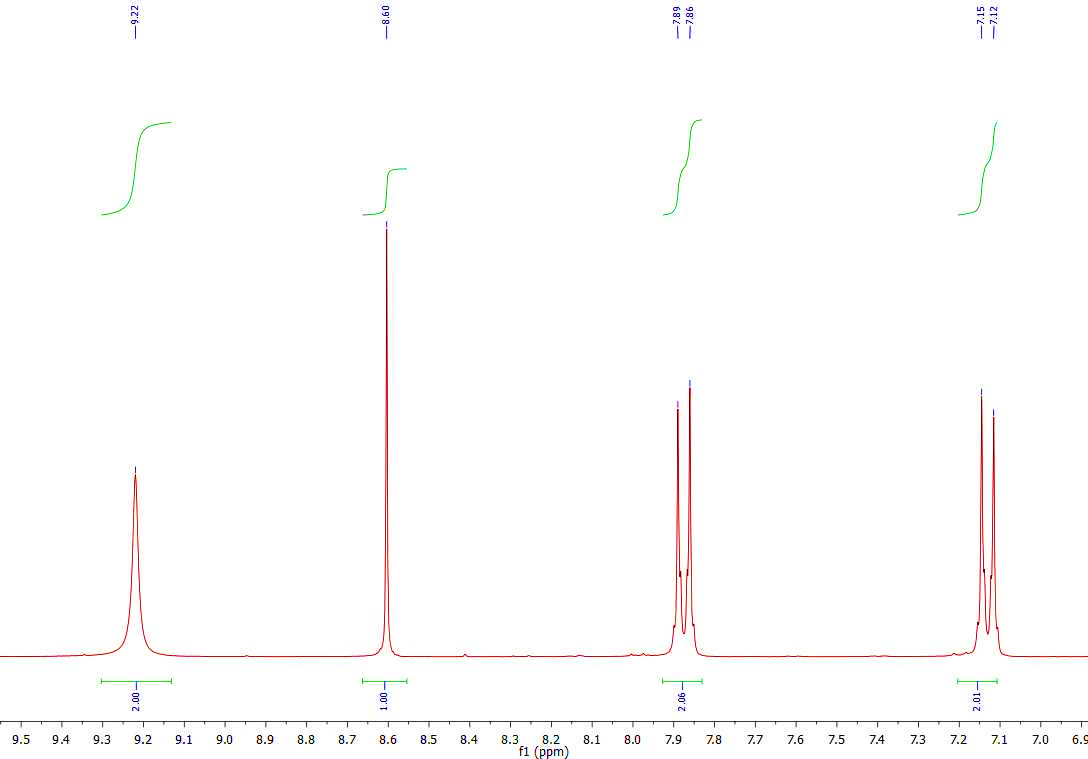


**Figure 65S.** ^13^C NMR spectrum of *5-amino-7-(4-methoxyphenyl)-[1,2,4]triazolo[1,5-a]pyrimidine-6-carbonitrile (8'f)*


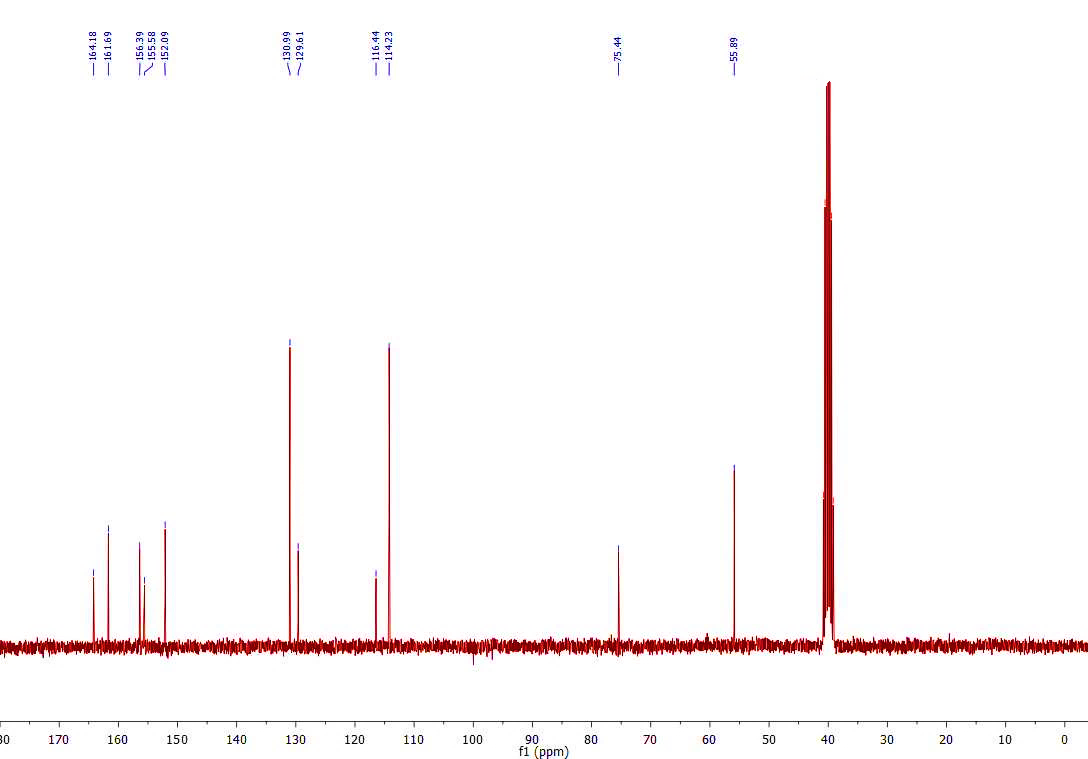


**Figure 66S.** FT-IR spectrum of *5-amino-7-(3-ethoxy-4-hydroxyphenyl)-[1,2,4]triazolo[1,5-a]pyrimidine-6-carbonitrile (8'g)*

**^
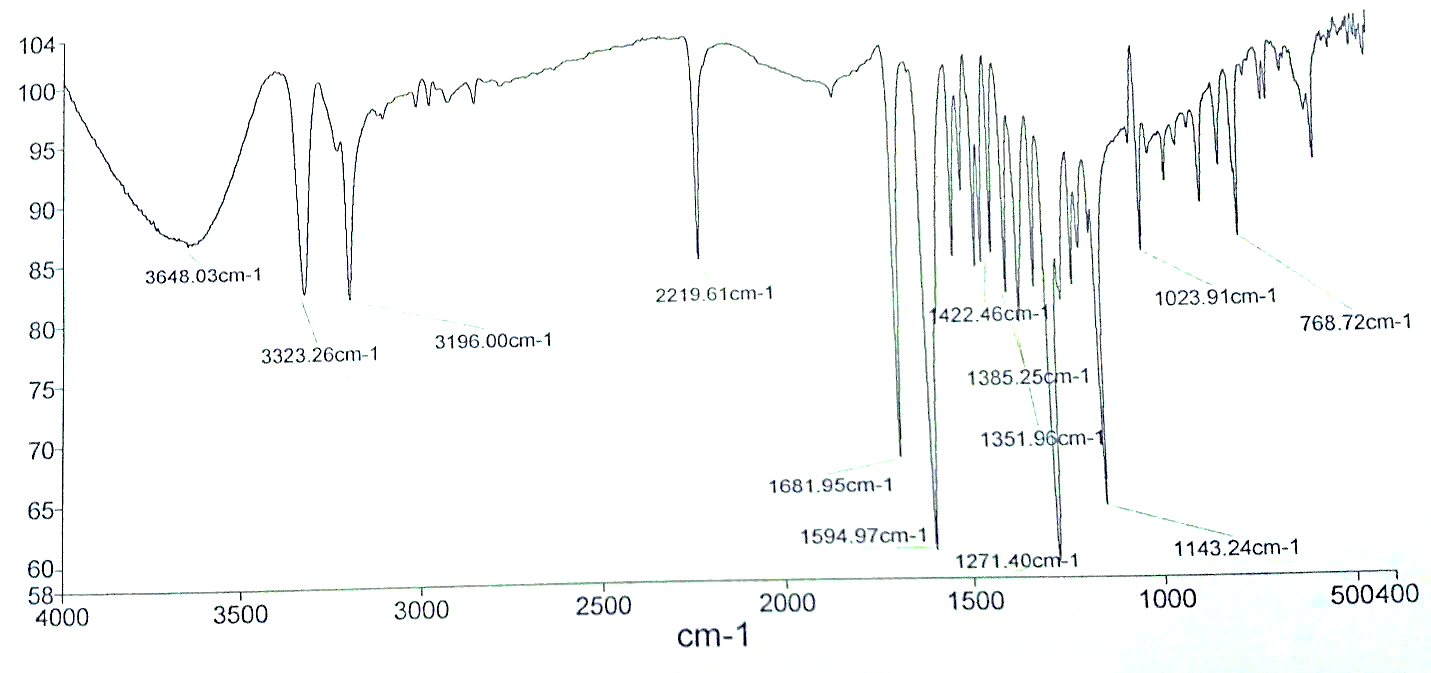
^**

**Figure 67S.** ^1^H NMR spectrum of *5-amino-7-(3-ethoxy-4-hydroxyphenyl)-[1,2,4]triazolo[1,5-a]pyrimidine-6-carbonitrile (8'g)*


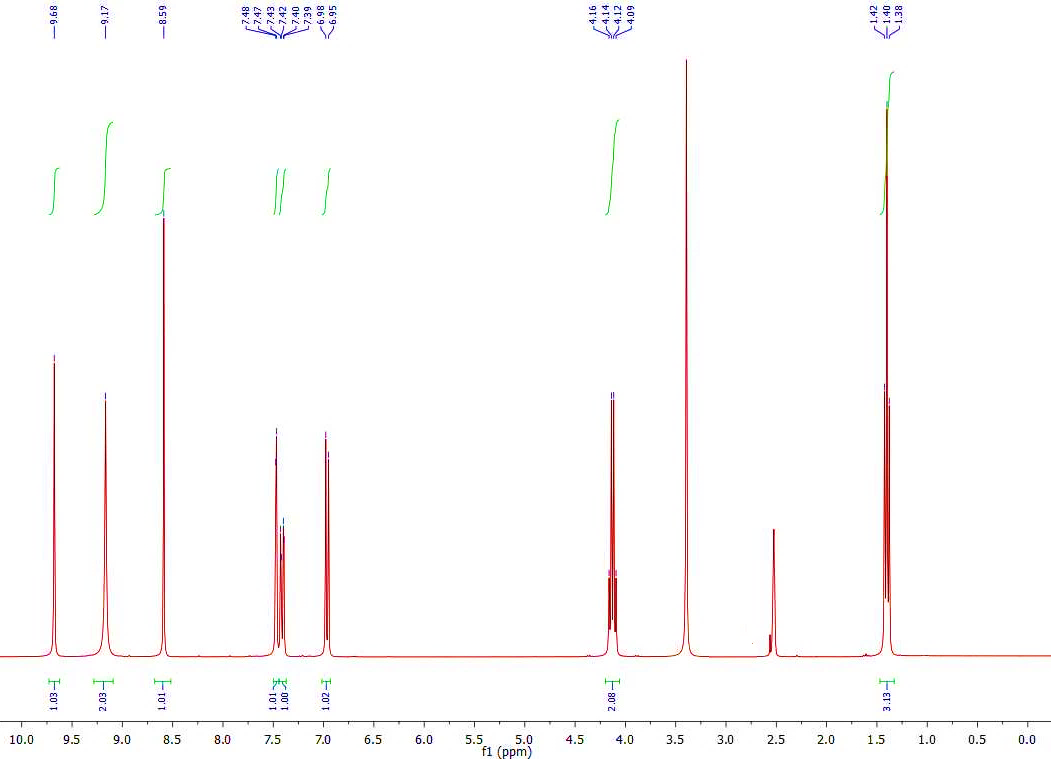


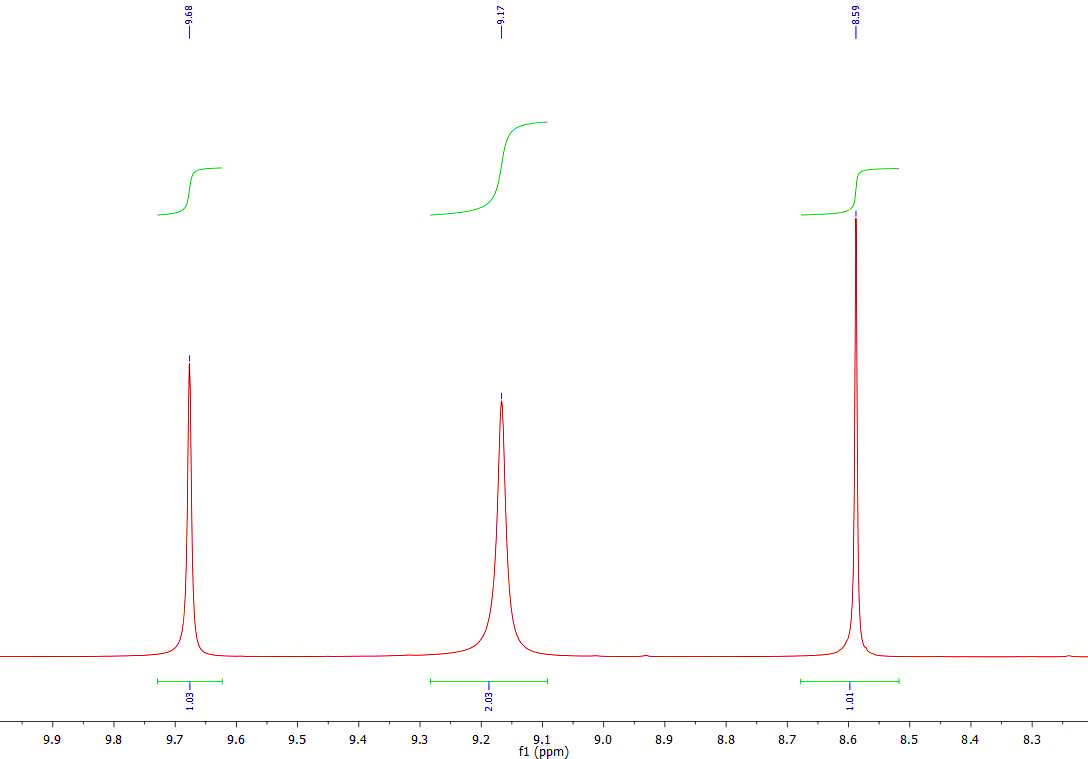


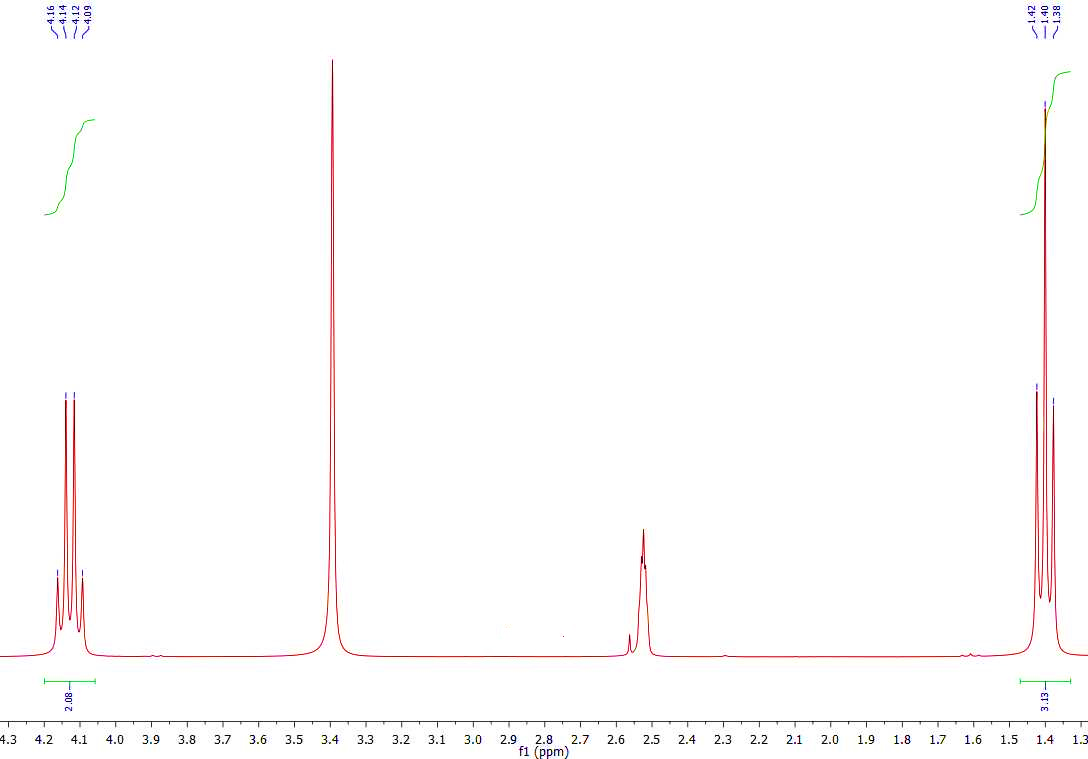


**Figure 68S.** ^13^C NMR spectrum of *5-amino-7-(3-ethoxy-4-hydroxyphenyl)-[1,2,4]triazolo[1,5-a]pyrimidine-6-carbonitrile (8'g)*


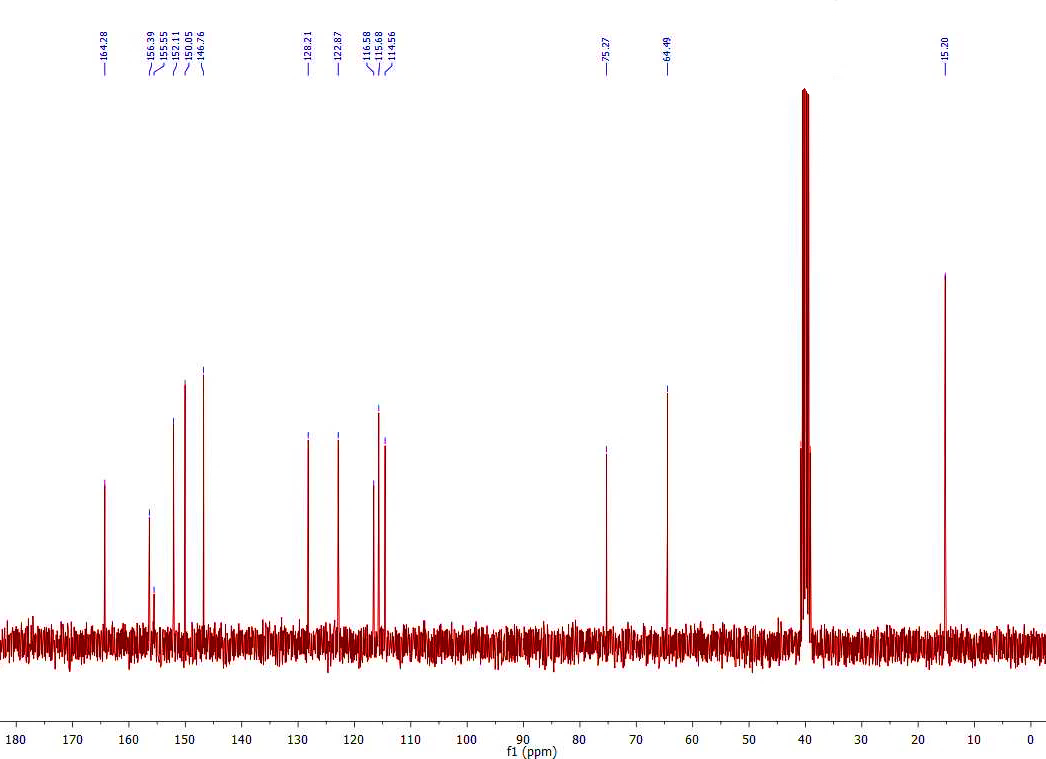


**Figure 69S.** FT-IR spectrum of *5-amino-7-(3-hydroxyphenyl)-[1,2,4]triazolo[1,5-a]pyrimidine-6-carbonitrile (8'h)*


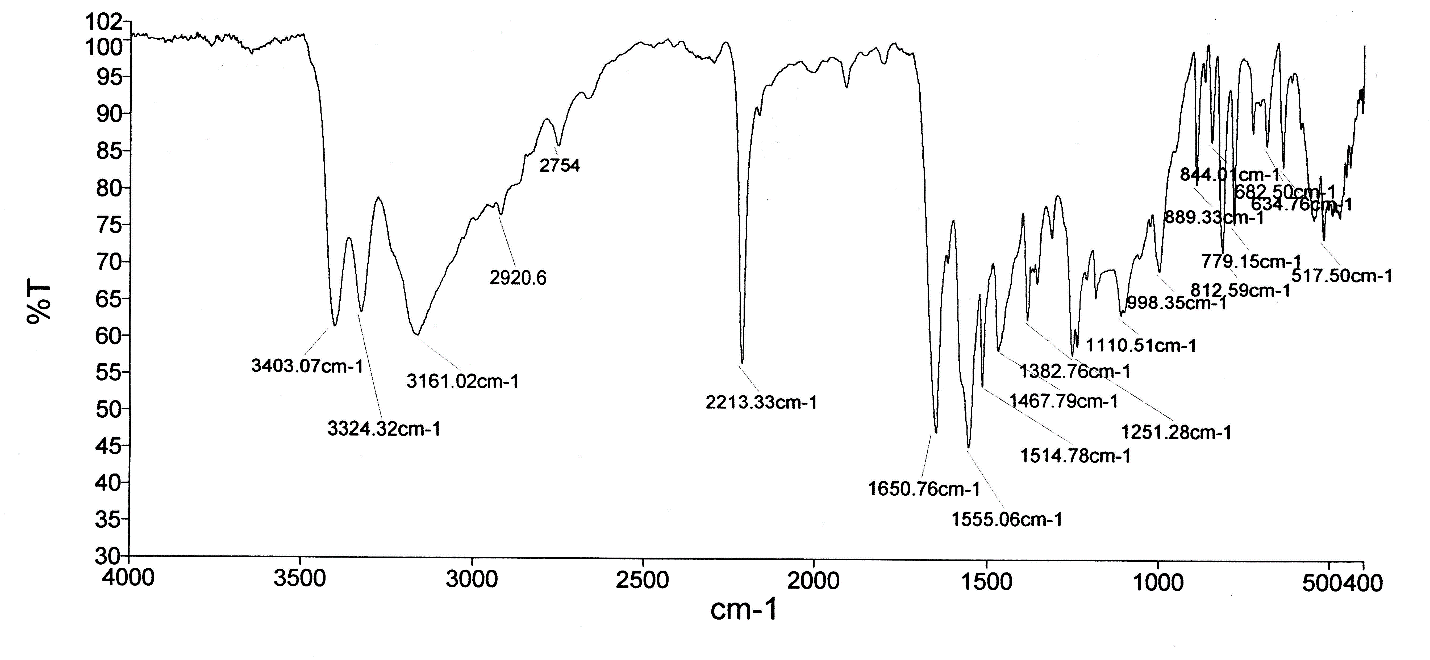


**Figure 70S.** ^1^H NMR spectrum of *5-amino-7-(3-hydroxyphenyl)-[1,2,4]triazolo[1,5-a]pyrimidine-6-carbonitrile (8'h)*


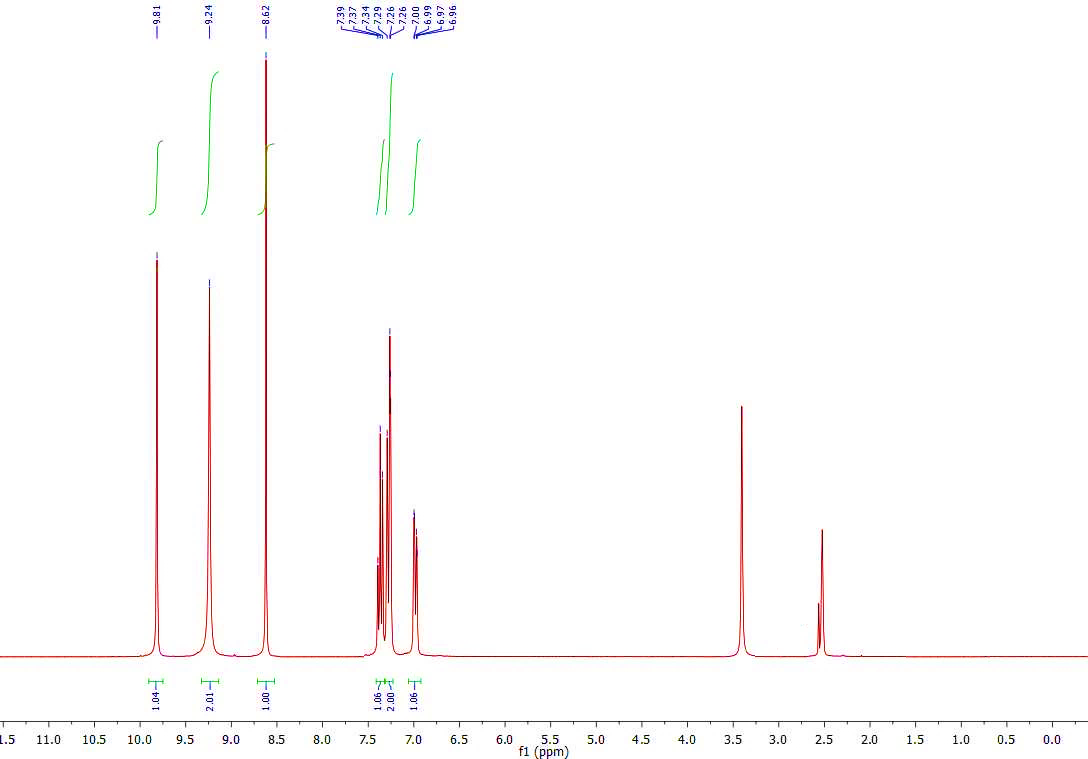

^
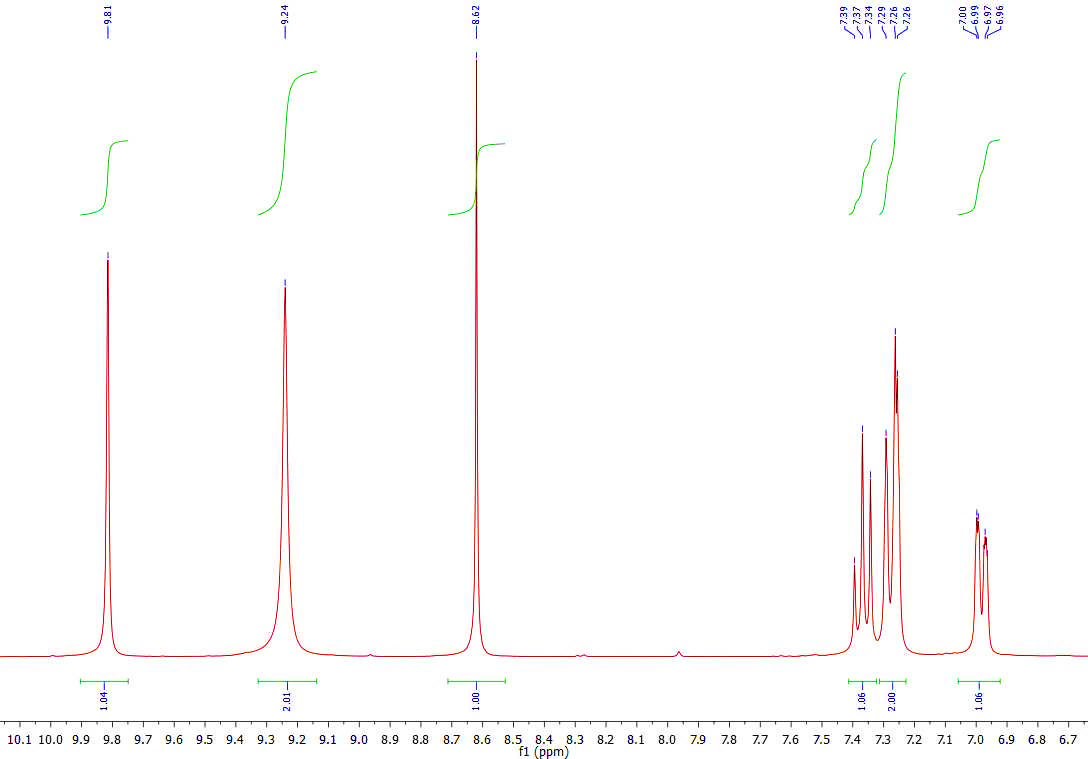
^

**Figure 71S.** ^13^C NMR spectrum of *5-amino-7-(3-hydroxyphenyl)-[1,2,4]triazolo[1,5-a]pyrimidine-6-carbonitrile (8'h)*


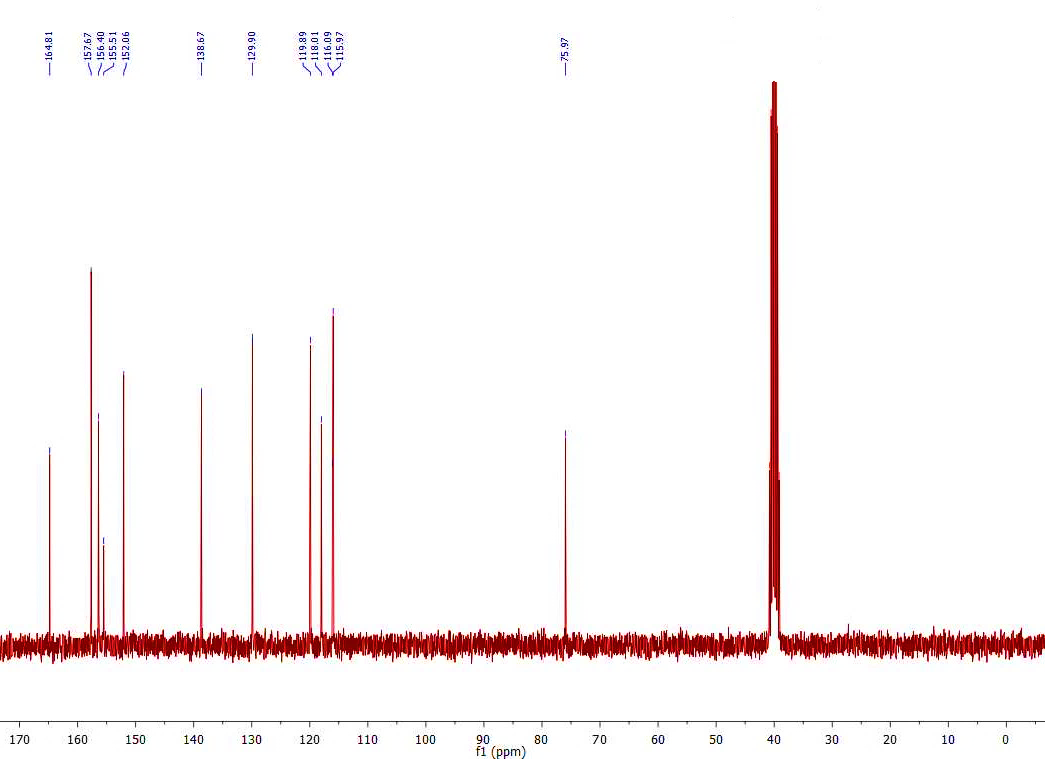


**Figure 72S.** FT-IR spectrum of *7-phenyl-7,12-dihydro-5H-isochromeno[4,3-d][1,2,4]triazolo[1,5-a]pyrimidin-5-one (9a)*

^^
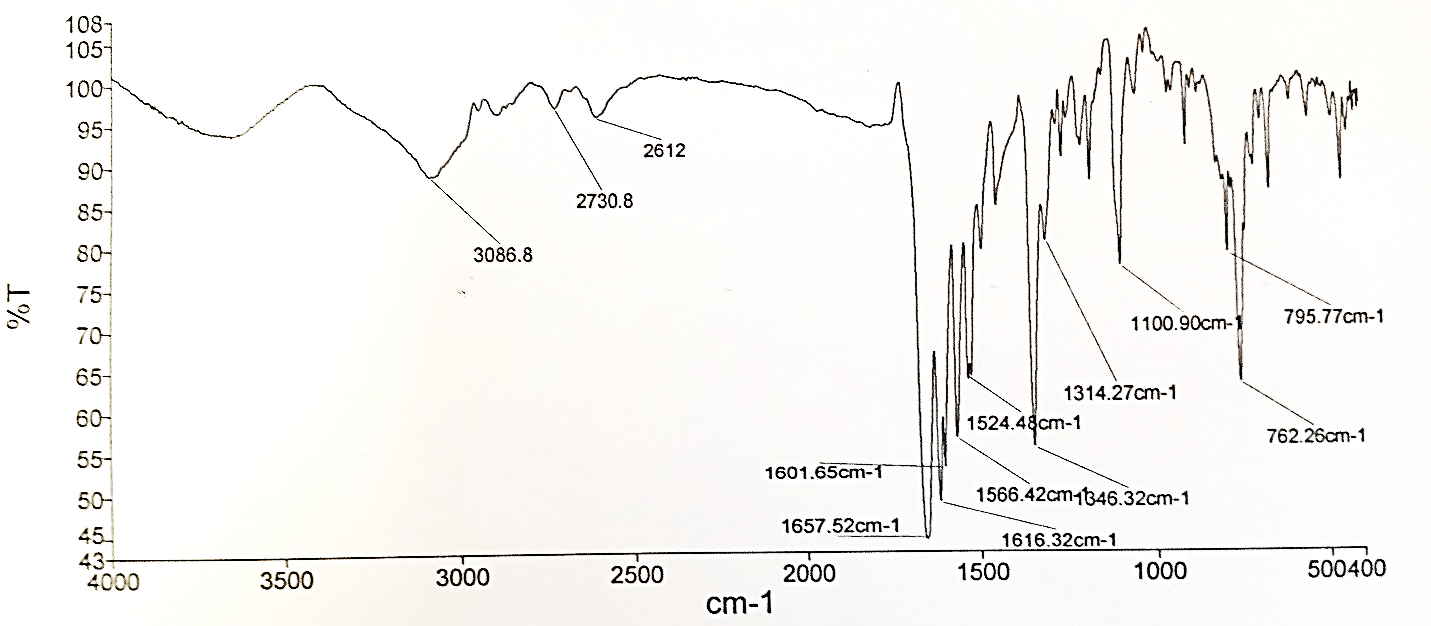


**Figure 73S.** ^1^H NMR spectrum of *7-phenyl-7,12-dihydro-5H-isochromeno[4,3-d][1,2,4]triazolo[1,5-a]pyrimidin-5-one (9a)*

^
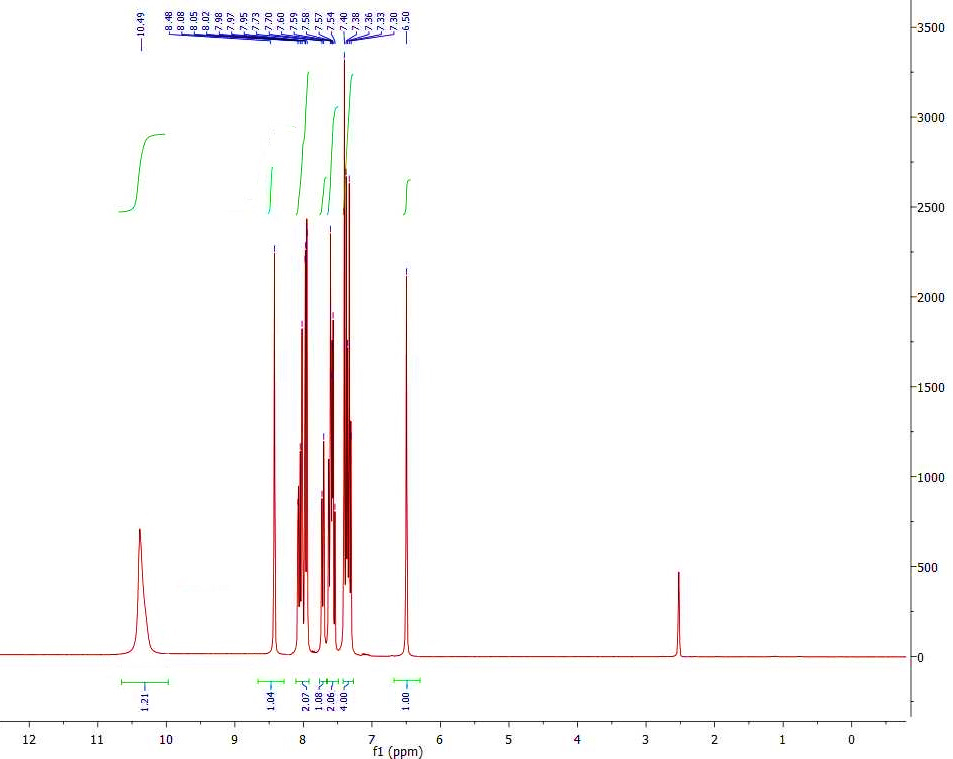
^

**Figure 74S.** ^13^C NMR spectrum of *7-phenyl-7,12-dihydro-5H-isochromeno[4,3-d][1,2,4]triazolo[1,5-a]pyrimidin-5-one (9a)*

^^
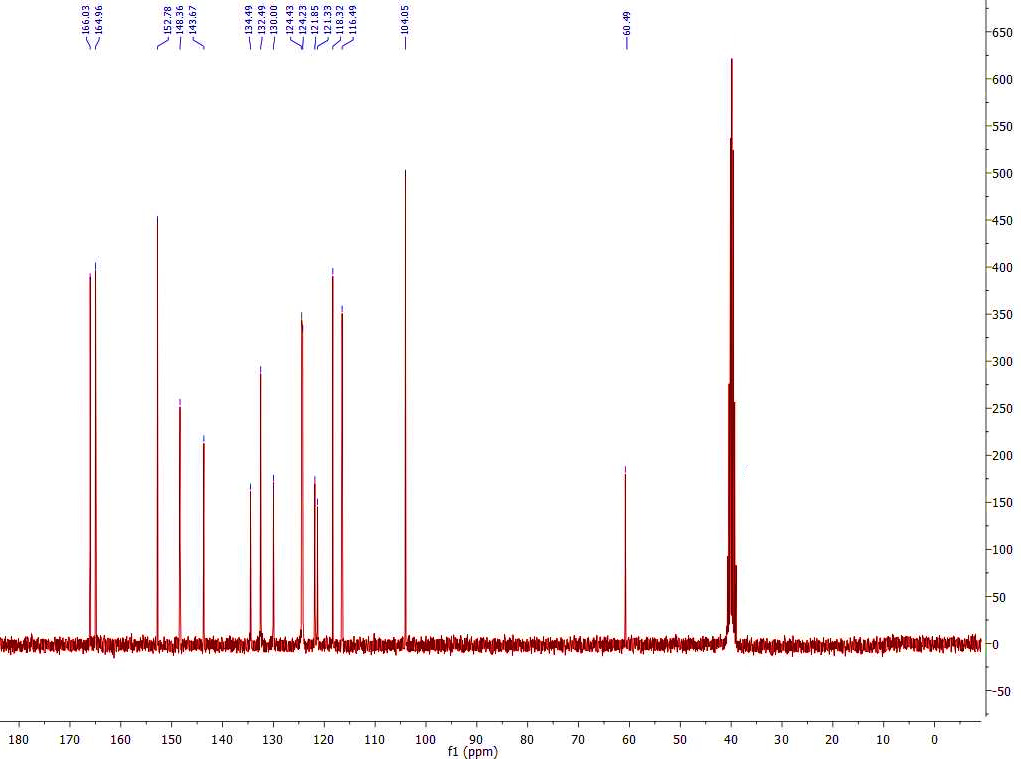


**Figure 75S.** FT-IR spectrum of *7-(4-fluorophenyl)-7,12-dihydro-5H-isochromeno[4,3-d][1,2,4]triazolo[1,5-a]pyrimidin-5-one (9b)*


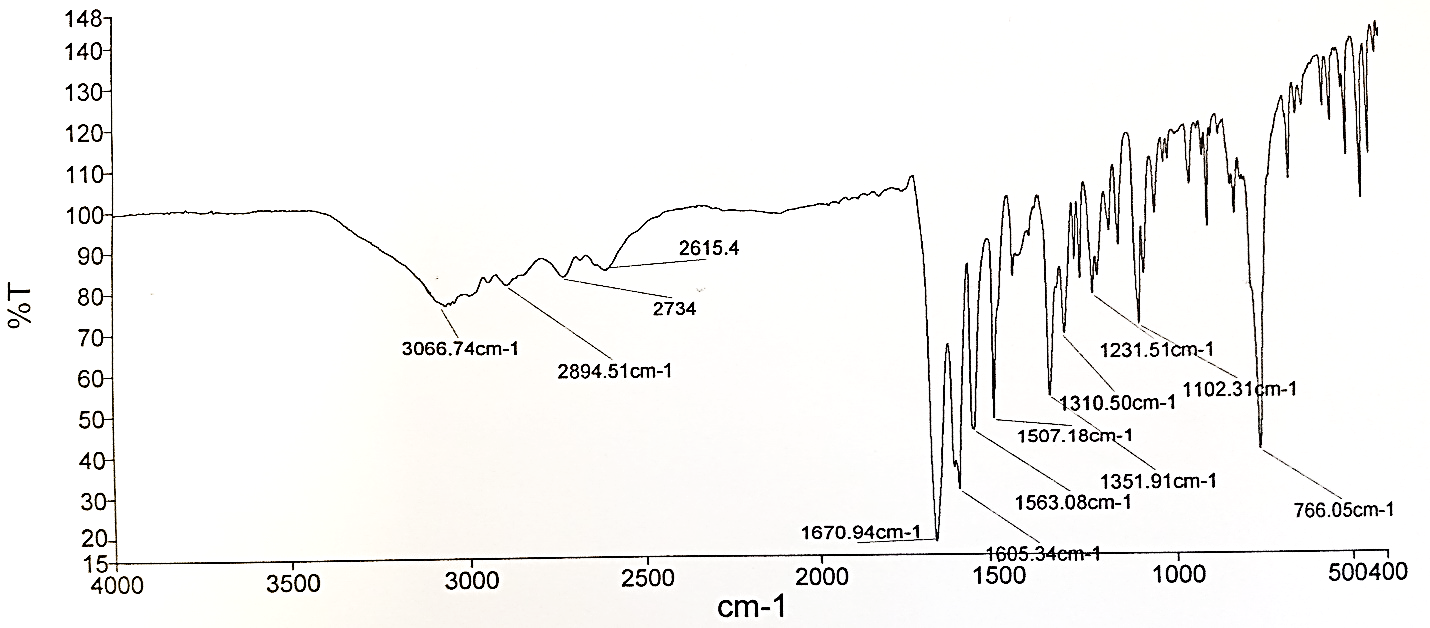


**Figure 76S.** ^1^H NMR spectrum of *7-(4-fluorophenyl)-7,12-dihydro-5H-isochromeno[4,3-d][1,2,4]triazolo[1,5-a]pyrimidin-5-one (9b)*


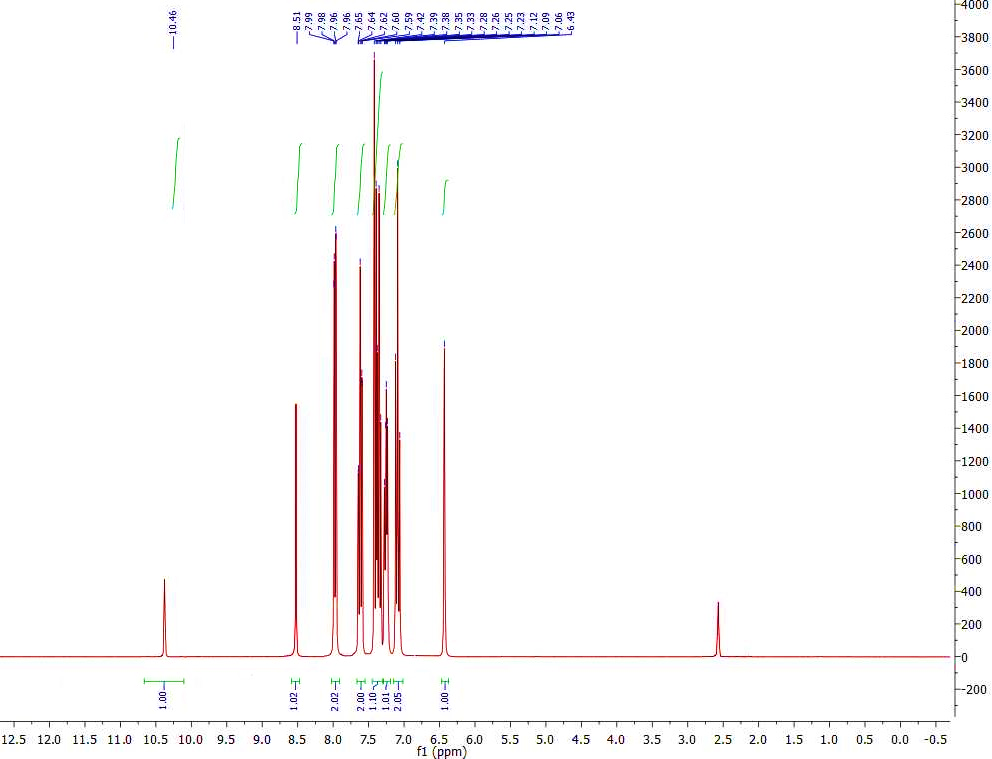


**Figure 77S.** ^13^C NMR spectrum of *7-(4-fluorophenyl)-7,12-dihydro-5H-isochromeno[4,3-d][1,2,4]triazolo[1,5-a]pyrimidin-5-one (9b)*


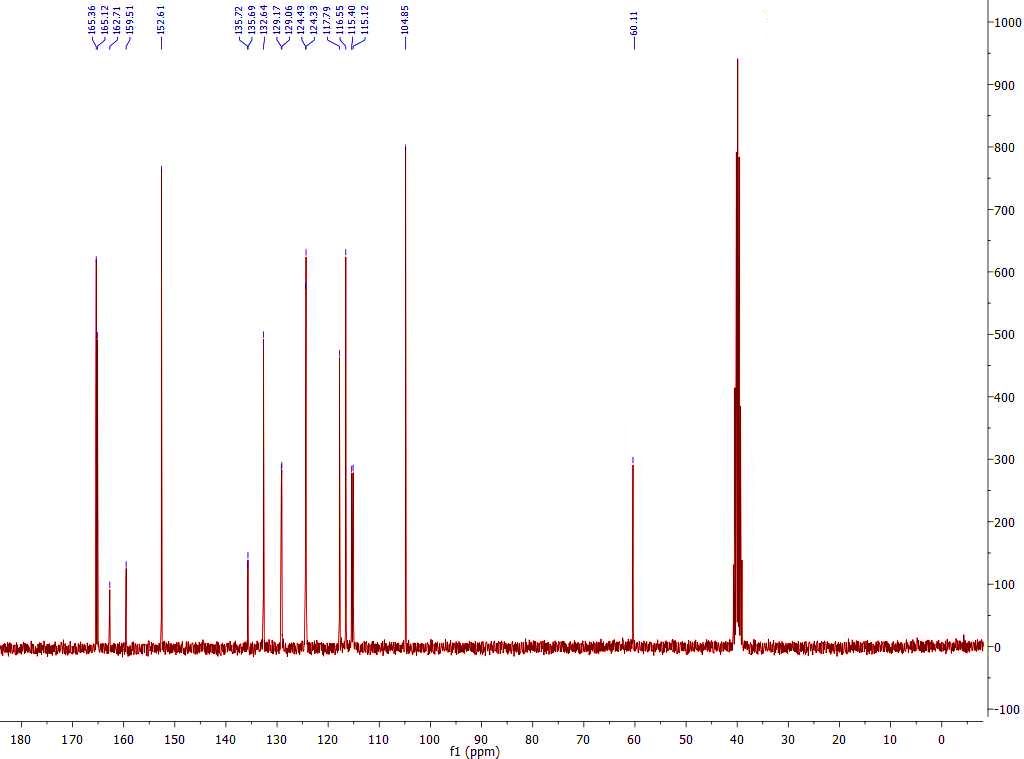


**Figure 78S.** FT-IR spectrum of *7-(4-bromophenyl)-7,12-dihydro-5H-isochromeno[4,3-d][1,2,4]triazolo[1,5-a]pyrimidin-5-one (9c)*

^^
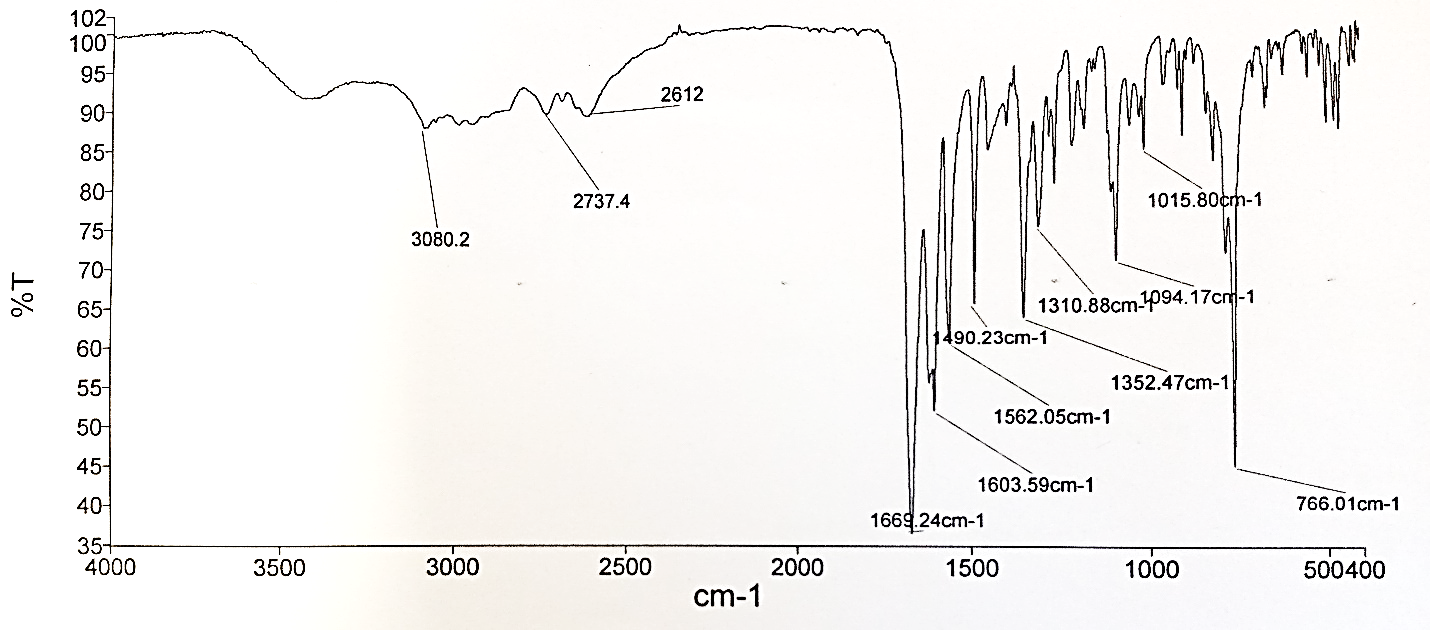


**Figure 79S.** ^1^H NMR spectrum of *7-(4-bromophenyl)-7,12-dihydro-5H-isochromeno[4,3-d][1,2,4]triazolo[1,5-a]pyrimidin-5-one (9c)*


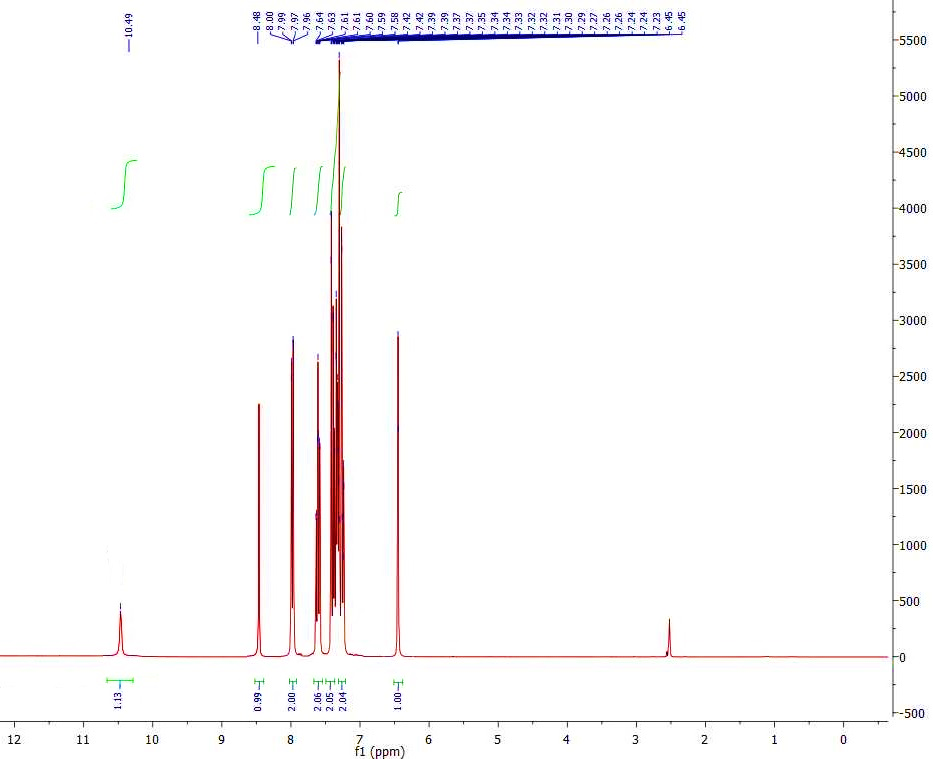


**Figure 80S.** ^13^C NMR spectrum of *7-(4-bromophenyl)-7,12-dihydro-5H-isochromeno[4,3-d][1,2,4]triazolo[1,5-a]pyrimidin-5-one (9c)*


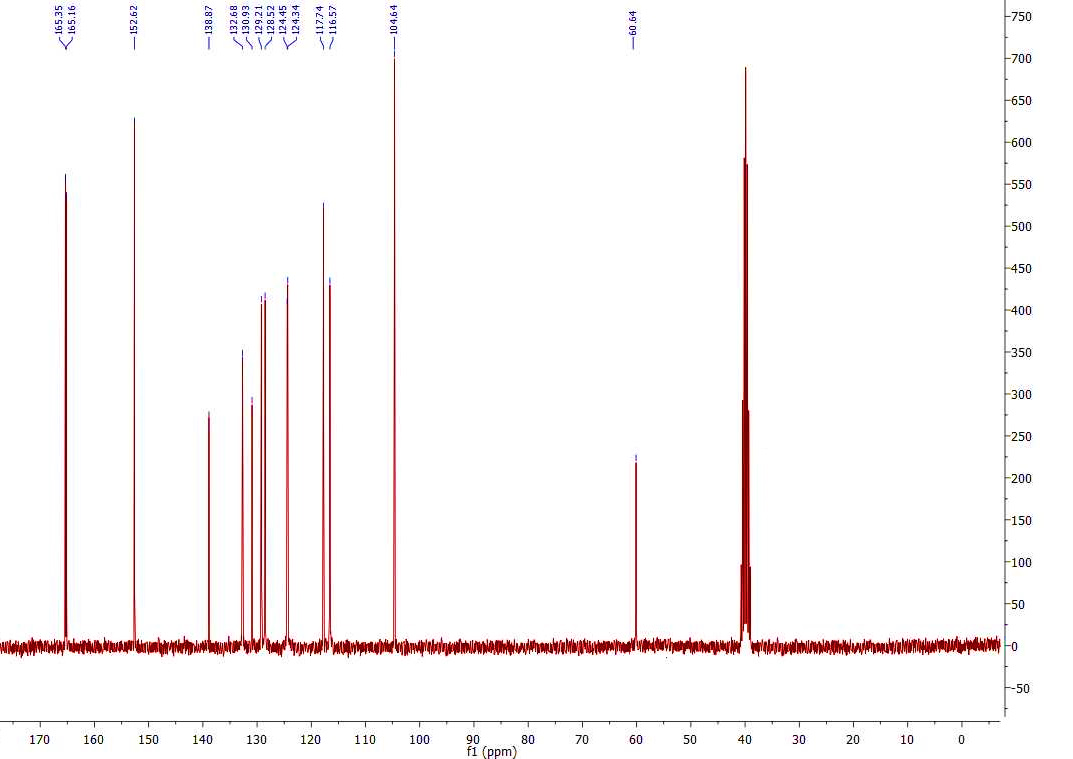


**Figure 81S.** FT-IR spectrum of *7-(3,4-dimethoxyphenyl)-7,12-dihydro-5H-isochromeno[4,3-d][1,2,4]triazolo[1,5-a]pyrimidin-5-one (9d)*


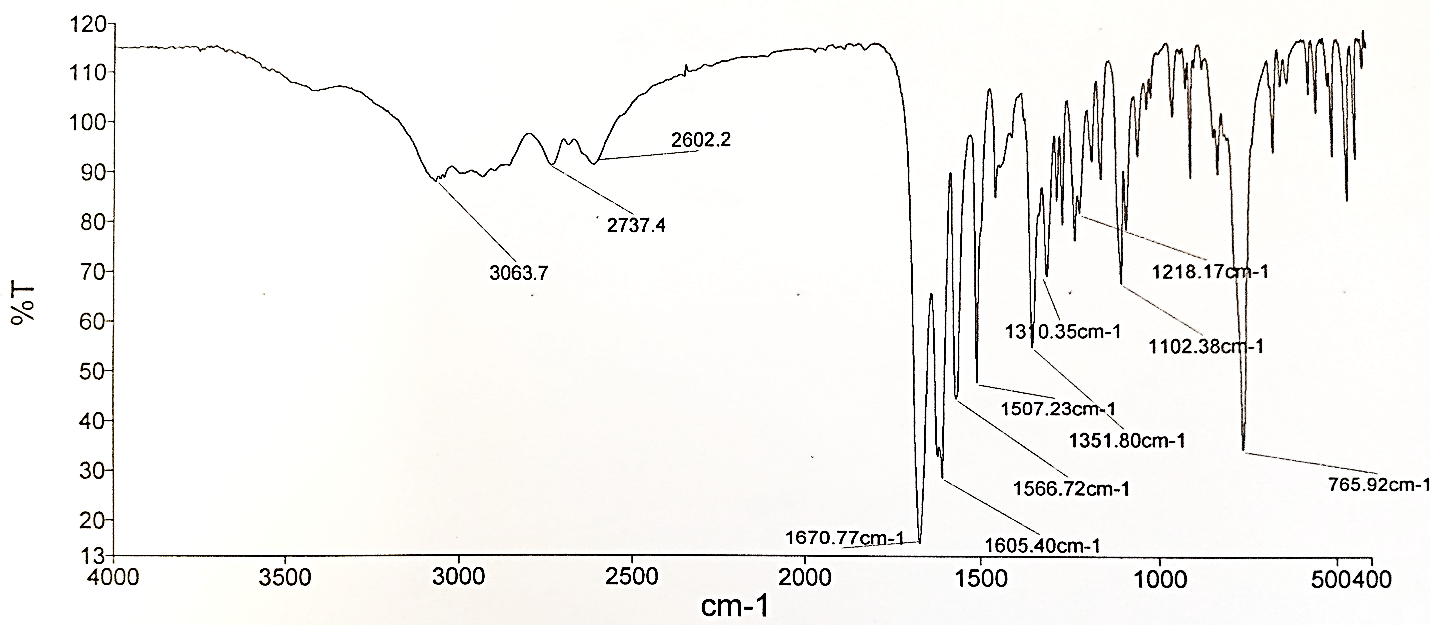


**Figure 82S.** ^1^H NMR spectrum of *7-(3,4-dimethoxyphenyl)-7,12-dihydro-5H-isochromeno[4,3-d][1,2,4]triazolo[1,5-a]pyrimidin-5-one (9d)*


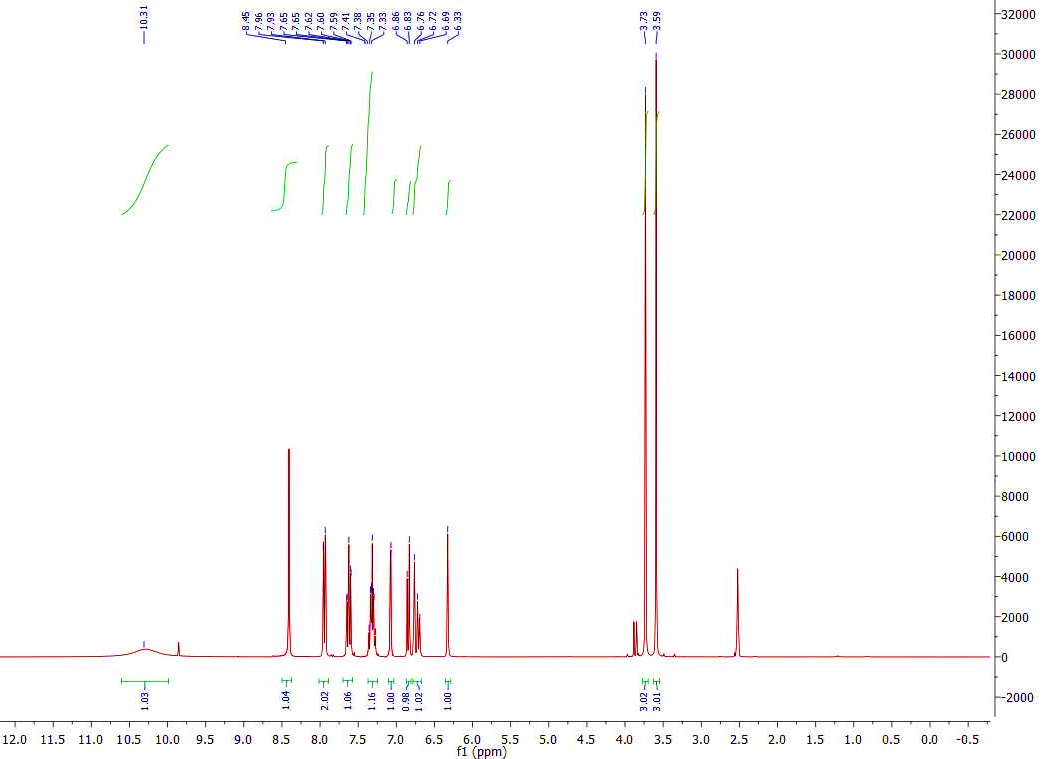


**Figure 83S.** ^13^C NMR spectrum of *7-(3,4-dimethoxyphenyl)-7,12-dihydro-5H-isochromeno[4,3-d][1,2,4]triazolo[1,5-a]pyrimidin-5-one (9d)*


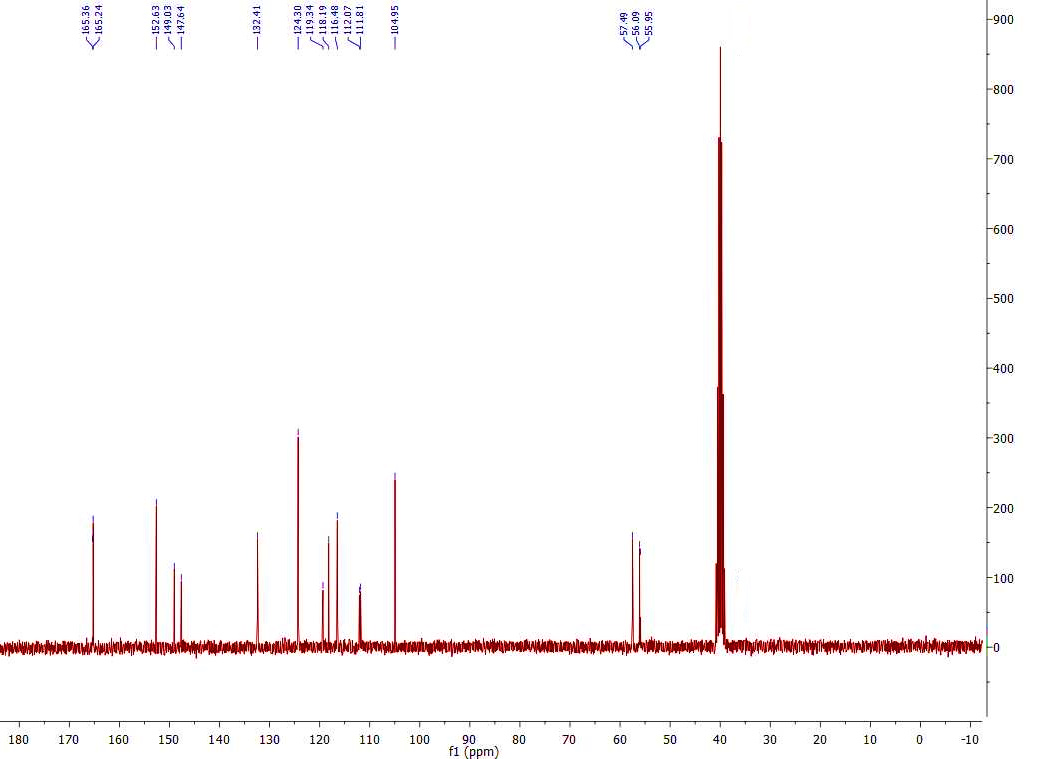


**Figure 84S.** FT-IR spectrum of *7-([1,1'-biphenyl]-4-yl)-7,12-dihydro-5H-isochromeno[4,3-d][1,2,4]triazolo[1,5-a]pyrimidin-5-one (9e)*


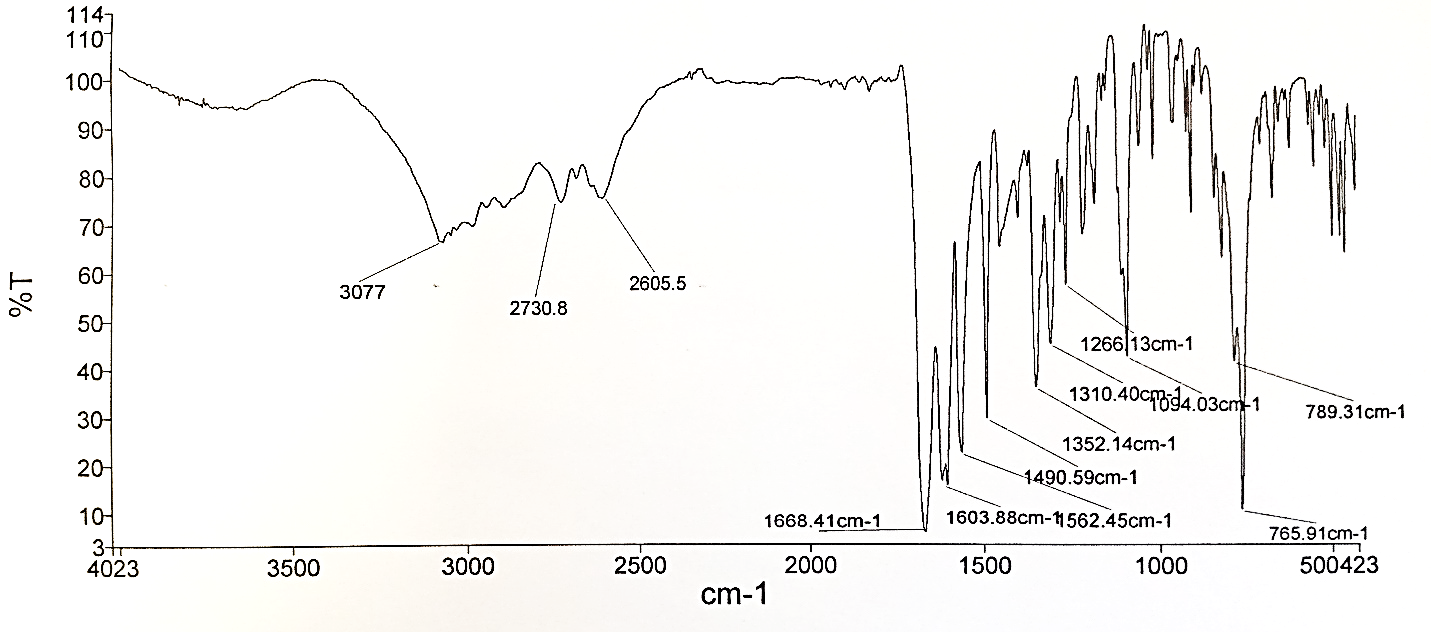


**Figure 85S.** ^1^H NMR spectrum of *7-([1,1'-biphenyl]-4-yl)-7,12-dihydro-5H-isochromeno[4,3-d][1,2,4]triazolo[1,5-a]pyrimidin-5-one (9e)*


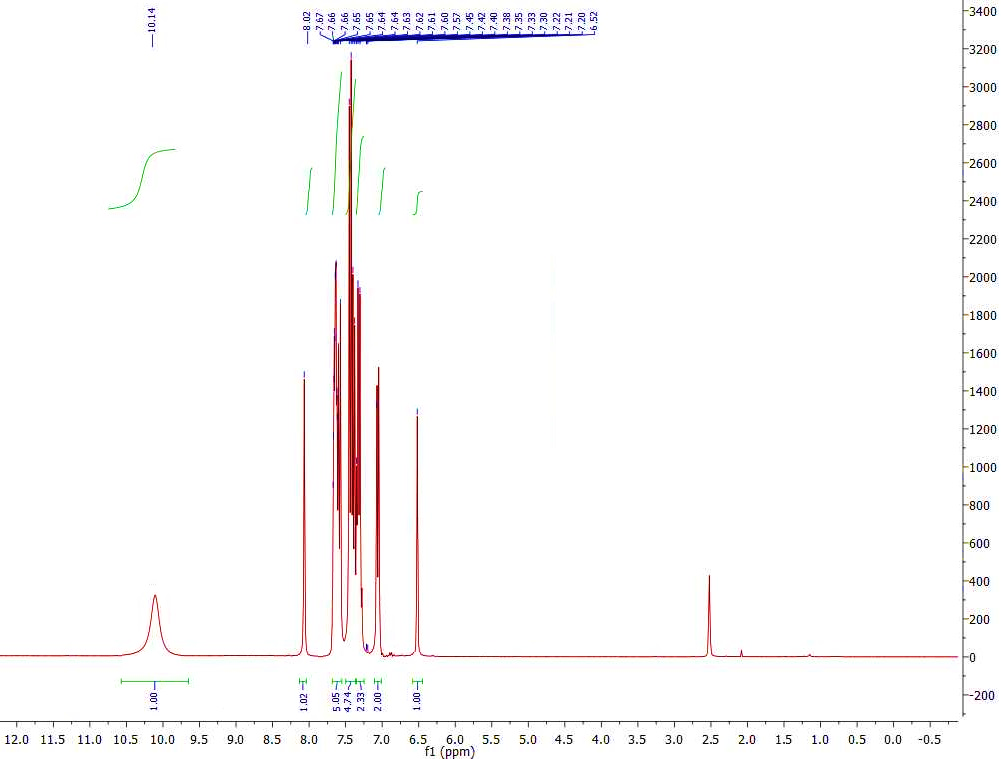


**Figure 86S.** ^13^C NMR spectrum of *7-([1,1'-biphenyl]-4-yl)-7,12-dihydro-5H-isochromeno[4,3-d][1,2,4]triazolo[1,5-a]pyrimidin-5-one (9e)*


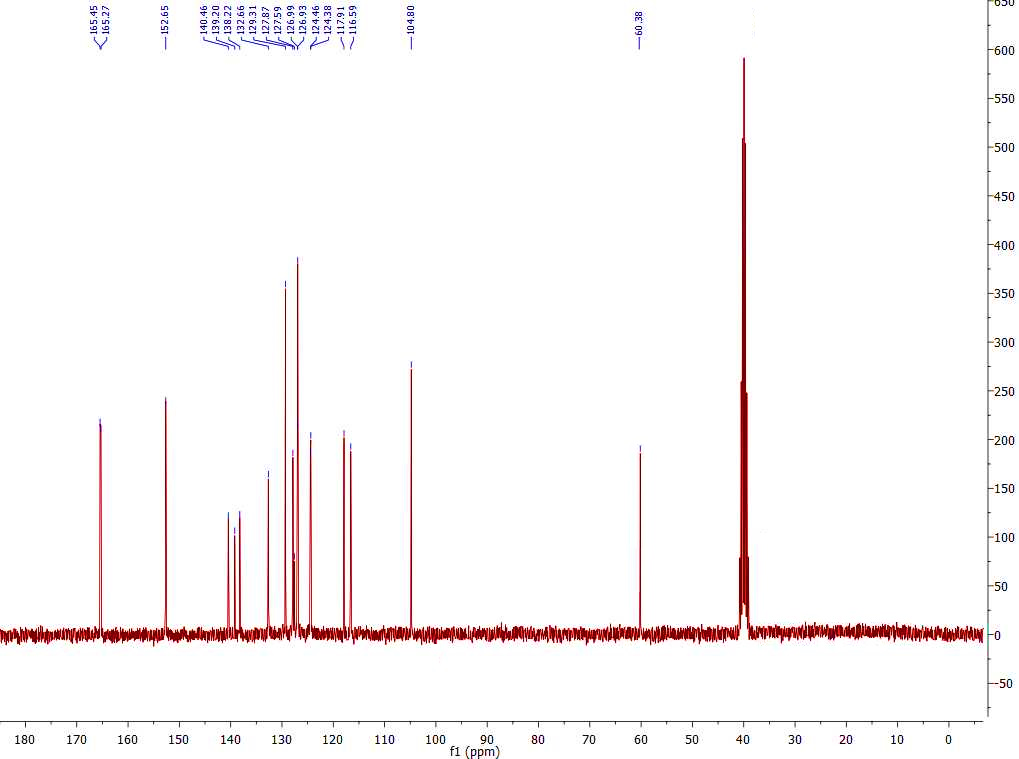


**Figure 87S.** FT-IR spectrum of *2-(1H-indol-3-yl)-4-phenyl-5,6,7,8-tetrahydroquinoline-3-carbonitrile (10a)*

^
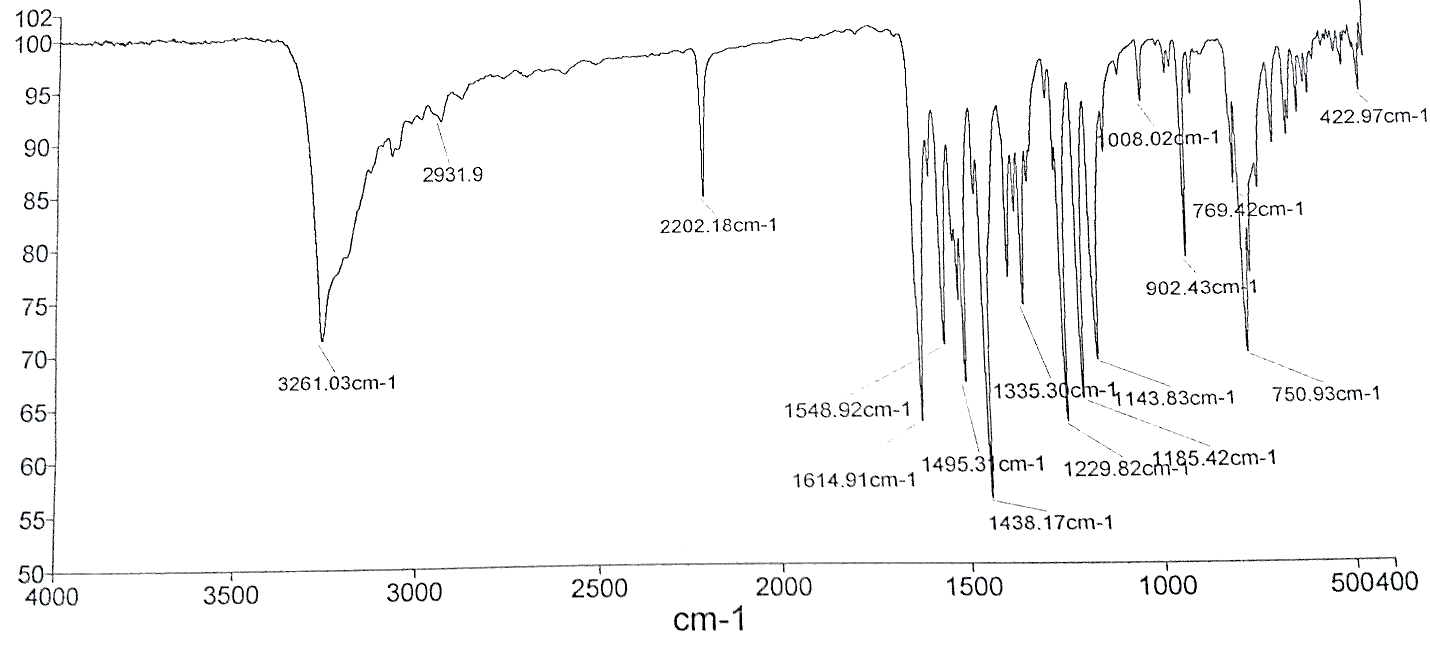
^

**Figure 88S.** ^1^H NMR spectrum of *2-(1H-indol-3-yl)-4-phenyl-5,6,7,8-tetrahydroquinoline-3-carbonitrile (10a)*


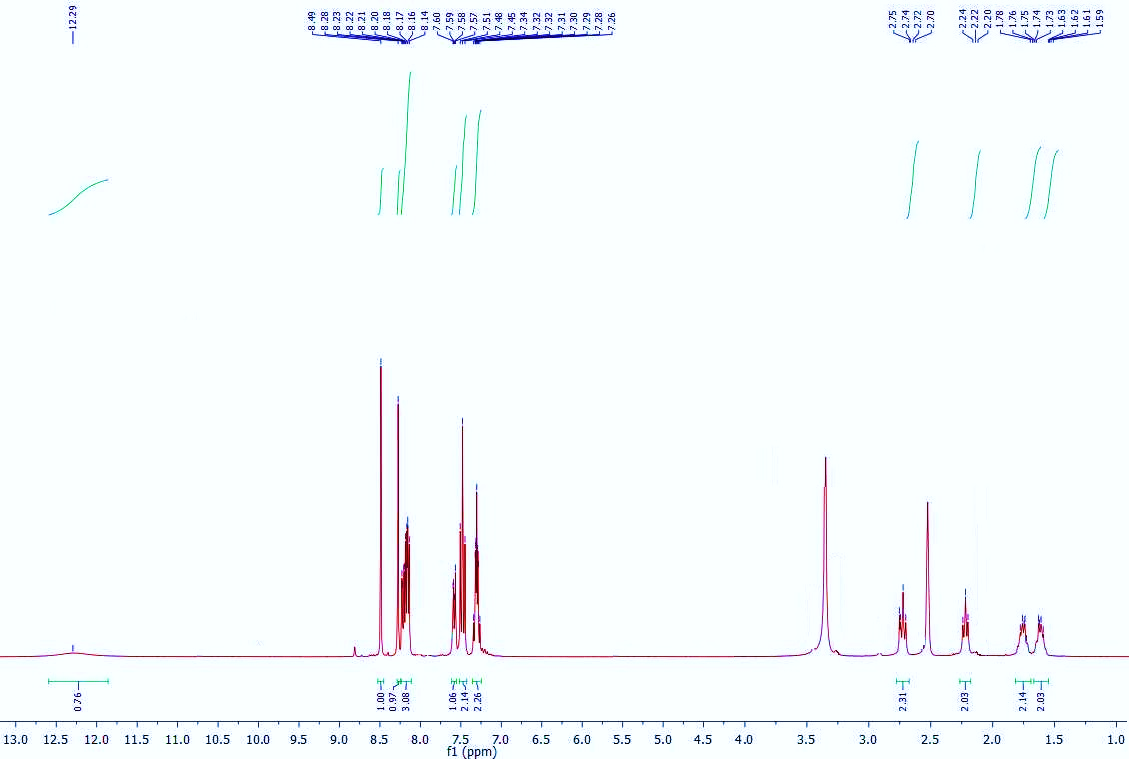


**Figure 89S.** ^13^C NMR spectrum of *2-(1H-indol-3-yl)-4-phenyl-5,6,7,8-tetrahydroquinoline-3-carbonitrile (10a)*


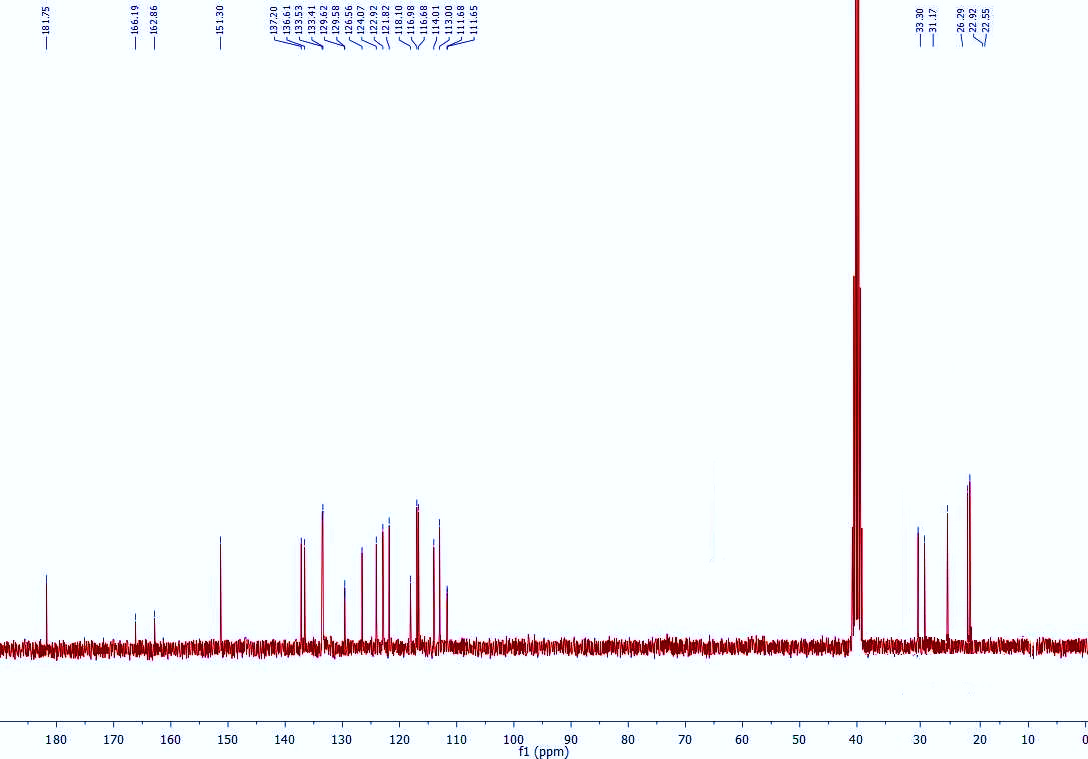


**Figure 90S.** FT-IR spectrum of *2-(1H-indol-3-yl)-4-(naphthalen-2-yl)-5,6,7,8-tetrahydroquinoline-3-carbonitrile (10b)*

^
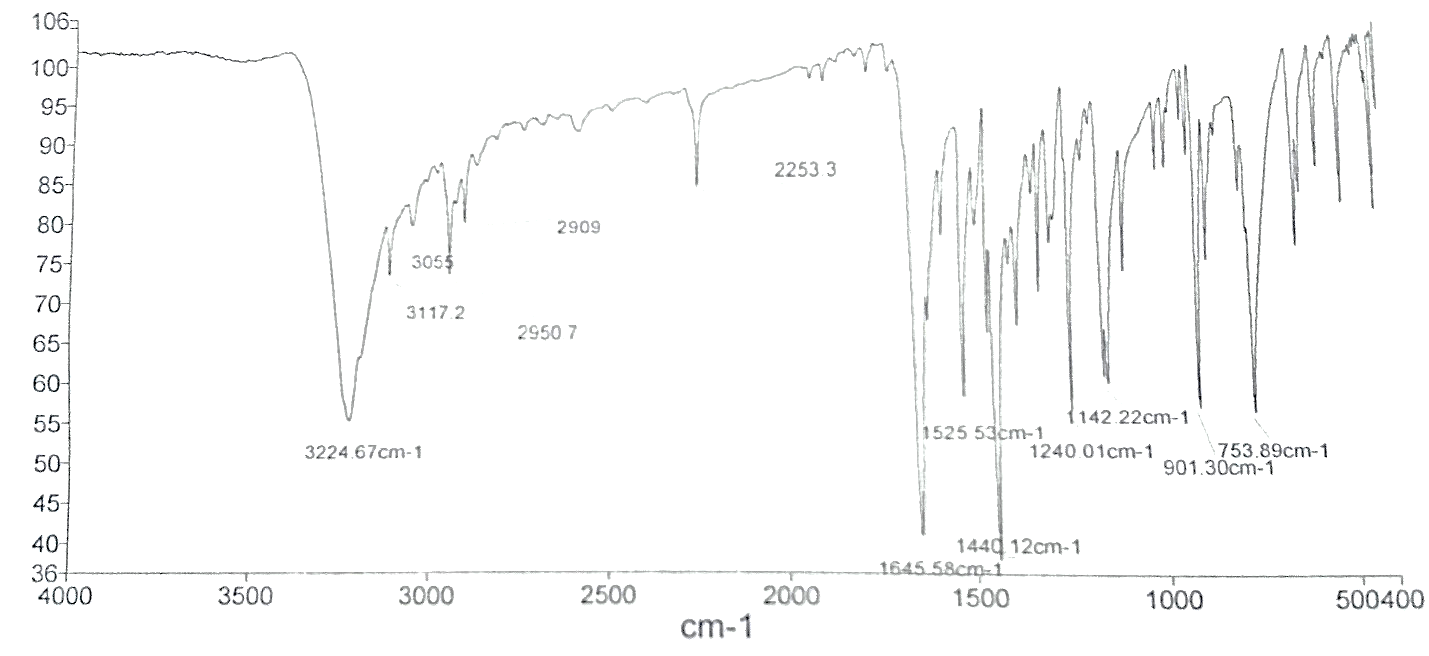
^

**Figure 91S.** ^1^H NMR spectrum of *2-(1H-indol-3-yl)-4-(naphthalen-2-yl)-5,6,7,8-tetrahydroquinoline-3-carbonitrile (10b)*


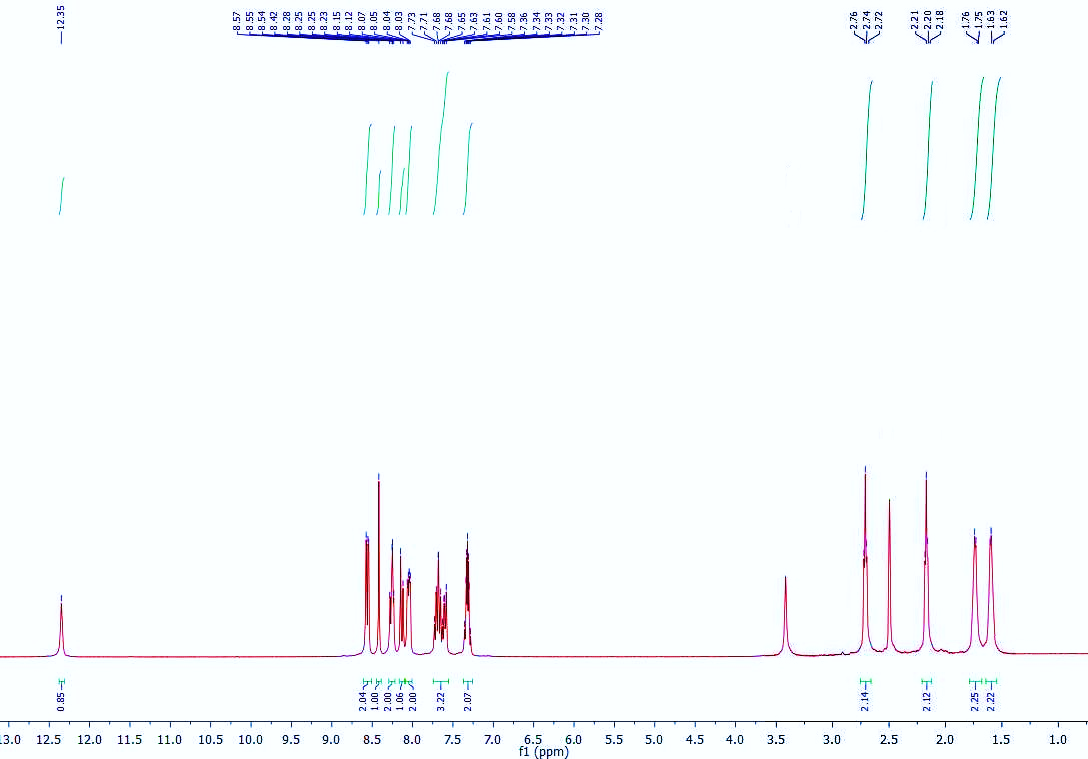


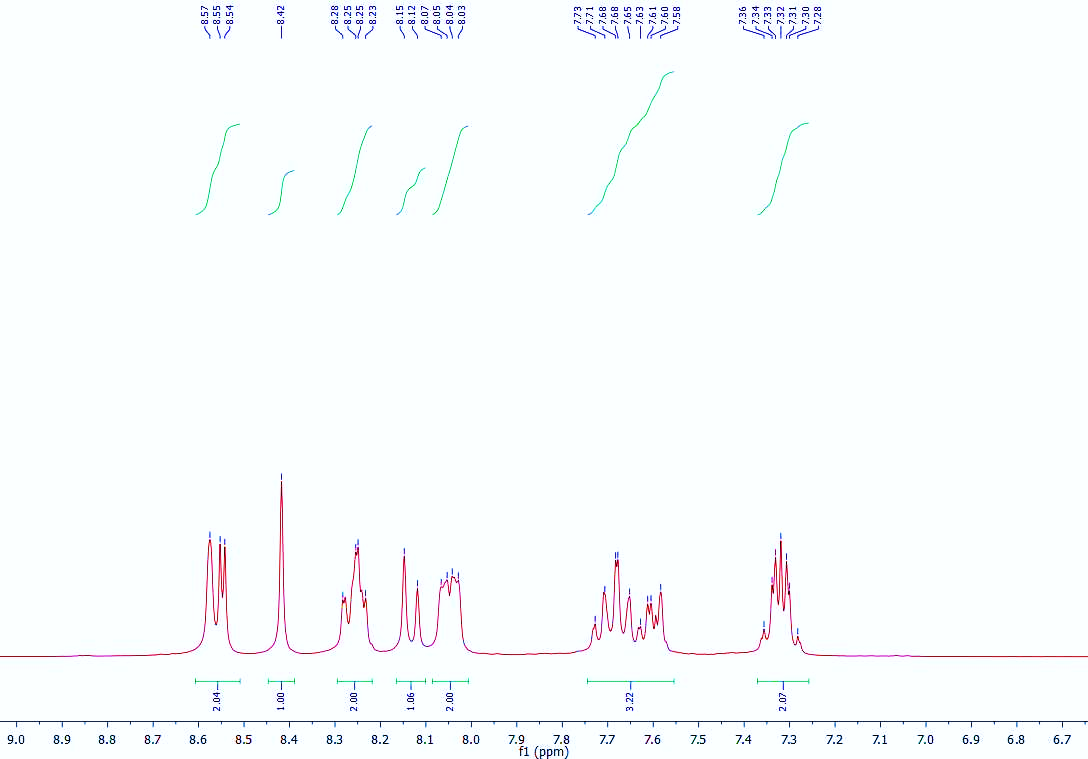


**Figure 92S.** ^13^C NMR spectrum of *2-(1H-indol-3-yl)-4-(naphthalen-2-yl)-5,6,7,8-tetrahydroquinoline-3-carbonitrile (10b)*


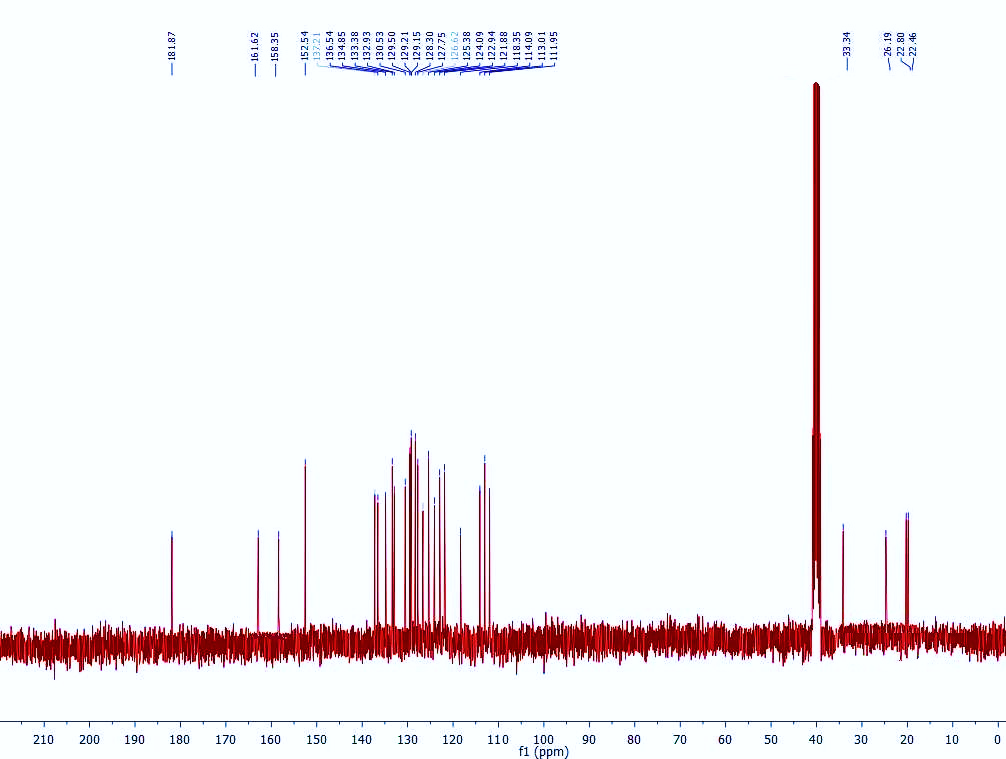


**Figure 93S.** FT-IR spectrum of *2,4-di(1H-indol-3-yl)-5,6,7,8-tetrahydroquinoline-3-carbonitrile (10c)*

^
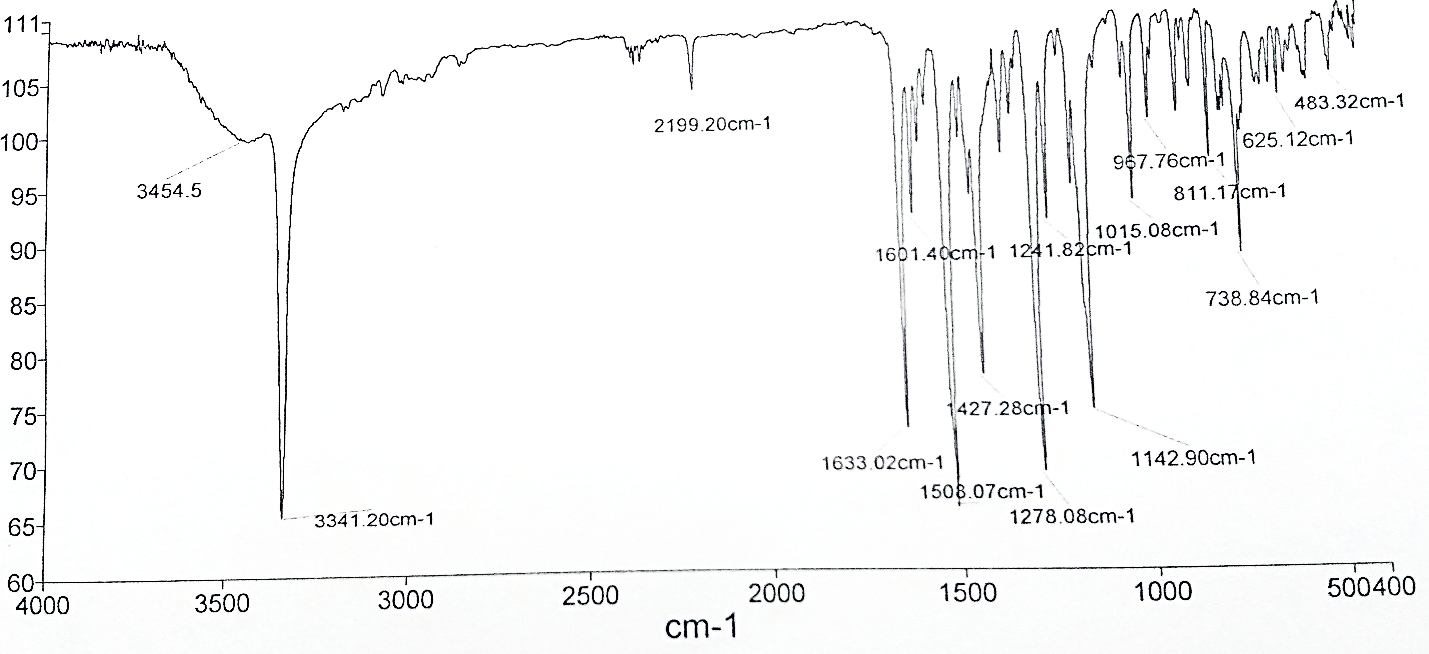
^

**Figure 94S.** ^1^H NMR spectrum of *2,4-di(1H-indol-3-yl)-5,6,7,8-tetrahydroquinoline-3-carbonitrile (10c)*


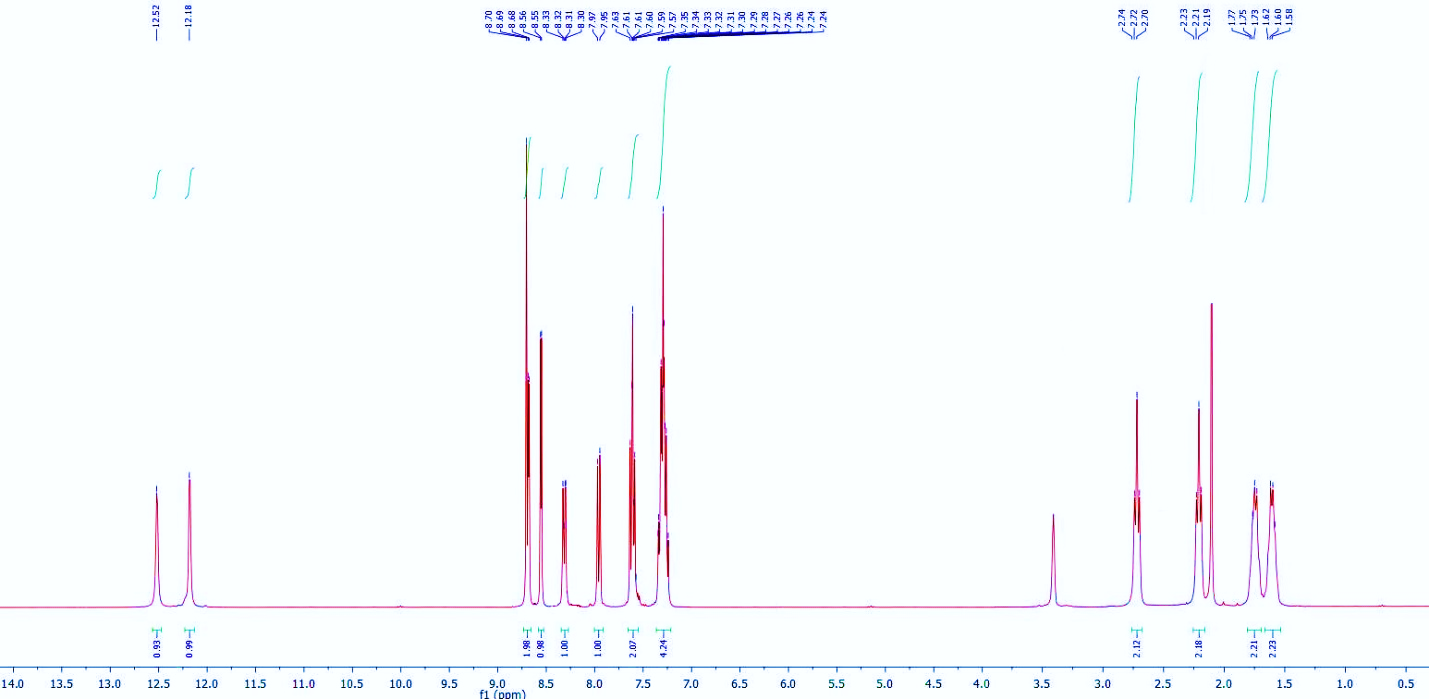


**Figure 95S.** ^13^C NMR spectrum of *2,4-di(1H-indol-3-yl)-5,6,7,8-tetrahydroquinoline-3-carbonitrile (10c)*


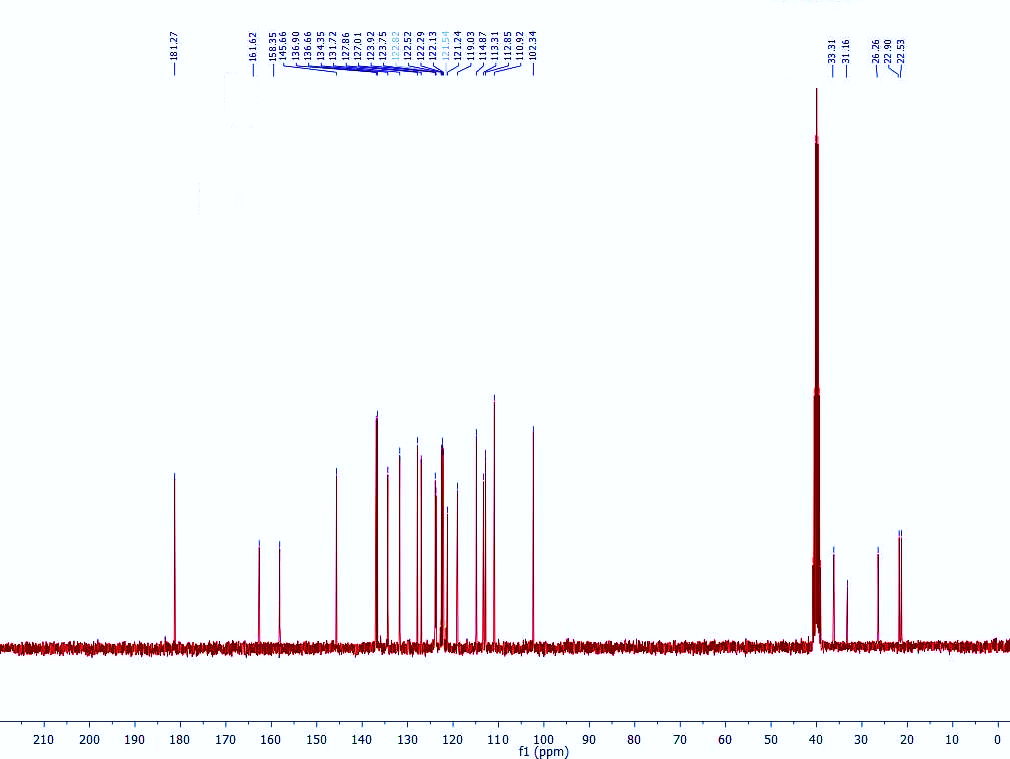


**Figure 96S.** FT-IR spectrum of *2-(1H-indol-3-yl)-4-(naphthalen-1-yl)-5,6,7,8-tetrahydroquinoline-3-carbonitrile (10d)*

^
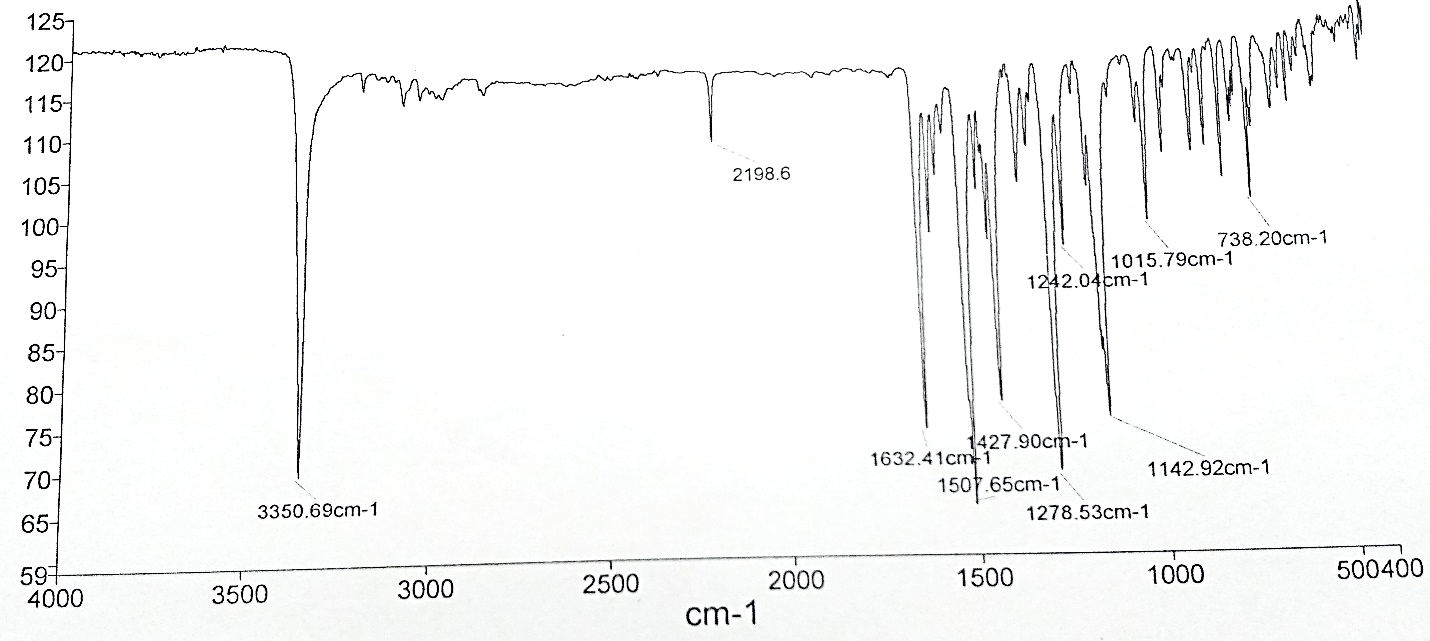
^

**Figure 97S.** ^1^H NMR spectrum of *2-(1H-indol-3-yl)-4-(naphthalen-1-yl)-5,6,7,8-tetrahydroquinoline-3-carbonitrile (10d)*


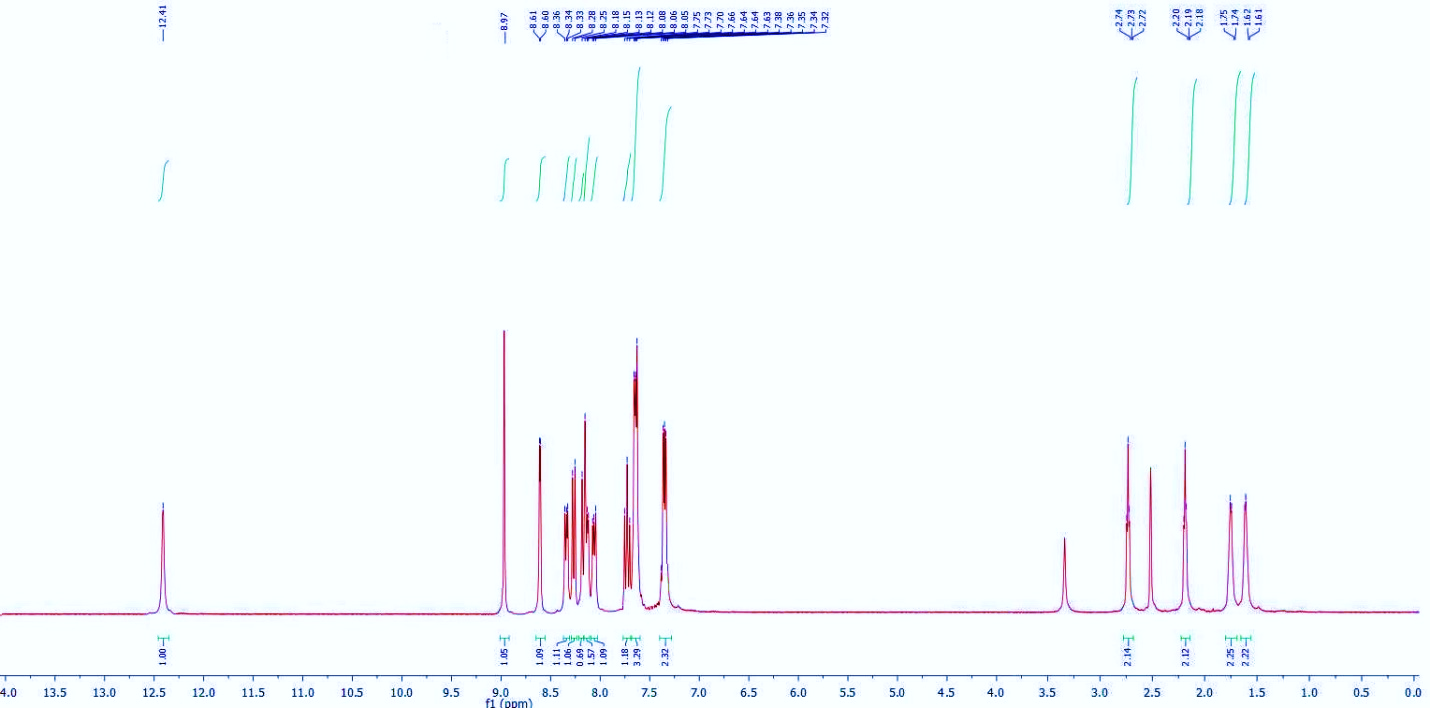


**Figure 98S.** ^13^C NMR spectrum of *2-(1H-indol-3-yl)-4-(naphthalen-1-yl)-5,6,7,8-tetrahydroquinoline-3-carbonitrile (10d)*


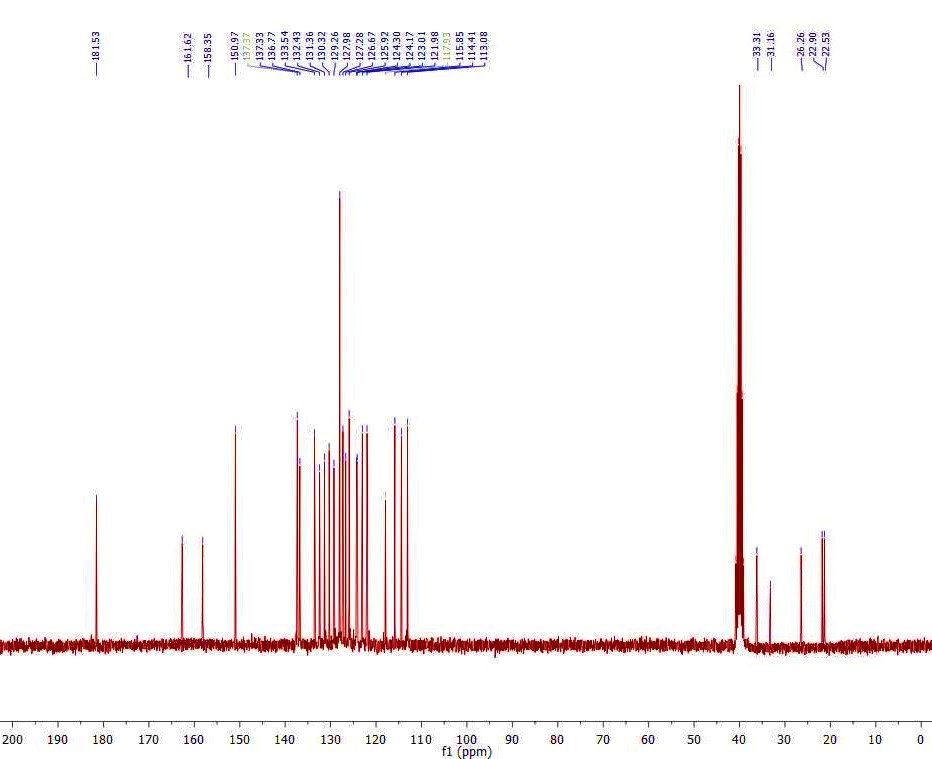


**Figure 99S.** CHN elemental analysis of compounds **8e-8g**, **8'e-8'h**, and **10a-10d**

**Figure 99S.** *Continues*

**Figure 100S.** CHN elemental analysis of compound **A**

**Figure 101S.** CHN elemental analysis of compound **B**

**Figure 102S.** CHN elemental analysis of compound **C**

**Figure 103S.** CHN elemental analysis of **[(VO)TPP][(TCM)_4_]**

**Figure 104S.** ICP-OES analysis of **[(VO)TPP][(TCM)_4_]**

**Figure 105S.** ICP-OES analysis of recycled **[(VO)TPP][(TCM)_4_]**
